# An essential and highly selective protein import pathway encoded by nucleus-forming phage

**DOI:** 10.1073/pnas.2321190121

**Published:** 2024-04-30

**Authors:** Chase J. Morgan, Eray Enustun, Emily G. Armbruster, Erica A. Birkholz, Amy Prichard, Taylor Forman, Ann Aindow, Wichanan Wannasrichan, Sela Peters, Koe Inlow, Isabelle L. Shepherd, Alma Razavilar, Vorrapon Chaikeeratisak, Benjamin A. Adler, Brady F. Cress, Jennifer A. Doudna, Kit Pogliano, Elizabeth Villa, Kevin D. Corbett, Joe Pogliano

**Affiliations:** ^a^School of Biological Sciences, Division of Molecular Biology, University of California San Diego, La Jolla, CA 92093; ^b^Department of Biochemistry, Faculty of Science, Chulalongkorn University, Bangkok, Thailand 10330; ^c^California Institute for Quantitative Biosciences, University of California, Berkeley, CA 94720; ^d^Innovative Genomics Institute, University of California, Berkeley, CA 94720; ^e^Department of Molecular and Cell Biology, University of California, Berkeley, CA 94720; ^f^Department of Chemistry, University of California, Berkeley, CA 94720; ^g^HHMI, University of California, Berkeley, CA 94720; ^h^Environmental Genomics and Systems Biology Division, Lawrence Berkeley National Laboratory, Berkeley, CA 94720; ^i^Molecular Biophysics and Integrated Bioimaging Division, Lawrence Berkeley National Laboratory, Berkeley, CA 94720; ^j^HHMI, University of California San Diego, La Jolla, CA 92093; ^k^Department of Cellular and Molecular Medicine, University of California San Diego, La Jolla, CA 92093

**Keywords:** phage, protein trafficking, phage nucleus

## Abstract

Targeting proteins to specific subcellular destinations is essential in prokaryotes, eukaryotes, and the viruses that infect them. Chimalliviridae phages encapsulate their genomes in a nucleus-like replication compartment composed of the protein chimallin (ChmA) that excludes ribosomes and decouples transcription from translation. These phages selectively partition proteins between the phage nucleus and the bacterial cytoplasm. Currently, the genes and signals that govern selective protein import into the phage nucleus are unknown. Here, we identify two components of this protein import pathway: a species-specific surface-exposed region of a phage intranuclear protein required for nuclear entry and a conserved protein, PicA (Protein importer of chimalliviruses A), that facilitates cargo protein trafficking across the phage nuclear shell. We also identify a defective cargo protein that is targeted to PicA on the nuclear periphery but fails to enter the nucleus, providing insight into the mechanism of nuclear protein trafficking. Using CRISPRi-ART protein expression knockdown of PicA, we show that PicA is essential early in the chimallivirus replication cycle. Together, our results allow us to propose a multistep model for the Protein Import Chimallivirus pathway, where proteins are targeted to PicA by amino acids on their surface and then licensed by PicA for nuclear entry. The divergence in the selectivity of this pathway between closely related chimalliviruses implicates its role as a key player in the evolutionary arms race between competing phages and their hosts.

## Significance

The phage nucleus is an enclosed replication compartment built by Chimalliviridae phages that, similar to the eukaryotic nucleus, separates transcription from translation and selectively imports certain proteins. This allows the phage to concentrate proteins required for DNA replication and transcription while excluding DNA-targeting host defense proteins. However, the mechanism of selective trafficking into the phage nucleus is currently unknown. Here, we determine the region of a phage nuclear protein that targets it for nuclear import and identify a conserved, essential nuclear shell-associated protein that plays a key role in this process. This work provides a mechanistic model of selective import into the phage nucleus.

Compartmentalization of the cytoplasm into organelles enclosed by a semipermeable barrier is a hallmark of eukaryotic life. The phage nucleus is a viral organelle produced by phages in the Chimalliviridae family and the first DNA-enclosing viral replication compartment discovered in a prokaryotic cell (1–6). During the chimallivirus infection cycle, the phage genomes replicate within the phage nucleus, which is bounded by a lattice composed mainly of the phage protein chimallin (ChmA) (1–4, 7–9). The ChmA barrier excludes ribosomes, decoupling transcription from translation, so macromolecular transport across the nuclear shell is necessary (1, 3, 4, 7, 10). mRNA must be exported, while many proteins for DNA replication and transcription must be imported. The ChmA lattice is porous to metabolites and nucleotides required for transcription, but these pores are too small to accommodate trafficking of folded macromolecules (7–9). While a putative nuclear shell mRNA transporter was recently discovered (11), no protein transporter has been identified.

Protein import across the ChmA barrier is selective (1–4, 6, 10, 12). Certain phage proteins concentrate in the nucleus including RNA polymerase subunits, a RecA-related recombinase (henceforth RecA), DNA polymerase, and a number of proteins of unknown function (1, 3, 4, 13, 14). In contrast, many phage proteins are excluded and remain in the host cytoplasm including metabolic enzymes like thymidylate kinase and virion structural proteins (1, 3, 4, 13). Many host proteins are also excluded, including DNA-targeting defense proteins such as Cas9 and restriction endonucleases (1, 4, 6, 10, 12), which provides the phage with broad protection against many host defenses. Furthermore, during coinfection of a single cell by two different nucleus-forming phages, the phage nucleus of one species excludes a toxic nuclease produced by the other (15, 16). The high degree of specificity seen in protein localization to the phage nucleus has led to the hypothesis that chimalliviruses encode a selective protein trafficking system (1, 2, 4, 10, 15, 17).

In eukaryotes, proteins can be sorted into organelles including the endoplasmic reticulum, nucleus, mitochondria, and peroxisomes (18–23). In prokaryotes, proteins can be translocated to the periplasm, outer membrane, or secreted outside of the cell (24–31). While the mechanisms underlying these transport systems vary significantly, most rely on a signal sequence encoded within the cargo protein, a means of recognizing that signal, and a protein complex to facilitate the trafficking of the cargo to its destination (24–31). In nucleus-forming phages, it is reasonable to expect that protein trafficking would follow a similar pattern. However, bioinformatic analyses of nuclear proteins have failed to identify any conserved signal sequences.

Experiments with GFPmut1 suggested that residues on the protein’s surface are essential for its nuclear import (10). GFPmut1 is a GFP variant that is imported into the PhiKZ phage nucleus but not the nuclei of other phages, while other GFP variants tested are not imported into any phage nucleus. Import of GFPmut1 is lost if a surface-exposed phenylalanine (F99) is mutated and partially lost if a nearby surface-exposed methionine (M153) is mutated (10), suggesting the signals that target proteins to the nucleus may be encoded in amino acids distant in sequence but located on the same surface in the folded protein. However, the signals needed for targeting endogenous phage proteins to the nucleus remain unidentified.

Here, we use genetic and cell biological approaches to determine the recognition region on a phage protein that targets it for nuclear import and demonstrate that a conserved protein in nucleus-forming phages, which we term PicA (Protein importer of chimalliviruses A), plays a key role in selective nuclear import. PicA is a protein identified to be involved in selective protein trafficking into the phage nucleus, and we show that it is essential for viral reproduction. These results provide insight into how these phages selectively partition proteins between the phage nucleus and the bacterial cytoplasm and establish the PIC pathway as an essential component of the phage replication cycle with direct implications for phage–phage and phage–host competition.

## Results

### Protein Localization to the Phage Nucleus Is Species and Sequence Specific.

**Fig. 1.**
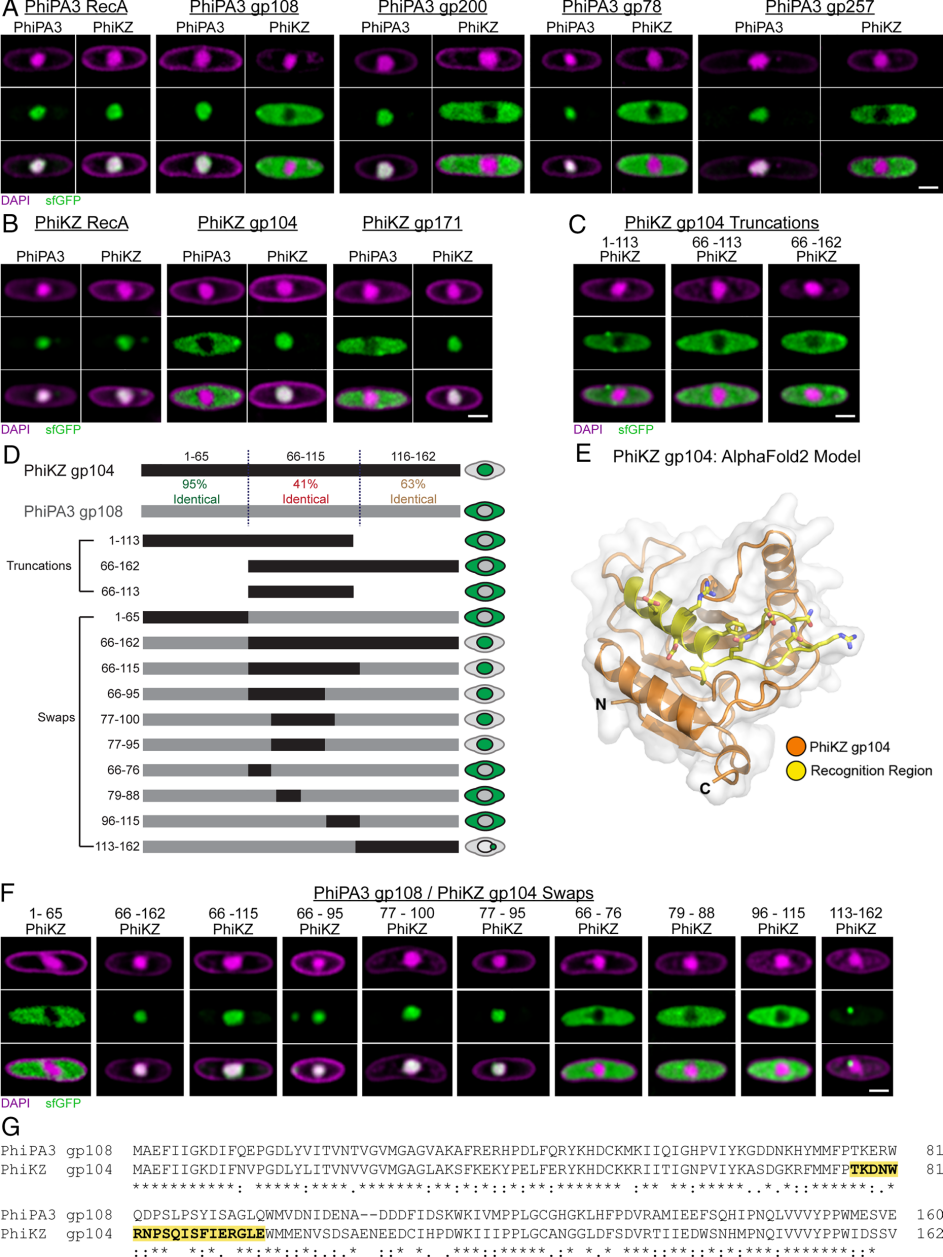
Protein trafficking into the phage nucleus is species and sequence specific. (*A*) *P. aeruginosa* strain PA01 K2733 cells expressing the indicated sfGFP-tagged PhiPA3 nuclear protein infected with either PhiPA3 or PhiKZ. DAPI staining (purple) shows the phage nucleus in the center of the cell and peptidoglycan at the cell periphery. sfGFP (green) shows the localization of the tagged protein either to the phage nucleus or the cytoplasm. (*B*) *P. aeruginosa* cells expressing the indicated sfGFP-tagged PhiKZ nuclear protein infected with either PhiPA3 or PhiKZ. (*C*) *P. aeruginosa* cells expressing the indicated sfGFP-tagged PhiKZ gp104 truncation infected with PhiKZ. (*D*) Schematic depicting the percent conservation between PhiKZ gp104 and PhiPA3 gp108 and the sfGFP-tagged protein constructs expressed in (*D*) and (*E*) with their phenotype indicated on the right. Numbers on the left indicate the amino acids belonging to PhiKZ gp104 in each construct. (*E*) AlphaFold predicted structure of PhiKZ gp104 with the experimentally determined recognition region required for nuclear localization shown in yellow. (*F*) *P. aeruginosa* cells expressing the indicated sfGFP-tagged PhiKZ gp104/PhiPA3 gp108 chimera infected with PhiKZ. All infections were imaged at 30 to 45 mpi. (Scale bars, 1 µm.) All cells are representative of the population. Larger fields of view appear in *SI Appendix*. (*G*) Clustal Omega generated protein sequence alignment of PhiPA3 gp108 and PhiKZ gp104. “*” denotes conserved amino acids. “:” and “.” denote highly similar and weakly similar amino acids, respectively. The sequence of PhiKZ gp104 import recognition region is highlighted in yellow and bolded.

To better understand the specificity of the phage nuclear import machinery, we studied the ability of PhiPA3 and PhiKZ, two closely related *Pseudomonas* phages (4, 32, 33). to import each other’s proteins. We expressed five previously identified PhiPA3 nuclear proteins (13) with a C-terminal sfGFP tag (RecA, gp108, gp200, gp78, and gp257) in *Pseudomonas aeruginosa* and infected the cells with either PhiPA3 or PhiKZ [sfGFP alone does not localize to the phage nucleus of either phage (10)]. As expected, all five PhiPA3 proteins localized within the PhiPA3 nucleus (Fig. 1*A*). When cells were infected with PhiKZ, PhiPA3 RecA was imported into the PhiKZ nucleus, while the other four PhiPA3 proteins were excluded from the PhiKZ nucleus (Fig. 1*A*), demonstrating that PhiKZ does not import the majority of PhiPA3 nuclear proteins.

Three of the five PhiPA3 proteins have a homolog in PhiKZ: PhiKZ RecA, gp104, and gp171. As expected, these three PhiKZ proteins localize to the PhiKZ nucleus (Fig. 1*B*). PhiKZ RecA was also imported into the PhiPA3 nucleus. However, PhiKZ gp104 and gp171 were excluded from the PhiPA3 nucleus (Fig. 1*B*), demonstrating that the PhiPA3 nucleus excludes the majority of PhiKZ nuclear proteins as well. These data show that these phages primarily import their cognate proteins and exclude the proteins of other phages from their nucleus.

PhiKZ and PhiPA3 RecA share the highest sequence identity of the proteins tested (~80% amino acid sequence identity) (*SI Appendix*, Fig. S1*D*), but the other homolog pairs also share considerable amino acid sequence identity. PhiKZ gp104 and PhiPA3 gp108 share ~65%, while PhiKZ gp171 and PhiPA3 gp200 share ~54% (Fig. 1*G* and *SI Appendix*, Fig. S1*C*). The fact that these latter proteins only localize to their cognate nucleus demonstrates that these phages can discriminate between closely related homologs with significant sequence identity, suggesting the existence of phage-specific import recognition signals.

We investigated this hypothesis with PhiKZ gp104, a 162-amino acid protein of unknown function belonging to the conserved macrodomain family (34). We first expressed truncated versions of sfGFP-tagged PhiKZ gp104 in *P. aeruginosa* and infected with PhiKZ (Fig. 1 *C* and *D*). While full-length PhiKZ gp104 was imported (Fig. 1*B*), none of the truncations localized to the phage nucleus, suggesting that multiple distant sequences are required for import or that the phage recognizes the surface of a properly folded protein.

Next, we generated chimeras of PhiKZ gp104 and its homolog PhiPA3 gp108 and tested for localization to the PhiKZ nucleus to identify a region conferring import specificity (Fig. 1 *D* and *F*). PhiKZ gp104 and PhiPA3 gp108 have nearly identical N termini (amino acids 1 to 65) and are most divergent in the middle and C-terminal regions (Fig. 1 *D* and *G*). Since PhiPA3 gp108 does not localize to the PhiKZ nucleus (Fig. 1*A*) but shares high sequence and structural homology to PhiKZ gp104 (Fig. 1*G* and *SI Appendix*, Fig. S1*A*), this approach allowed us to screen for sequence-specific localization effects while still maintaining the full sequence length and general structure of the protein. As expected, fusing the N-terminal 65 amino acids of PhiKZ gp104 to the C-terminal 95 amino acids of PhiPA3 gp108 maintained the cytoplasmic localization of the chimeric PhiPA3 gp108 during PhiKZ infection (Fig. 1*F*). However, swapping the C-terminal ~2/3rds (amino acids 66 to 162) or the middle ~1/3 (amino acids 66 to 115) of PhiKZ gp104 into the homologous region of PhiPA3 gp108 caused the chimera to localize to the nucleus during PhiKZ infection (Fig. 1*F*). Shortening the region of PhiKZ gp104 swapped into PhiPA3 gp108 revealed three more chimeras that localize to the PhiKZ nucleus: chimeras containing PhiKZ gp104 amino acids 66 to 95, 77 to 100, or 77 to 95 (Fig. 1*F*). In comparison, three chimeras with shorter sequences from PhiKZ gp104 did not localize to the PhiKZ nucleus (Fig. 1*F*). These data indicate that at least part of the region between amino acids 77 and 95 is essential for PhiKZ gp104 localization to the phage nucleus, but the region between amino acids 79 to 88 is either insufficiently short or unnecessary. The region between amino acids 77 and 95 is ~47% identical between the homologs, demonstrating that the phage import machinery discriminates between only a small number (≤10) of nonconserved amino acids (*SI Appendix*, Fig. S1*B*).

Mapping the nonconserved amino acids in this region to the AlphaFold-predicted structure (35) of PhiKZ gp104 shows that they are part of an exposed surface of the protein that we call the recognition region (Fig. 1*E*). Aligning the sequence of this region with other known PhiKZ nuclear proteins including RecA, RNA polymerases, and gp171 did not reveal a conserved motif (36). These results indicate that protein trafficking into the phage nucleus likely involves a recognition surface rather than an N- or C-terminal signal sequence as found in many other import systems (18, 20, 22, 25, 27). This is consistent with previous results that two surface-exposed amino acids which are distant in the primary sequence are essential for the localization of GFPmut1 to the PhiKZ nucleus (10).

One sfGFP-tagged chimera containing the first 112 amino acids of PhiPA3 gp108 with the C-terminal segment of PhiKZ gp104 (amino acids 113 to 162) produced an unexpected phenotype. During PhiKZ infection, this protein accumulates in a single punctum at the periphery of the phage nucleus but fails to enter (Fig. 1*F*). This suggests there could be two major steps in the import process, nuclear targeting and translocation across the nuclear shell, and that the chimera may be a stalled transport intermediate. The presence of a single focus instead of multiple foci spread around the nuclear shell additionally suggests that the nucleus may have a single site of protein import.

### Mutations in the Conserved Shell-Associated Protein PicA Alter Selective Protein Trafficking into the Phage Nucleus.

**Fig. 2.**
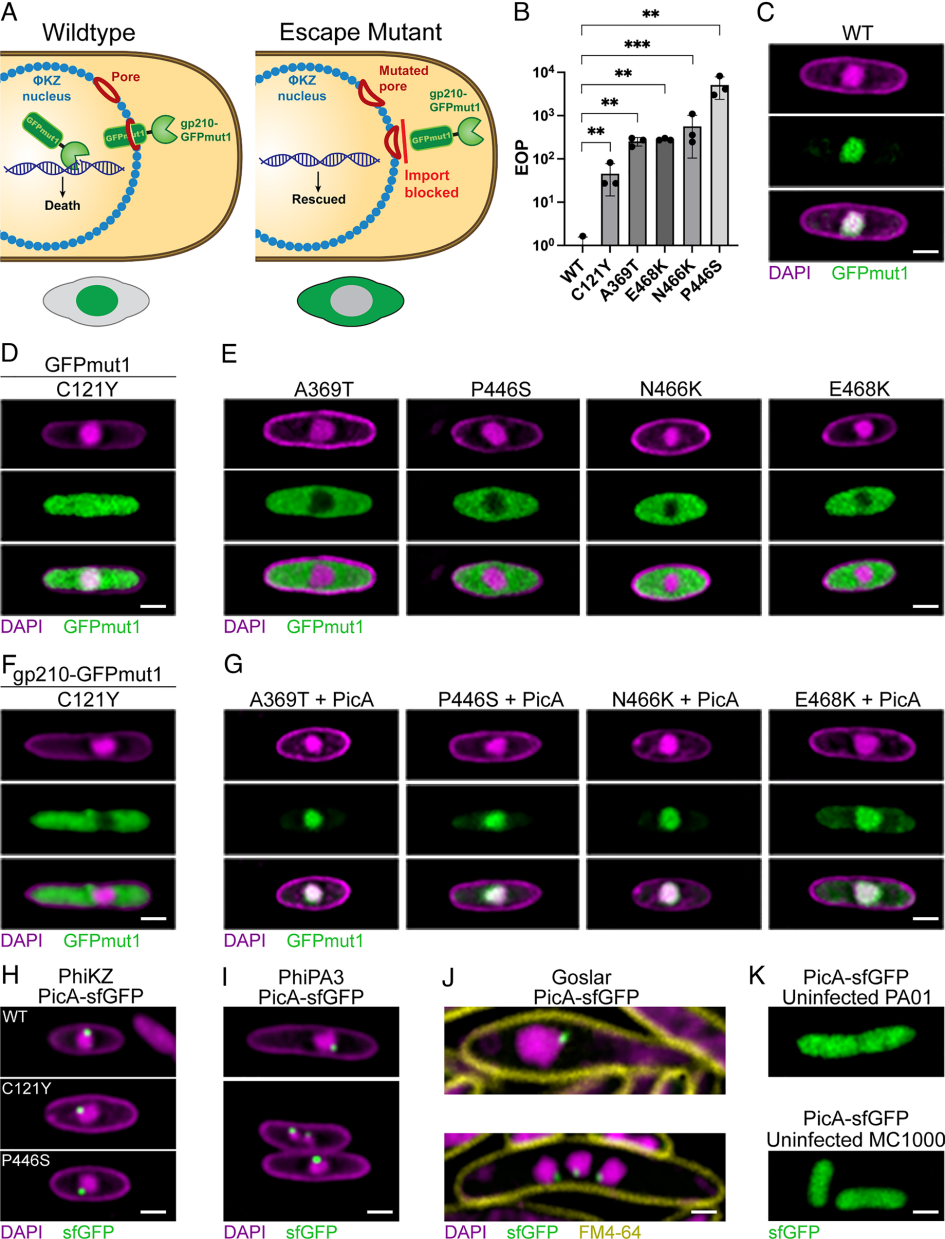
The nuclear shell–associated protein PicA plays a key role in selective protein trafficking into the phage nucleus. (*A*) Schematic illustrating the genetic selection performed with the toxic fusion protein gp210-GFPmut1 targeted to the PhiKZ nucleus with the expected GFPmut1 localization phenotype of the wild-type and mutant PhiKZ depicted below. (*B*) Mean efficiency of plating of mutant PhiKZ relative to the wild type calculated by spot titer on lawns of *P. aeruginosa* expressing gp210-GFPmut1. Error bars indicate SD. **P < 0.01, ***P < 0.001. Significance was determined by the paired *t* test. (*C*) Microscopy image of *P. aeruginosa* expressing GFPmut1 infected with wild-type PhiKZ. All *P. aeruginosa* phage infections imaged at 30 to 45 mpi. (*D*) *P. aeruginosa* expressing GFPmut1 infected with PicA C121Y mutant PhiKZ. (*E*) *P. aeruginosa* expressing GFPmut1 infected with PhiKZ containing the indicated mutation in PicA. (*F*) *P. aeruginosa* expressing gp210-GFPmut1 infected with PicA C121Y mutant PhiKZ. (*G*) *P. aeruginosa* expressing both wild-type PicA and GFPmut1 infected with the indicated PicA mutant PhiKZ. (*H*) *P. aeruginosa* expressing sfGFP-tagged wild-type or mutant PicA infected with wild-type PhiKZ. (*I*) *P. aeruginosa* expressing sfGFP-tagged wild-type PicA from phage PhiPA3 infected with PhiPA3. The *Upper* panel shows a cell with a single nucleus, while the *Lower* panel depicts a cell with multiple phage nuclei and a single PicA punctum on each nucleus. (*J*) *E. coli* strain MC1000 expressing sfGFP-tagged Goslar wild-type PicA infected with Goslar. The *Upper* panel shows a cell with a single nucleus, while the *Lower* panel depicts a cell with multiple phage nuclei and a single PicA punctum on each nucleus. Images were taken at 75 to 90 mpi. (*K*) PhiKZ PicA-sfGFP expressed in an uninfected *P. aeruginosa* PA01 K2733 cell (*Left*) or Goslar PicA-sfGFP expressed in an uninfected *E. coli* MC1000 cell (*Right*). (Scale bars, 1 µm.) All cells are representative of the population. Larger fields of view appear in *SI Appendix*.

To determine the identity of the factors that facilitate protein trafficking into the phage nucleus, we designed a genetic selection to isolate mutant phage that no longer import a toxic protein, a similar method as that which was used to initially identify many of the protein secretion genes in *Escherichia coli* (37). We expressed a fusion between GFPmut1 and the PhiPA3 endonuclease gp210 and then infected with PhiKZ (Fig. 2*A*). GFPmut1 can be used to drive PhiKZ nuclear import of proteins that are normally excluded and remain in the host cytoplasm (10, 15, 16). PhiPA3 gp210 is normally excluded from the PhiKZ phage nucleus, but gp210-GFPmut1 is imported, where it becomes toxic by cleaving the PhiKZ genome at a single recognition site and preventing replication (15, 16). We therefore predicted that through selection for phage that can replicate in the presence of gp210-GFPmut1, we could isolate escape mutants that have altered the ability to traffic proteins into the phage nucleus (Fig. 2*A*).

Strains expressing gp210-GFPmut1 exhibit a ~10,000-fold reduction in PhiKZ plaquing, and mutants that escape this selection can be isolated (16). Here, we isolated PhiKZ gp210-GFPmut1 resistant mutants and found they had mutations in one of two genes with approximately equal frequency (Table 1). The first is the virion RNA polymerase subunit gp178 gene, which contains the previously identified gp210 nuclease target site (16). Mutations at this site are expected to reduce or eliminate gp210-mediated cleavage, rendering the mutant resistant to gp210. The second mutated gene encodes a protein of unknown function, gp69. Five independent mutant phages with a single mutation in gp69 were isolated and each of these mutants showed impaired protein localization to the phage nucleus, leading us to name this protein PicA (Fig. 2 *D*–*F*). These mutants show a ~50- to ~5,000-fold rescue in titer compared to the wild type when plaquing on cells expressing gp210-GFPmut1 (Fig. 2*B*). Live cell imaging of infected cells showed that four of the five isolated mutants (A369T, P446S, N466K, and E468K) exclude GFPmut1 (Fig. 2*E*) and gp210-GFPmut1 from the PhiKZ nucleus (*SI Appendix*, Fig. S2). The fifth mutant (C121Y) does not fully exclude GFPmut1 from the nucleus (Fig. 2*D*), but does exclude the gp210-GFPmut1 fusion protein from the nucleus (Fig. 2*F*). Infections with all of these mutant phages appeared to progress normally and still concentrate sfGFP-tagged PhiKZ nuclear proteins (*SI Appendix*, Fig. S3), suggesting that import of endogenous phage proteins is not strongly affected by these point mutations. Furthermore, expressing wild-type PicA in conjunction with GFPmut1 and infecting with mutant phage restores the nuclear localization of GFPmut1, indicating that the effect is specific to the mutations in PicA and that these mutant phenotypes are recessive (Fig. 2*G*). We attempted to perform a similar selection with a PhiKZ nuclear protein; however, selecting mutants with PhiKZ gp104 fused to PhiPA3 gp210 only produced escape mutants that had altered the nuclease target site in gp178 (Table 1). This suggests that complete loss of nuclear import of an endogenous phage protein, gp104, was more detrimental to phage fitness than mutating the nuclease target site.

**Table 1.** PhiKZ mutants isolated in this study. Phenotypes in bold exhibit a defect in protein import.

Method of isolation	Gene product mutated	DNA mutation	Amino acid mutation	Frequency	Phenotype
gp210-GFPmut1 selection	gp178	a3215c	D1072A	7/32	Resistant to nuclease activity
	gp178	a3215g	D1072G	7/32	Resistant to nuclease activity
	gp178	g3214t	D1072Y	4/32	Resistant to nuclease activity
	PicA (gp69)	g362a	C121Y	1/32	**Excludes gp210-GFPmut1 from the phage nucleus, but not GFPmut1**
	PicA (gp69)	g1105a	A369T	1/32	**Excludes GFPmut1 from the phage nucleus**
	PicA (gp69)	c1336t	P446S	8/32	**Excludes GFPmut1 from the phage nucleus**
	PicA (gp69)	t1398g	N466K	3/32	**Excludes GFPmut1 from the phage nucleus**
	PicA (gp69)	g1402a	E468K	1/32	**Excludes GFPmut1 from the phage nucleus**
gp210-gp104 selection	gp178	g3213t	D1072Y	5/10	Resistant to nuclease activity
	gp178	a3214g	D1072G	4/10	Resistant to nuclease activity
	gp178	a3212g	D1071G	1/10	Resistant to nuclease activity
Cas13a PicA g3	PicA (gp69)	t222g	R74R	1/7	No phenotype observed
	PicA (gp69)	g221a	R74H	5/7	No phenotype observed
	PicA (gp69)	c224g	A75G	1/7	No phenotype observed
Cas13a PicA g4	PicA (gp69)	t1077a	H359Q	4/17	No phenotype observed
	PicA (gp69)	g1081c	G361C	3/17	No phenotype observed
	PicA (gp69)	t1092g	D364E	1/17	No phenotype observed
	PicA (gp69)	t1095c	H365H	5/17	No phenotype observed
	PicA (gp69)	t1095a	H365Q	1/17	**Excludes GFPmut1 from the phage nucleus**
	PicA (gp69)	a1097c	H366P	2/17	No phenotype observed
	PicA (gp69)	a1099c	T367P	1/17	**Delayed nuclear localization**
Cas13a PicA g6	PicA (gp69)	c889a	Q297K	10/10	**Altered gp104 nuclear localization**

The *picA* gene is part of the Chimalliviridae core genome and is conserved in all known phages that encode the nuclear shell protein ChmA (4) (*SI Appendix*, Fig. S4). Furthermore, the PicA homolog from phage PhiPA3 (gp63) was recently identified as nuclear shell-associated and part of the ChmA interaction network (13). Live cell imaging of infected cells expressing sfGFP-tagged PicA showed that PicA from diverse phages, including PhiKZ, PhiPA3, and *E. coli* phage Goslar (3, 38), localizes to the nuclear periphery (Fig. 2 *H*–*J* and *SI Appendix*, Fig. S5). For example, PhiKZ PicA-sfGFP localized into a single punctum at the nuclear periphery during PhiKZ infection (Fig. 2*H*) but is diffuse in uninfected cells (Fig. 2*K*). This localization was not affected by mutations (C121Y and P446S) in PicA that alter GFPmut1 import (Fig. 2*H*). PhiPA3 PicA (Fig. 2*I*) and Goslar PicA (gp174) (Fig. 2*J*) also localized in a single punctum at the nuclear periphery during infection of their respective hosts, *P. aeruginosa* and *E. coli*. When infected cells displayed more than one nucleus during infection, a single PicA-sfGFP punctum was seen at the periphery of each nucleus (Fig. 2 *I* and *J*), suggesting that this phenotype is biologically relevant and not due to nonspecific aggregation of the sfGFP-tagged protein. Together, these results suggest that PicA is a conserved protein that interacts with the nuclear shell for the purpose of trafficking proteins across the ChmA lattice.

**Fig. 3.**
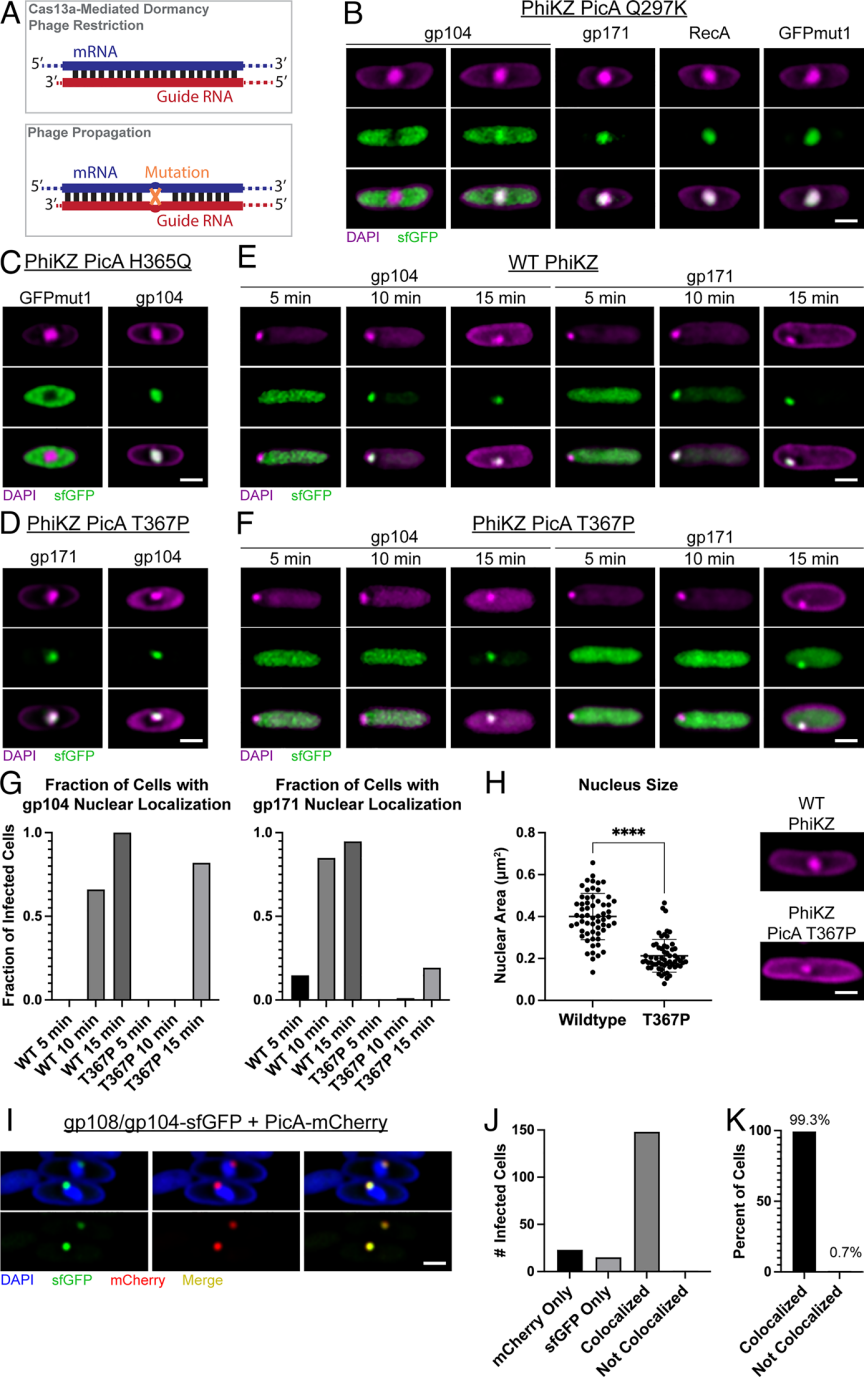
PicA traffics phage proteins into the phage nucleus. (*A*) Schematic of the mechanism of mutant isolation with catalytically active Cas13a. Anti-sense guide RNAs pair with mRNA of expressed genes and activate Cas13a, which leads to cell dormancy and phage restriction. Mutant phages that exist in the population escape targeting and become the dominant phage in the population. (*B*) *P. aeruginosa* cells expressing the indicated sfGFP-tagged PhiKZ protein infected with PhiKZ PicA Q297K mutant. Images were taken 30 to 45 mpi. Two examples for sfGFP-tagged PhiKZ gp104 show it is either partially or fully excluded by PhiKZ PicA Q297K. (*C*) *P. aeruginosa* cells expressing GFPmut1 (*Left*) or sfGFP-tagged PhiKZ gp104 (*Right*) infected with PhiKZ PicA H365Q mutant imaged at 30 mpi. (*D*) *P. aeruginosa* cells expressing the indicated construct infected with wild-type or PicA T367P mutant PhiKZ imaged at 30 mpi. (*E*) *P. aeruginosa* cells expressing sfGFP-tagged PhiKZ gp104 (*Left*) or sfGFP-tagged gp171 (*Right*) infected with wild-type PhiKZ. Images were taken after 5, 10, or 15 min of incubation with phage. (*F*) Same as in *E* except cells are infected with PicA T367P mutant PhiKZ. (*G*) Quantification of the fraction of infected cells that displayed a nuclear localization of the specified protein at the indicated time point as shown in (*E*) and (*F*). Nuclear localization during infection was called if the maximum GFP signal colocalized with the DAPI-stained phage genome. Localization was called for the total number of infected cells imaged for each condition (n = 68 to 159 per condition). (*H*) DAPI-stained wild-type *P. aeruginosa* cells infected with wild-type or PicA T367P mutant PhiKZ at 30 mpi with quantification of nuclear area for cells infected with wild-type or PicA T367P mutant PhiKZ imaged. The line indicates mean and error bars indicate SD. N = 60 per condition. ****P < 0.0001 by the Mann–Whitney test. (*I*) *P. aeruginosa* cells coexpressing a stalled PhiPA3 gp108/PhiKZ gp104 chimera (PhiPA3gp108[PhiKZgp104(113-162)]) tagged with sfGFP and PicA tagged with mCherry infected with wild-type PhiKZ. DAPI stain is shown in blue, sfGFP in green, and mCherry in red. Colocalization of mCherry and sfGFP can be seen in yellow. Lower panels depict only the fluorescent protein. (Scale bars, 1 µm.) All cells are representative of the population. Larger fields of view appear in *SI Appendix*. (*J*) Number of infected cells coexpressing both sfGFP-tagged chimera and PicA-mCherry that have fluorescence in at least one channel above threshold displaying the indicated phenotype. N = 187 cells (mCherry only = 23, sfGFP only = 15, mCherry/sfGFP with colocalization = 148, mCherry/sfGFP without colocalization = 1). (*K*) Percent of infected cells with both mCherry and sfGFP fluorescence above threshold that display either overlapping (colocalized, 99.3%) or nonoverlapping (distinct, 0.7%) fluorescent puncta at the nuclear periphery. N = 149 cells. See *SI Appendix*, Fig. S5 for more detail.

To gain deeper understanding of the key structural or sequence elements required for PicA to modulate protein import, we generated a series of *picA* mutants with LbuCas13a (*Leptotrichia buccalis* Cas13a, henceforth Cas13a) using guides targeting specific regions of PhiKZ PicA (39). Cas13a uses a guide RNA to recognize specific phage-encoded RNAs, which activates its nonspecific RNA nucleolytic activity. This leads to degradation of both phage and host cellular RNA, cell dormancy, and phage restriction (40) (Fig. 3*A*). Cas13a produces a strong selection against the wild-type gene so only phage with mutations in the targeted region can replicate (39, 41). RNA-targeting Cas systems are particularly well-suited for counterselection of chimalliviruses, as their DNA is protected within the phage nucleus, but their mRNA is not (6, 12, 39, 41, 42). Regions of the *picA* gene to be targeted with guides were chosen based upon the AlphaFold2 prediction of the PicA structure (*SI Appendix*, Fig. S6). Sequences proximal to the previously generated mutants, as well as sequences corresponding to additional faces of the protein were targeted with guide RNAs containing 31-nucleotide spacer sequences. We transformed plasmids expressing both Cas13a and single guides against *picA* into *P. aeruginosa* and infected with wild-type PhiKZ. In doing so, we selected spontaneous phage mutants that escape guide recognition and subsequently screened them for protein import defects. Of the seven guides tested, three (guide 3, 4, and 6) produced escape mutants. Among the 34 PhiKZ mutants we isolated, we observed 11 unique mutations in the *picA* gene (Table 1). Two were silent mutations, while the other nine resulted in single amino acid substitutions. Six of these nine mutants showed no change in their ability to import nuclear proteins. One of the remaining three mutants (H365Q) showed a defect only in GFPmut1 localization (Fig. 3*C*), demonstrating that we can recapitulate the previously identified phenotype (Fig. 2*E*) with this selection method.

The remaining two mutants (Q297K and T367P) showed a defect in the nuclear localization of one or more sfGFP-tagged phage proteins, implicating PicA in the endogenous protein trafficking pathway. PicA Q297K mutant PhiKZ failed to fully concentrate sfGFP-tagged PhiKZ gp104 in the phage nucleus after 30 min postinfection (mpi), while nuclear localization of PhiKZ gp171, PhiKZ RecA, or GFPmut1 were unaffected (Fig. 3*B*). PicA T367P mutant PhiKZ concentrated both PhiKZ gp104 and PhiKZ gp171 in the nucleus by 30 mpi (Fig. 3*D*) but showed a temporal delay in protein localization and produced smaller nuclei (Fig. 3 *D*–*G*). At 5 mpi, the phage genome can be seen as a DAPI-stained punctum at the pole of the cell in both wild-type and PicA T367P mutant PhiKZ infections. At this time point, exogenously expressed sfGFP-tagged PhiKZ gp104 and gp171 are diffuse in the cytoplasm (Fig. 3 *E*–*G*). By 10 mpi, sfGFP-tagged PhiKZ gp104 and gp171 are predominantly localized to the wild-type phage nucleus, while the fluorescence signal from both proteins is still diffuse in the cytoplasm during infection by the mutant. PhiKZ gp104-sfGFP and gp171-sfGFP do not concentrate in the mutant nucleus until 15 mpi or later (Fig. 3 *E*–*G*). However, at 30 mpi PicA T367P mutant nuclei are significantly smaller than wild-type nuclei, indicating a delay in nuclear development and that protein import is likely required for nucleus growth (Fig. 3*H*). We were unable to accurately measure the kinetics of PhiKZ RecA-sfGFP import in early infection due to its tendency to form foci in the cytoplasm prior to import. Neither the T367P mutation nor the Q297K mutation alters the localization of PicA (*SI Appendix*, Fig. S7). Together, these two mutations (Q297K and T367P) implicate PhiKZ PicA in the trafficking of endogenous phage proteins into the phage nucleus.

### The PicA Import Machinery Colocalizes with a Transport Intermediate.

Since mutations in PicA alter selective protein trafficking, we sought to determine whether PicA associates with imported proteins during phage infection. One sfGFP-tagged chimera of PhiPA3 gp108 and PhiKZ gp104 (PhiPA3gp108-[PhiKZ gp104(113-162)]-sfGFP) is diffuse in the cytoplasm of uninfected cells (*SI Appendix*, Fig. S8) but accumulates in a single punctum on the phage nuclear shell during infection, likely representing a stalled transport intermediate (Fig. 1*F*). Notably, fluorescently tagged PicA also localizes in a single punctum on the phage nucleus surface (Fig. 2 *H*–*J*). If PicA is part of the import machinery, we would expect it to colocalize with the putative transport intermediate. Therefore, we infected cells coexpressing the sfGFP-tagged stalled chimera and mCherry-tagged PicA with PhiKZ (Fig. 3*I* and *SI Appendix*, Fig. S9). Of the 187 infected cells observed, 149 contained puncta of both PicA-mCherry and stalled chimera-sfGFP, while the other 38 only contained a detectable punctum of one fluorescently tagged protein (Fig. 3*J*). Strikingly, in cells where both fluorescent fusions were detected, PicA-mCherry and stalled chimera-sfGFP colocalize on the nuclear periphery in 99.3% of infections (n = 149) (Fig. 3*K* and *SI Appendix*, Fig. S9). Since the PhiKZ PicA Q297K mutant shows reduced trafficking of gp104 into the phage nucleus, we also tested whether this mutant shows a decrease in accumulation of this stalled gp104 chimera. This mutant showed a ~sixfold reduction in the number of nuclei with the chimera accumulated on the nuclear periphery (*SI Appendix*, Fig. S10). Together, this provides evidence that PicA forms part of the import machinery through which imported proteins pass and supports a mechanism in which proteins are targeted to PicA for translocation across the phage nuclear shell.

### PicA Is Essential for Phage Nucleus Maturation and Phage Replication.

**Fig. 4.**
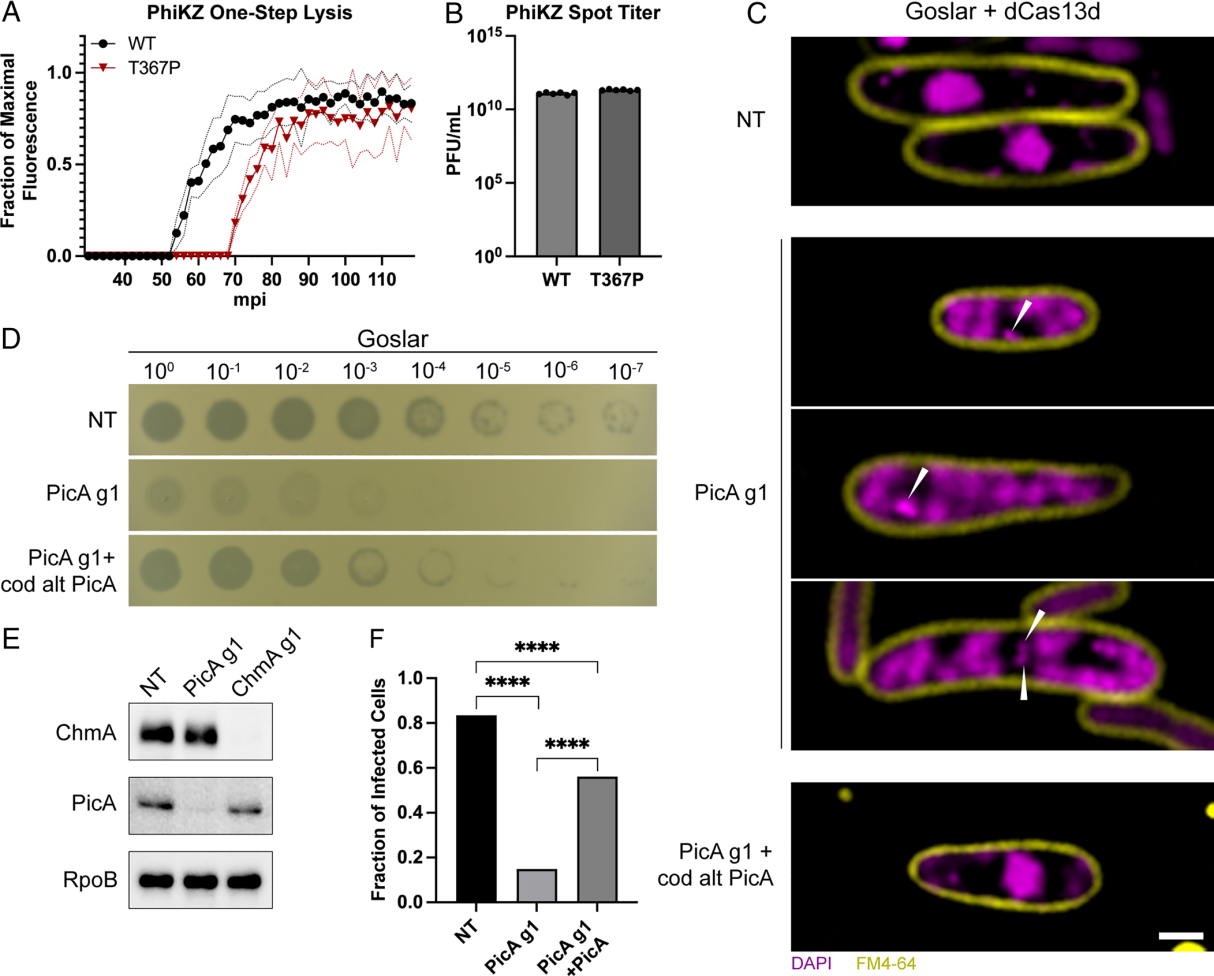
PicA is essential for replication and phage nucleus maturation. (*A*) One-step lysis curves of *P. aeruginosa* infected with wild-type or PicA T367P mutant PhiKZ. Phage-induced lysis was detected by Sytox green. Data shown as fraction of maximal fluorescence. The solid line depicts the mean across n = 12 replicates. Dotted lines show mean ± SD. (*B*) PFU/mL of wild-type and PicA T367P mutant PhiKZ as determined by spot titer on lawns of *P. aeruginosa*. N = 5 per condition. (*C*) *E. coli* expressing the indicated constructs (NT = nontargeting) and infected with wild-type Goslar. DNA is visualized with DAPI (purple), and cell membranes are visualized with FM4-64 (yellow). Location of the phage genome in arrested infections is located at the point of the white triangles. Images were taken at 75 to 90 mpi. (Scale bar, 1 µm.) All cells are representative of the population. Larger fields of view appear in *SI Appendix*. (*D*) Spot titer of wild-type phage Goslar on lawns of *E. coli* expressing dCas13d with the guides and protein indicated on the left. PicA exogenous expression constructs are codon altered to be insensitive to dCas13d PicA g1 targeting. Dilution of phage from high titer starting lysate (>10^10^ PFU/mL) is indicated above. The experiment was performed in triplicate, and a representative image is shown. (*E*) Western blot of Goslar-infected *E. coli* cells expressing dCas13d with the indicated guide RNA at 90 mpi. The blot was probed sequentially with anti-PicA and anti-ChmA polyclonal antibodies, stripped, and reprobed with anti-RpoB as a loading control. (*F*) Percent of infected cells imaged in (*B*) that contained a phage nucleus > 0.5 µm (n = 121 to 182 infected cells per condition). ****P < 0.0001 as determined by Fisher’s exact test.

PhiKZ PicA T367P mutant phage have a delay in importing PhiKZ proteins and a delay in phage nucleus growth. To determine whether these delays affect the timing of the PhiKZ lytic cycle, we performed a single-step lysis curve in which we monitored cell lysis with SYTOX Green, a cell impermeable fluorescent DNA dye, as a reporter for the completion of the phage lytic cycle (43). Cells infected with wild-type PhiKZ lysed at an average of 62 mpi (n = 12) as determined by the time at which 50% maximal fluorescence was reached, whereas lysis of cells infected with PhiKZ PicA T367P was delayed until an average of 75 mpi (n = 12) (Fig. 4*A*). Despite this delay in lysis time, the mutant phage displayed no decrease in titer when plated on wild-type *P. aeruginosa* (Fig. 4*B*). The delay in completing the lytic cycle together with the delay in phage nucleus growth suggests that the protein trafficking functions provided by PicA are important for efficient PhiKZ replication and raise the question of whether this highly conserved core gene plays an essential role that is conserved in other phages.

Selection against PhiKZ PicA with catalytically active Cas13a only produced escape mutants with single-nucleotide missense mutations (Table 1), suggesting that the gene encoding PicA is essential. To determine how loss of PicA affects *E. coli* phage Goslar replication, we used the recently developed CRISPRi-ART approach, which utilizes a catalytically deactivated *Ruminococcus flavefaciens* Cas13d (dCas13d) (44, 45) to selectively knock down Goslar PicA during infection of *E. coli.* We chose to focus on Goslar since we found that the dCas13d repression system was less effective when used in *P. aeruginosa* against PhiKZ.

Spot titers of Goslar on lawns of *E. coli* cells expressing dCas13d and a guide targeting the region surrounding the start codon of PicA (PicA g1) required a ~10,000-fold higher phage titer for clearing than strains expressing a nontargeting guide (Fig. 4*D*). The clearings in PicA g1 lawns are opaque and do not form individual plaques, making exact efficiency of plating quantification difficult. However, the large order of magnitude decrease in phage clearing was readily apparent and consistent across replicates. Complementation of the PicA knockdown with a plasmid-expressed codon-altered *picA* gene that is not targeted by the guide restored phage titer, showing that the effect of the knockdown is specific to PicA (Fig. 4*D*).

We used fluorescence microscopy to understand at which stage infections were blocked in the absence of PicA expression. Individual host cells expressing dCas13d PicA g1 displayed hallmarks of early infection, including cell swelling and host genome degradation, but were arrested at an early stage of phage replication (Fig. 4*C*). Even by 75 to 90 mpi, the phage nucleus failed to mature and the phage genome appeared as a small DAPI-stained focus in the bacterial cell (Fig. 4*C*, white arrows). Frequently, multiple phage genomes can be seen in cells with arrested infections (Fig. 4*C*). A similar phenotype has previously been observed in infections where the essential nuclear shell protein ChmA is selectively knocked down (45). Western blots of infected cells confirmed that PicA expression was strongly inhibited by PicA g1 expression, but not by the nontargeting guide nor ChmA g1 controls (Fig. 4*E*). Furthermore, ChmA was expressed at approximately wild-type levels during PicA knockdown indicating that the arrested infection is still transcriptionally active (Fig. 4*E*). Complementation of the knockdown with a codon-altered untargetable PicA restored nucleus formation (Fig. 4 *C* and *F*). Quantification of >100 infected cells per condition shows that a maturing phage nucleus greater than 0.5 micron in diameter formeds in the majority (84%) of infected cells expressing the nontargeting guide (Fig. 4*F*). However, infected cells expressing the PicA targeting guide only exhibited a maturing phage nucleus 15% of the time. Expressing the nontargetable PicA along with PicA g1 increased the number of maturing phage nuclei present to 56% (Fig. 4*F*). These complementation results are in agreement with spot titer results (Fig. 4*D*), indicating that this phenotype is specific and not due to off-target or polar effects on cotranscribed genes. However, sfGFP-tagged version of PicA failed to rescue, suggesting the C terminus is important for function (*SI Appendix*, Fig. S11). Together, these data indicate that PicA plays an essential role early in nucleus-based phage replication.

## Discussion

### A Proposed Mechanism for Protein Trafficking into the Phage Nucleus.

**Fig. 5.**
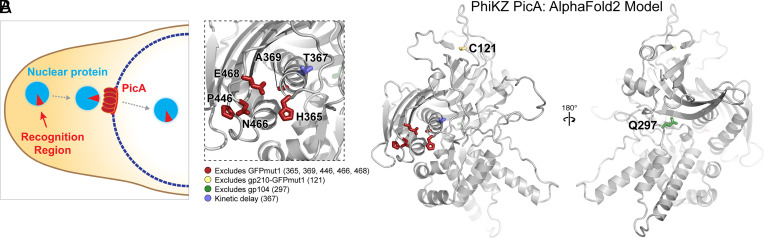
Model for the mechanism of selective protein trafficking into the phage nucleus. (*A*) Mechanistic models for selective protein trafficking into the phage nucleus. (*B*) AlphaFold2 predicted structure of PhiKZ PicA with the location of amino acids which display protein import defects when mutated. The *Inset* to the left shows the clustering of mutations that prevent GFPmut1 import (A369, P446, N466, and E468), as well as the mutation that alters the timing of protein import (T367). The structure to the right shows mutations that block only gp210-GFPmut1 import (C121) or alter gp104 import (Q297). Mutations shown in the box on the left are in the region of PicA that is the most confidently predicted by AlphaFold2 (*SI Appendix*, Fig. S5).

In order to achieve selective protein trafficking into the phage nucleus, the proteins destined for import must be recognized in the cytoplasm and transported across the ChmA shell. We found that the import pathways of PhiPA3 and PhiKZ are highly effective at discriminating between each other’s proteins. We have identified a specific recognition region of an endogenous PhiKZ nuclear protein, gp104, required for transporting it into the nucleus and a conserved shell-associated protein, PicA, that comprises part of the protein import machinery. One of the chimeras generated between PhiKZ gp104 and its homolog PhiPA3 gp108 (PhiPA3gp108-[PhiKZ gp104(113-162)]-sfGFP) revealed a stalled transport intermediate that was targeted to the PhiKZ import machinery, but failed to be transported. The stalled intermediate colocalized with fluorescently tagged PicA foci on the nuclear periphery, suggesting a two-step import model in which proteins are first targeted to PicA and then licensed to be translocated across the nuclear shell by the import machinery (Fig. 5*A*). This process is dependent upon amino acids predicted to be on the surface of the imported protein (Fig. 5*A*), consistent with prior results showing that single amino acid substitutions on the surface of GFPmut1 affect import into the PhiKZ nucleus and suggesting that proteins are recognized in their folded state. Stalled transport intermediates have been studied extensively for the Sec translocase where they block protein translocation (37). However, our stalled transport intermediate appears not to block the import of essential proteins. Many explanations for this exist including that the intermediate is trapped upstream of a point where import would be blocked, that the mechanism of protein trafficking can accommodate multiple trafficked proteins at once including stalled proteins, or that a block indeed occurs, but occurs after a sufficient number of essential proteins have been imported. When PicA expression is prevented using CRISPRi-ART, phage nucleus development is halted at an early stage of infection, suggesting that the protein import pathway is essential for the phage nucleus-based life cycle. Our PicA mutants directly implicate PicA in the trafficking of both endogenous (gp104 and gp171) and nonendogenous (GFPmut1 and gp210-GFPmut1) proteins into the phage nucleus (Figs. 2 and 3). The fact that mutants with a complete loss of protein import would be nonviable and that PicA is essential, conserved in all known nucleus-forming phages, and associated with the phage nuclear shell, suggests it is a general factor for selective protein trafficking into phage nucleus. We cannot rule out that PicA performs other essential functions in nucleus-based phage replication, particularly early in infection, nor that other factors are involved in nuclear trafficking or that auxiliary pathways of import exist for other proteins.

The PIC pathway is different from other protein translocation systems, such as bacterial TAT and Sec pathways, that depend upon a simple signal sequence at the N or C terminus of cargo proteins (27, 30, 31). These cellular systems have evolved to be universal and interchangeable, so one common targeting sequence can be recognized in many divergent species of bacteria, enabling new genes acquired via horizontal gene transfer to be properly localized. In contrast, chimalliviruses face selection pressures from both other chimalliviruses and the host, likely shaping the evolution of distinct import selectivities. The PIC pathway, similar to proposed mechanisms for Type II Secretion systems (46), relies on multiple amino acids on the surfaces of both the folded cargo proteins and PicA for import. This provides high substrate selectivity that can be maintained and evolved such that competing phages like PhiPA3 and PhiKZ can coexist in the same virocell without importing each other’s proteins (Fig. 1*A*) (15). We have recently shown that PhiPA3 and PhiKZ frequently coinfect and compete with one another (15, 16). Indeed, importing PhiPA3’s homing endonuclease gp210 into PhiKZ’s nucleus is fatal for PhiKZ (Fig. 2) (15, 16). Thus, toxic proteins produced by competing chimalliviruses provide a strong selective pressure to evolve import systems with distinct selectivities (Fig. 2). The selectivity of the PIC pathway also protects the phage genomes from host defense proteins, preventing import of DNA-targeting CRISPR-Cas and restriction enzymes (6, 12). Notably, mutations in host defense factors that overcome this protection by targeting to one phage’s nucleus would not defend against other nucleus-forming phages. Thus, the overall mechanism described here requiring two steps (targeting and licensing) and multiple surfaces of cargo and transporter ensures import specificity, protecting these phages from competing viruses and host defenses, and likely plays an important role in shaping the evolution of nucleus-forming phages.

AlphaFold2 (35) produces a predicted structure of PhiKZ PicA with regions of high confidence (Fig. 5*B* and *SI Appendix*, Fig. S6). Foldseek and DALI searches find no structural homologs (47, 48). Mapping the mutations generated in this study onto the predicted structure of PicA provides insights into the regions important for selective trafficking (Fig. 5*B*). Six mutations, A369T, P446S, N466K, E468K, H365Q, and T367P, all occur within ~13 Å of each other in structural space despite being distant from each other in the primary sequence. Two other mutations, Q297K and C121Y, occur on surfaces distant from each other and the other mutations isolated (Fig. 5*B*). While the T367P mutation imparts a general defect on protein trafficking, the rest of these mutations provide a deficit in trafficking only one or a small number of nuclear proteins (Fig. 5*B*). Together these mutations demonstrate that residues on various regions of PicA’s surface play critical and differing roles in cargo selection. Protein import into the phage nucleus is essential, remarkably selective (1, 4, 13), and able to discriminate between closely related proteins (Fig. 3), so it is not surprising that mutations in PicA with effects on specific substrates were identified. Since import of many proteins (RNAP, DNAP, etc.) is essential for replication, mutants that exclude these proteins or all proteins would be nonviable and therefore impossible to isolate. However, the fact that different mutations in PicA alter the trafficking of both endogenous and nonendogenous substrates, and that four substrates tested were affected by at least one mutation, supports that it is part of the general protein trafficking pathway.

The presence of a pore-like structure in the phage nuclear shell for macromolecular transport has been postulated since the discovery of the phage nucleus (1, 5), but pores large enough to accommodate protein trafficking have yet to be observed in tomograms of infected cells (1, 3, 4, 7, 10). One potential explanation for this is that only a limited number of protein trafficking complexes exist on the nuclear shell. Indeed, sfGFP-tagged PicA forms a single punctum on the nuclear periphery of three different phages, suggesting a higher-order oligomeric state. AlphaFold2 does not produce high-confidence oligomers of PicA, which likely suggests that other yet-to-be-identified proteins are required for the formation of the protein trafficking complex. The observation of a single PicA punctum that colocalizes with a stalled transport intermediate suggests that there may be only a single complex on the nuclear shell where protein trafficking occurs.

## Materials and Methods

### Bacterial Strains, Phage, and Growth Conditions.

*P. aeruginosa* PAO1 K2733 was used as the host for PhiKZ and PhiPA3. It was grown at 30 °C in LB, and 15 μg/ml gentamicin was used for plasmid selection. Phage stocks of PhiKZ and PhiPA3 were collected from plate lysates using phage buffer [10 mM Tris (pH 7.5), 10 mM MgSO_4_, 68 mM NaCl, and 1 mM CaCl_2_] and stored at 4 °C with titers of ~10^11^ PFU/mL. *E. coli* MC1000 was used as the host for Goslar. It was grown at 37 °C, and 30 μg/mL chloramphenicol and/or 100 μg/mL ampicillin were used for plasmid selection. Phage stocks of Goslar were collected from plate lysates using phage buffer and stored at 4 °C with titers of ~10^10^ PFU/mL.

### Plasmid Construction and Transformation.

Plasmids were synthesized and cloned by Genscript. The vector for all *P. aeruginosa* plasmids was pHERD-30 T. The vector for *E. coli* plasmids was either pDSW206 or p15a-CmR. Plasmid transformation was accomplished by 2 kV electroporation.

### Single-Cell Infection Assay.

Single-cell infections of *P. aeruginosa* and *E. coli* were visualized using fluorescence microscopy. *P. aeruginosa* was grown at 30 °C rotating up to an OD_600_ ~0.5 and mixed with PhiKZ or PhiPA3 lysate at a ratio of 1:100 lysate:culture. For protein expression, LB was supplemented with 15 μg/mL gentamicin and 0.1% arabinose. Infections were incubated rotating at 30 °C and then inoculated on 1% agarose pads containing 25% LB and 1 µg/mL DAPI and dried for 5 min at room temperature before a coverslip was applied. Alternatively, *E. coli* was grown at 37 °C to an OD_600_ 0.3 and inoculated on 1% agarose pads containing 25% LB with antibiotics if indicated and grown for 2 h at 37 °C. For protein expression, the agarose pad was supplemented with 0.2 mM IPTG (pDSW206) and/or 25 nM aTc (p15A-CmR). A volume of 10 μL of Goslar lysate was added and incubated at 37 °C. Cells were stained with 25 µg/mL DAPI and 3.75 ug/mL FM4-64 in 25% LB before imaging. Imaging was performed with a DeltaVision Elite Deconvolution microscope (Applied Precision, Issaquah, WA). Images were further processed by the aggressive deconvolution algorithm in the DeltaVision SoftWoRx 6.5.2 Image Analysis Program.

### Plaque Assay.

For Goslar, 9 mL of 0.35% LB top agar containing antibiotics and inducing agents were combined with 1 mL of overnight *E. coli* culture and poured onto LB plates containing the required antibiotics. Expression of dCas13d was induced with 200 nM aTc and expression of PicA was induced with 0.2 mM IPTG. A 10-fold dilution series of Goslar was prepared in LB and 2 µL were spotted for each dilution and incubated at 37 °C for 15 to 18 h.

For *P. aeruginosa*, liquid cultures were preinduced with 0.1% arabinose if indicated and grown to an OD_600_ ~0.5. Then, 200 μL of culture was mixed with 5 mL 0.35% LB top agar ± 0.1% arabinose and poured onto a plate with the appropriate antibiotic. A 10-fold dilution series of PhiKZ was prepared in phage buffer and 2 μL were spotted for each dilution and incubated at 30 °C for 15 to 18 h.

### Efficiency of Plating.

The efficiency of plating for mutant PhiKZ was determined by the plaque assay on K2733 lawns expressing gp210-GFPmut1 as described above. The average titer of wild-type PhiKZ was normalized to one and the efficiency of plating for each mutant was determined relative to the wild type.

### Isolation and Sequencing of gp210-Resistant PhiKZ.

A volume of 100 μL of *P. aeruginosa* K2733 gp210-GFPmut1 (OD_600_ ~0.4) was infected with 10 μL of PhiKZ lysate, and the plaque assay was performed. Isolated plaques were streak purified three times to obtain clonal populations. Plate lysates were generated, and phage genomic DNA was isolated (*SI Appendix*). Whole genome sequencing was performed by SeqCenter (Pittsburgh) using Illumina NextSeq 2000 at a depth of 200Mbp. SeqCenter provided paired-end reads (2x151bp) and reported variations from the Genbank entry for PhiKZ (NC_004629.1).

### Isolation of PicA Mutant PhiKZ with Cas13a.

*P. aeruginosa* K2733 cells expressing Cas13a and the specified guide were grown to OD_600_ ~0.5 in LB containing 15 μg/mL gentamicin and 1% arabinose. Escape mutants were then isolated as described above. The region of the genome targeted was amplified by PCR and sequenced by Sanger sequencing (Azenta). Whole genome sequencing was performed as described for a subset of mutants (PhiKZ PicA T367P and Q297K) to determine that no off-target mutations were generated.

Alternatively, for PicA guide 6, no plaques could be isolated by the above method without first enriching for mutants in liquid culture. A volume of 3 mL of OD_600_ ~0.5 culture was infected with ~10^9^ PFU of PhiKZ and grown overnight at 30 °C rotating. The supernatant was collected and serially diluted until individual plaques could be obtained and purified by the above method.

### Western Blot.

Western blot was performed essentially as described in ref. 45. See *SI Appendix* for detailed methodology.

### Quantification of the Fraction of Cells with Nuclear Localization of sfGFP-Tagged Protein during PhiKZ Infections.

Single-cell infection and fluorescence microscopy were performed as described. The location of the phage genome was determined by the approximately circular area of high intensity in the DAPI channel. Nuclear localization of the sfGFP-tagged protein was called if the highest intensity of GFP signal overlapped with the phage genome. Bias was avoided by quantifying every cell imaged across five fields of imaging per condition (N = 68 to 159 per condition).

### Quantification of Nuclear Cross-Sectional Area.

Single-cell infection and fluorescence microscopy were performed as described. Nuclear cross-sectional area was determined by tracing the border of the DAPI-stained nuclei in ImageJ at its widest point. The area was then measured by ImageJ in µm^2^. Sixty nuclei were measured per condition, areas were plotted with PRISM, and statistical significance was determined by the Mann–Whitney test.

### Quantification of Nuclear Diameter.

Single-cell infection and fluorescence microscopy were performed as described. Infected cells were identified by the presence of cell swelling and host genome degradation. DAPI-stained foci indicative of phage genomes were measured at their widest point using the DeltaVision SoftWoRx 6.5.2 Image Analysis Program. Infected cells were then binned into two categories, those containing a DAPI stained focus greater or less than 0.5 µm. All cells imaged per each condition were quantified to avoid bias. N ranged from 121 to 183 infected cells per condition. Statistical significance was determined by Fisher’s exact test performed as a pairwise comparison between each of the three conditions.

### Single-Step Time to Lysis.

The experiment was performed essentially as described in ref. 43. See *SI Appendix* for detailed methodology.

### Fluorescence Colocalization Analysis.

We used fluorescence microscopy images to perform object-based colocalization analyses of PhiPA3gp108[PhiKZgp104(113-162)]-sfGFP and PicA-mCherry foci in PhiKZ infected cells in eight different fields of view (FOV). Two FOVs were excluded from the analysis due to sample drift. The analysis and corresponding scripts were performed and written in MATLAB (*SI Appendix*). A total of 187 infected cells had fluorescence above threshold and were included in the analysis. (49)

## Supplementary Material

Appendix 01 (PDF)

## Data Availability

Image Analysis Algorithm data have been deposited in Github (https://github.com/koeinlow/Colocalization_analysis_Pogliano_lab/).
