# Supplementary material for: An essential and highly selective protein import pathway encoded by nucleus-forming phage
Source: Proc Natl Acad Sci U S A. 2024 Apr 30;121(19):e2321190121. doi: 10.1073/pnas.2321190121 (PMC11087766; doi:10.1073/pnas.2321190121)
Supplement: Supplementary file 1 — Appendix 01 (PDF) [file pnas.2321190121.sapp.pdf]

PNAS

# Supporting Information for

## An essential and highly selective protein import pathway encoded by nucleus-forming phage

Chase J. Morgan[a], Eray Enustun[a], Emily G. Armbruster[a], Erica A. Birkholz[a], Amy Prichard[a], Taylor Forman[a], Ann Aindow[a], Wichanan Wannasrichan[a,d], Sela Peters[a], Koe Inlow[a], Isabelle L. Shepherd[a], Alma Razavilar[a], Vorrapon Chaikeeratisak[d], Benjamin A. Adler[e,f], Brady F. Cress[f], Jennifer A. Doudna[e-j], Kit Pogliano[a], Elizabeth Villa[a,c], Kevin D. Corbett[a,b], Joe Pogliano[a,]*

*Corresponding author: jpogliano@ucsd.edu 4111 Natural Sciences Building University of California San Diego La Jolla, CA 92093, USA

**This PDF file includes:**

Supporting Text
SI References
Tables S1 to S2
Figures S1 to S15

## Supplementary Methods

### Phage Genomic DNA Isolation

Phage genomic DNA was isolated using 10 mL of lysate first incubated with 5 µl of each RNaseA (100 mg/mL) and DNaseI (20mg/mL) at 37°C for 30 minutes and then 4 ml of phage precipitant (30% PEG 8000, 19.3% NaCl in $ddH_2O$) was added and incubated overnight at 4 °C. Samples were centrifuged at 10,000 rcf 4°C for 20 minutes and resuspended in 0.5 ml sterile water. 2.5 ml of Qiagen Buffer PB was added and incubated at RT for 10 minutes. The resuspensions were then filtered through Qiagen PCR Purification columns as specified by the manufacturer and then eluted with 100 uL of sterile water.

### Fluorescence Colocalization Analysis

https://github.com/koeinlow/Colocalization_analysis_Pogliano_lab/. In general, pairs of images corresponding to a specific FOV showing sfGFP and mCherry fluorescence were globally thresholded and binarized. For 2-D pixel-based object detection, we identify connected components (objects) using 4-connected pixel neighborhoods. Objects which contain less than 5 and more than 150 pixels are removed, and corresponding label matrices are generated for each discrete object. Image pairs are overlaid to create a composite image, and label matrices are again generated for overlapping objects and pixels. Composite images were then overlaid onto DAPI-stained cells to determine the number of infected cells that display overlapping or distinct fluorescence in one or both

channels. Cells were then binned into the phenotypes shown in Figure S5 and the number of cells displaying each phenotype were counted.

**AlphaFold Structural Prediction**

Structural predictions were generated with AlphaFold2 (1) software accessed through Google Colab notebook (https://colab.research.google.com/github/sokrypton/ColabFold/blob/main/AlphaFold2.ipynb). Amino acid sequences used for AlphaFold2 structural predictions were retrieved from the National Center for Biotechnology Information database. Structures were visualized and figures generated with PyMOL software (The PyMOL Molecular Graphics System, Version 2.0 Schrödinger, LLC).

**Phylogenetic Analysis**

All phylogenetic analyses performed compare protein sequences. The genome-wide phylogenetic tree was made using VICTOR (2), and the trees made for individual proteins were made using Clustal Omega 2.1 (3) and annotated in iTOL (4). Percent identity values were calculated from Clustal Omega alignments.

**Single Step Time-to-Lysis**

*P. aeruginosa* K2733 was grown at 37 °C to $OD_{600}$ ~0.25 in LB. 30μL of phage lysate (wildtype or mutant PhiKZ) was added to 3mL of bacteria (MOI = ~5) and incubated at 30°C for 25 minutes then Sytox Green Nucleic Acid Stain (ThermoFisher) was added to a final concentration of 5μM. 200 μL of each culture was added to a well of a black-walled, clear-bottom 96-well plate (Costar), with 12

wells per condition. Fluorescence measurements were performed in a microplate reader (Tecan Infinite M Plex) at 30 °C. Fluorescence measurements were taken at: $\lambda_{excitation}$ = 504 nm; $\lambda_{emission}$ = 537 nm; gain = 25; flashes per well = 5. Measurements began at 30 mpi and were taken every 2 minutes up to 120 minutes post-infection. Data analysis was done with Microsoft Excel and Prism (GraphPad). For each replicate, background fluorescence was subtracted and values were divided by the maximum fluorescence detected. Negative values due to photobleaching of the background prior to lysis were set to zero. The mean +/- the standard deviation across 12 replicates was plotted and average time-to-lysis determined as the time point at which the mean fraction of maximal fluorescence reached 0.5.

**Western blot**

MC1000 strains were infected on 1% agarose, 25% LB, 6 cm diameter plates containing the appropriate antibiotics and inducers. 75 mpi ~4.0 x $10^8$ cells were resuspended in 500 µL 2x SDS loading buffer (0.1 M Tris HCl, 0.004% bromophenol blue, 4% SDS, 20% glycerol, pH 6.8 plus 5% BME) and heated for 4 minutes at 95 °C. 10 µL of each sample were loaded into a Novex 4-20% Tris-Glycine Gel and run at 200V. Protein was transferred to PVDF (Pall Life Sciences) and blocked at room temperature with StartingBlock PBS Blocking Buffer (Thermo Scientific). Membrane was blotted with anti-ChmA (dilution = 1:500; custom polyclonal rabbit antibody generated by GenScript), anti-PicA (dilution = 1:1,000; custom polyclonal rabbit antibody generated by GenScript), and anti-RpoB-HRP (loading control, dilution = 1:5,000; Biolegend) for 1 hour at room temperature. The

membranes were washed with TBS-T and incubated with secondary antibody if indicated (dilution = 1:10,000; HRP-conjugated goat anti-rabbit IgG (H + L), Invitrogen) for 1 hr at room temperature. The membranes were visualized via ECL (Cytiva Amersham) using a ChemiDoc MP Imaging System (Bio-Rad). Images were adjusted for figure panels in Adobe Photoshop (21.2.0) and final figures were generated in Adobe Illustrator (24.2).

**SI References**


1) J. Jumper, et al., Highly accurate protein structure prediction with AlphaFold. *Nature* **596**, 583–589 (2021).
2) J. P. Meier-Kolthoff, M. Göker, VICTOR: genome-based phylogeny and classification of prokaryotic viruses. *Bioinformatics* **33**, 3396–3404 (2017).
3) F. Madeira, et al., Search and sequence analysis tools services from EMBL-EBI in 2022. *Nucleic Acids Res.* **50**, W276–W279 (2022).
4) I. Letunic, P. Bork, Interactive Tree Of Life (iTOL) v5: an online tool for phylogenetic tree display and annotation. *Nucleic Acids Res.* **49**, W293–W296 (2021).

**Supplementary Table 1**
**Cas13 guide RNAs used in this study**

| Guide Name | DNA Sequence | Codons Targeted |
|---|---|---|
| PhiKZ_PicAg1 | ATTATTAAAACCTAACATAGAACTACCTCAA | 1-10 |
| PhiKZ_PicAg2 | TCAGTATTACCACCCATTGCTGAATTATTAA | 4-14 |
| PhiKZ_PicAg3 | TGAGTTTGAGCACGTGGTGCAATCACATCAG | 68-78 |
| PhiKZ_PicAg4 | CTGCAACAGTGTGATGATCTGCACCACCCCA | 360-370 |
| PhiKZ_PicAg5 | TTCGAAGTACAGCAGATGCATCACCTAGCAT | 286-296 |
| PhiKZ_PicAg6 | TTGGAATGGATATCTTGATTTCGAAGTACAG | 292-302 |
| PhiKZ_PicAg7 | GAACTTGGAAAGAGAAATCGTAGGCATTGTT | 547-557 |
| Goslar_PicAg1 | TATTTCACGAACATTCATCGGGATCATTTTA | -4-7 |
| Goslar_ChmAg1 | ACCGTTATTACGTACGTCTAAGCCCATTTGG | -8-3 |
| Non-targeting | AGAGACCTCGTTTACCTATCGGTCTC | N/A |

**Supplementary Table 2**
**Plasmid constructs used in this study**

| Backbone | Insert |
|---|---|
| pHERD30T | PhiPA3_RecA-sfGFP |
| | PhiPA3_gp108-sfGFP |
| | PhiPA3_gp200-sfGFP |
| | PhiPA3_gp78-sfGFP |
| | PhiPA3_gp257-sfGFP |
| | PhiKZ_RecA-sfGFP |
| | PhiKZ_gp104-sfGFP |
| | PhiKZ_gp171-sfGFP |
| | PhiKZ_gp104(1-113)-sfGFP |
| | PhiKZ_gp104(66-113)-sfGFP |
| | PhiKZ_gp104(66-162)-sfGFP |
| | PhiPA3_gp108[PhiKZ_gp104(1-65)]-sfGFP |
| | PhiPA3_gp108[PhiKZ_gp104(66-162)]-sfGFP |
| | PhiPA3_gp108[PhiKZ_gp104(66-115)]-sfGFP |
| | PhiPA3_gp108[PhiKZ_gp104(66-95)]-sfGFP |
| | PhiPA3_gp108[PhiKZ_gp104(77-100)]-sfGFP |
| | PhiPA3_gp108[PhiKZ_gp104(66-76)]-sfGFP |
| | PhiPA3_gp108[PhiKZ_gp104(79-88)]-sfGFP |
| | PhiPA3_gp108[PhiKZ_gp104(96-115)]-sfGFP |
| | PhiPA3_gp210-GFPmut1 |
| | PhiPA3_gp210-gp104 |
| | GFPmut1 |
| | PhiKZ_PicA + GFPmut1 |
| | PhiKZ_PicA-sfGFP |
| | PhiPA3_PicA-sfGFP |
| | Lbu_Cas13a_PicAg1 |
| | Lbu_Cas13a_PicAg2 |

| | |
|---|---|
| | Lbu_Cas13a_PicAg3 |
| | Lbu_Cas13a_PicAg4 |
| | Lbu_Cas13a_PicAg5 |
| | Lbu_Cas13a_PicAg6 |
| | Lbu_Cas13a_PicAg7 |
| | PhiKZ_PicA-mCherry + PhiPA3gp108[PhiKZgp104(113-162)]-sfGFP |
| pDSW206 | Goslar_PicA-sfGFP_codonaltered(1-30) |
| | Goslar_PicA_codonaltered(1-30) |
| p15A-CmR | dCas13d_Goslar_ChmAg1 |
| | dCas13d_Goslar_PicAg1 |
| | dCas13d_Non-targeting |

A

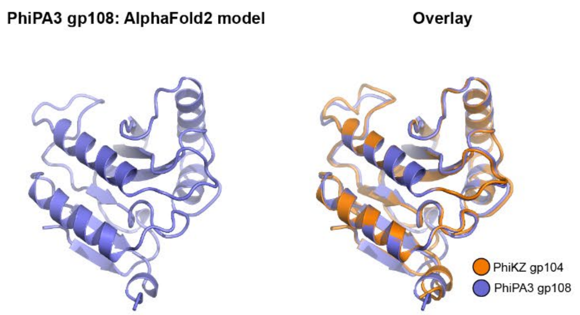


B

```
PhiKZ gp104   TKDNWRNPSQISFIERGLE
PhiPA3 gp108  TKERWQDPSLPSYISAGLQ
```

C

```
PhiPA3_gp200    MDSIKDLLADPLKGTKQAQGVLCYLFREVLLWRKINWFTWNRRQKAYFEKPHNAVNPDKG  60
PhiKZ_gp171     MESVKDLLSDPQKGLRQAEGVLCYVFREVLLWRNMTQFAWDKRLNAYFQKPHNKNNPDKG  60
                *:*:****:** ** :**:*****:********::. *:*::* :***:****  *****

PhiPA3_gp200    NLNKALKADDMNWPGFKKGIDFLNPESATLEVKLNWTHKE-STSYVIVIDPTEDEKNPTV  119
PhiKZ_gp171     NLNKALKADTMSWDGFKKAIDFLSPVKATLEVRLIWGTSKPDSVYTINIDPTEDEVTCSA  120
                ********* *.* ****.****.* .*****:* *   .: .: *.* *******  . :.

PhiPA3_gp200    NNFPWQDCPIFKSAKPAETLMSHLFRHIVAEEGKGEPDIMVWWEKKFDDYIKNPVNVVGL  179
PhiKZ_gp171     NDFPYEECEIFKNQKPATTTMAHLYRHILWKEGINQE----KWDKLWEEFLNNPVNLVGL  176
                *:**:::* ***. *** * *:**:***: :** .:       *:* :::::****:***

PhiPA3_gp200    NQKEINQNIAALRRSLMEARMSWNVFRRGLHLLRPRSEEYILTLHWTTDPEMKKTLPDSI  239
PhiKZ_gp171     DKKKLTSLANTQRRGLLSPNMSWNTLRRGFLLLRPKEERYTLSLQWTDDPNLRDKLEDSV  236
                ::*::..   : **.*:. .****.:***: ****:.*.* *:*:** **:::..* **:

PhiPA3_gp200    HPIKIADPYFVE-----     251
PhiKZ_gp171     HIVPVPDPHWVEPNSNE     253
                * : : **::**
```

D

```
PhiPA3_RecA     MFAKHFERPAFRPALNIGCLMDVSTGKYEQGKHGEMIMNGGLGSLTGIASRPNNFKTALG   60
PhiKZ_RecA      MFGKHFERPAFRPALNIGCLMDISTGKYEQGKHGEMILNGGMGSLTGIASRPNNFKTALG   60
                **.*******************:**************:***:******************

PhiPA3_RecA     VYMLAMVRRAFPGSYSMVYDTEGTLNPDARFTALAASIKELQGINWADDEQFVFTDLSRY   120
PhiKZ_RecA      IYLMAMMRRAFPGAHAMTYDTEGTLNPGARFTSLAQAYPELAAIDWENDEQYMFTDLSRY   120
                :*::**:******:::*.*********.****:** :  ** .*:* :***::*******

PhiPA3_RecA     TGDEFFKLFRTALAEKEKAEKDHLRTTPFLDVNGNNKKCLYPTTGFIDSFSKFIVTAVSD   180
PhiKZ_RecA      TGDEWFKIFRDALSEKEKAEKDYLRTTPFLDVNGNNKKALYPTGGFIDSFSKFIVSAVSE   180
                ****:**:** **:********:**************.**** **********:***:

PhiPA3_RecA     MYEKNAIGASGNNTDAMTNGKAKNQLFNQLPQVCAKTGTYMILTAHVGDIIQMEMYPTDK   240
PhiKZ_RecA      MYAKNAIGDSKVNTDAMTNGKAKNQLFNQLPQICAKTSTYMILTAHVGDIIQIEMYPTDK   240
                ** ***** *  *******************:****.**************:*******

PhiPA3_RecA     RNLSEMKKDTVLKGVSSGFYSLPNNVWDVMSNKPLLNKDKMPVYPLDNSTAIEGDSDLRI   300
PhiKZ_RecA      RNLSHMKKDTVLKGVSGGFYSLPNNVFSIESNKPLVNKEKMPLYPIDNSTAIEGDTDLRI   300
                ****.***********.*********:.: *****:**:***:**:*********:****

PhiPA3_RecA     LEVKNLRGKGGITGLPFTLIVSQTEGIMPSLSEFHYCKEAGYGIGGNLQNYYLELMPDVK   360
PhiKZ_RecA      LEVINLRGKGGITGLPFHLIVSQTEGILPSLSEFHYCRENDWGIGGNNINYFVELCPDIK   360
                *** ************* *********:*********:* .:*****  **::** **:*

PhiPA3_RecA     LSRTTVRKKLNDNPALQRAVEIQSEMLQLIQFQRWTD---VPDPKELYEGLKAMGYDWDV   417
PhiKZ_RecA      LSRTTVRKKLMDNPALQRAVEIQSEMLQLIQFQRWNLGDYVVTPAELYADLKVMGYDWDI   420
                ********** *************************.    *  * *** .**.******:

PhiPA3_RecA     ILNQTRGYWVCEEDEHLVEKKFLSTFDLIRMLRSEYKPYWMSDADKAKIIPLDLAKAAA   476
PhiKZ_RecA      ILNNTRGYWMFEEDEQFSDKKFLSTYDLLRMRKSEYKPYWMTDEEKAKIVPLELAKAKA   479
                ***:*****: ****:: :******:**:** :********:* :****:**:**** *
```

**Figure S1**

**Structural and sequence alignments of PhiKZ and PhiPA3 nuclear proteins**

A) AlphaFold predicted structures of PhiPA3 gp108 (left) and structures of PhiKZ gp104 and PhiPA3 gp108 aligned with PyMol (right). RMSD = 0.487 Angstrom.

B) PhiKZ gp104 amino acids 77-95 aligned with the corresponding region in PhiPA3 gp108. Conserved amino acids are in green. Non-conserved amino acids are in black with red highlighting.

C) Clustal Omega generated sequence alignment of PhiPA3 gp200 and PhiKZ gp171.

D) Clustal Omega generated sequence alignment of PhiPA3 RecA and PhiKZ RecA.

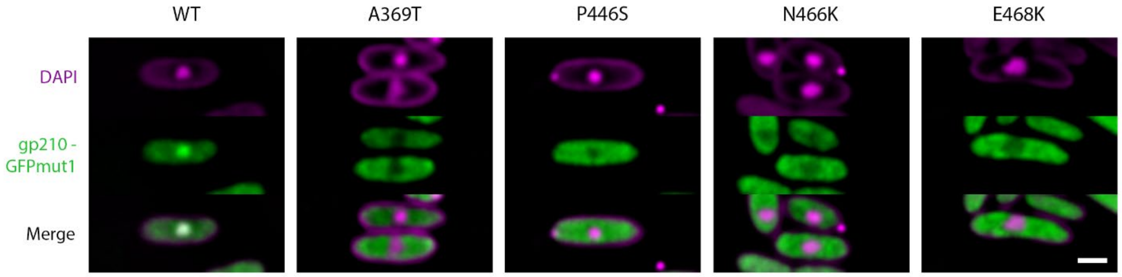


**Figure S2**

**PicA mutant PhiKZ that excludes GFPmut1 also exclude PhiPA3 gp210-GFPmut1**

PicA wildtype or mutant PhiKZ infecting *P. aeruginosa* cells expressing PhiPA3 gp210-GFPmut1. Scale bar is 1 µm.

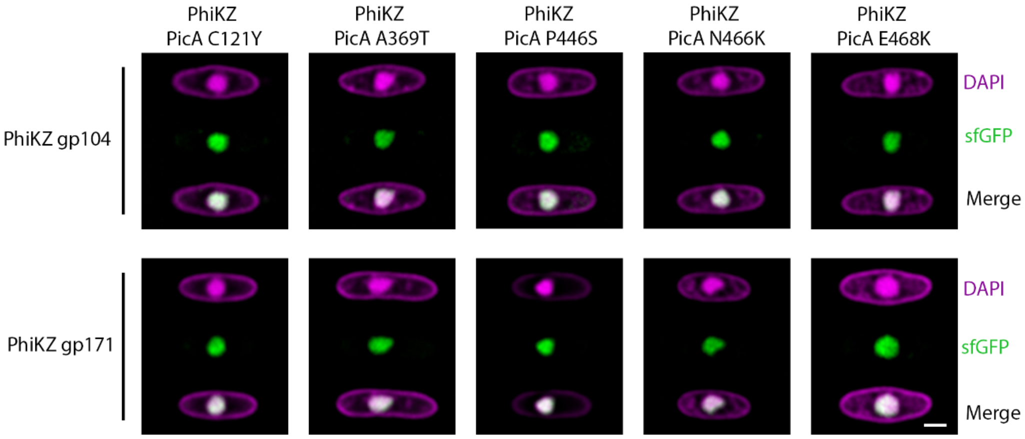


**Figure S3**

**PicA mutant PhiKZ that exclude GFPmut1 still import phage proteins**

PicA mutant PhiKZ infecting *P. aeruginosa* cells expressing sfGFP-tagged PhiKZ gp104 (upper panels) or PhiKZ gp171 (lower panels). Scale bar is 1 µm.

A

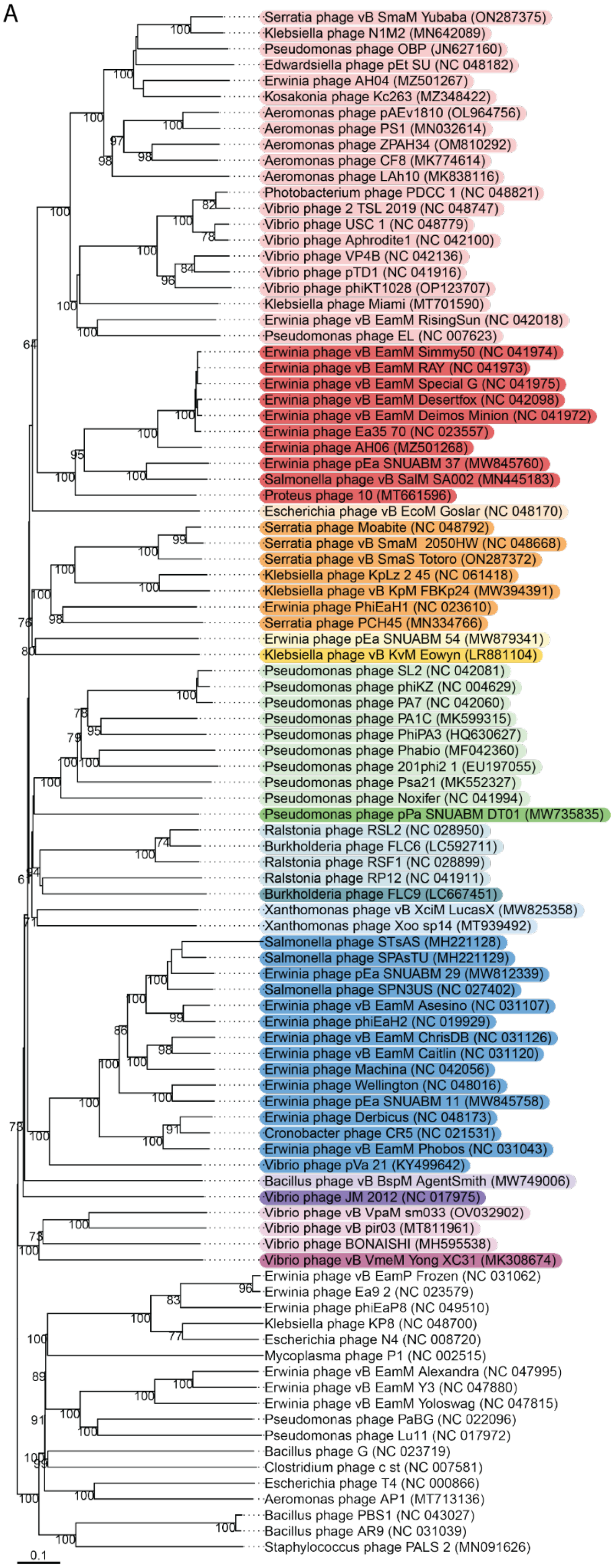


B

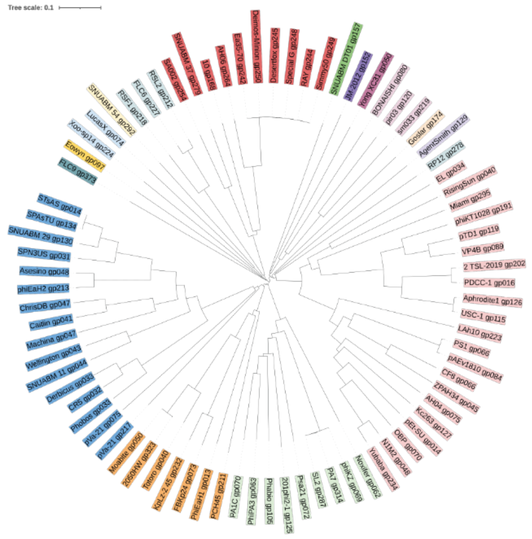


C

| | PhiKZ | PhiPA3 | Goslar |
|---|---|---|---|
| PhiKZ | 100.00 | 52.32 | 20.46 |
| PhiPA3 | 52.32 | 100.00 | 23.32 |
| Goslar | 20.46 | 23.32 | 100.00 |

**Figure S4**
**Phylogenetic Analysis of PicA**

(A) A phylogenetic tree comparing the whole genomes of all chimalliviruses, showing that they form a single clade. As PicA is part of the core genome of chimalliviruses, homologs are encoded in all members of this group. Chimalliviruses are colored in rainbow by VICTOR-predicted genus groups.

(B) A phylogenetic tree of PicA homologs. In general, the protein tree is congruent with the genome tree with the two exceptions of *Erwinia* phage SNUABM_54 clustering with *Xanthomonas* phages Xoo-sp14 and LucasX rather than by itself as a singleton and *Ralstonia* phage RP12 being separate from *Ralstonia* phages RSL2 and RSF1 and *Burkholderia* phage FLC6. Interestingly, *Vibrio* phage pVa-21 has two homologs of this protein, one with a long tail and one without.

(C) A comparison of the % identity of the amino acid sequence of the PicA homologs from PhiKZ (gp69), PhiPA3 (gp63), and Goslar (gp174).

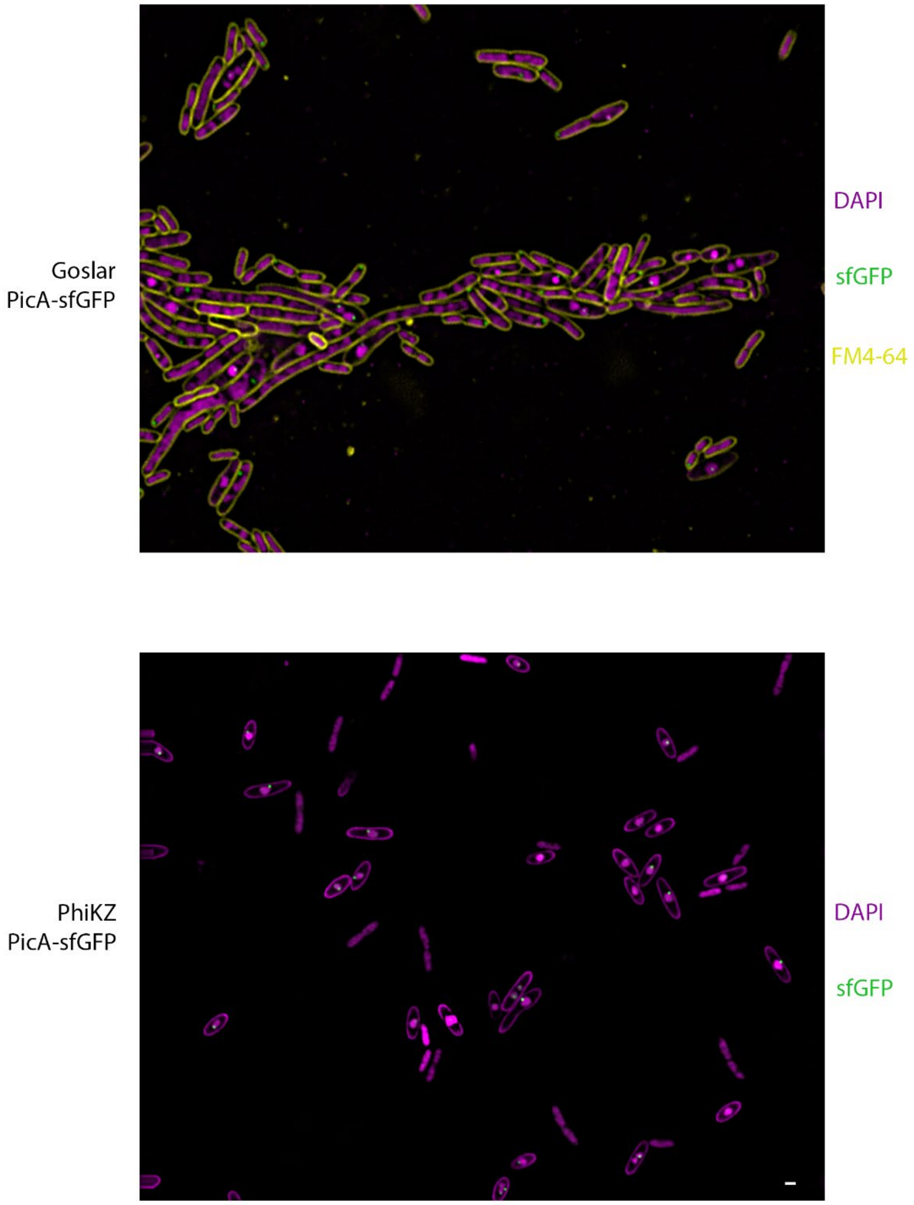


**Figure S5**
**Cells expressing PicA-sfGFP infected with phage**
*E. coli* cells expressing PicA-sfGFP infected with Goslar (upper panel) or *P. aeruginosa* cells expressing PicA-sfGFP infected with PhiKZ (lower panel). Scale bar is 1 µm.

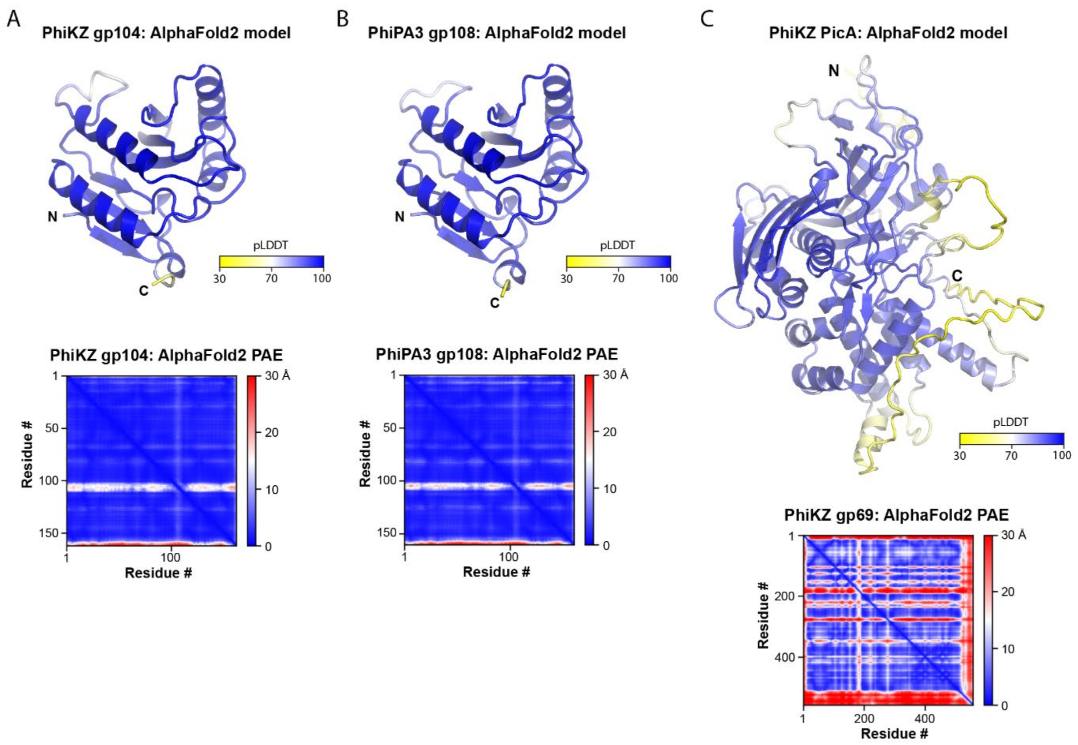


**Figure S6**

**AlphaFold2 predicted structures and predicted aligned errors**

Alphafold2 predicted structure colored by pLDDT and predicted aligned error chart for PhiKZ gp104 (A), PhiPA3 gp108 (B) and PhiKZ PicA (C).

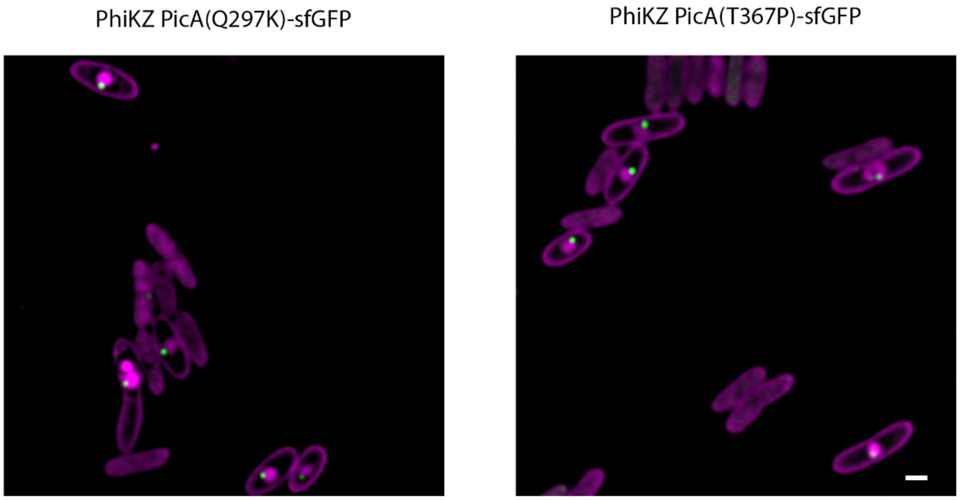


**Figure S7**
**Cells expressing PicA-sfGFP mutants infected with PhiKZ**
*P. aeruginosa* cells expressing sfGFP-tagged PicA with the indicated mutation infected with PhiKZ. Images taken 30-45 mpi. Scale is 1 µm.

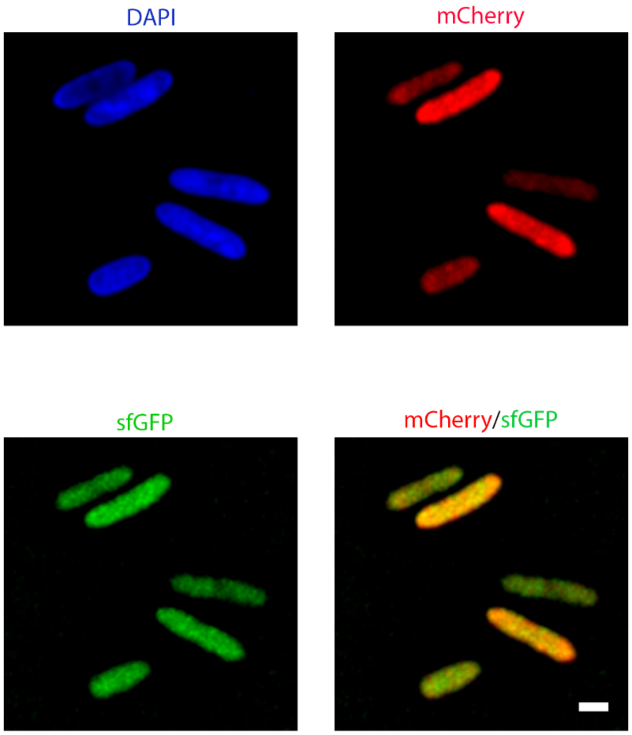


**Figure S8**
**PicA-mCherry and PhiPA3gp108-[PhiKZ gp104(113-162)]-sfGFP are diffuse in uninfected cells.**
Microscopy image of *P. aeruginosa* cells expressing both PicA-mCherry and PhiPA3gp108-[PhiKZ gp104(113-162)]-sfGFP. Scale bar is 1 µm.

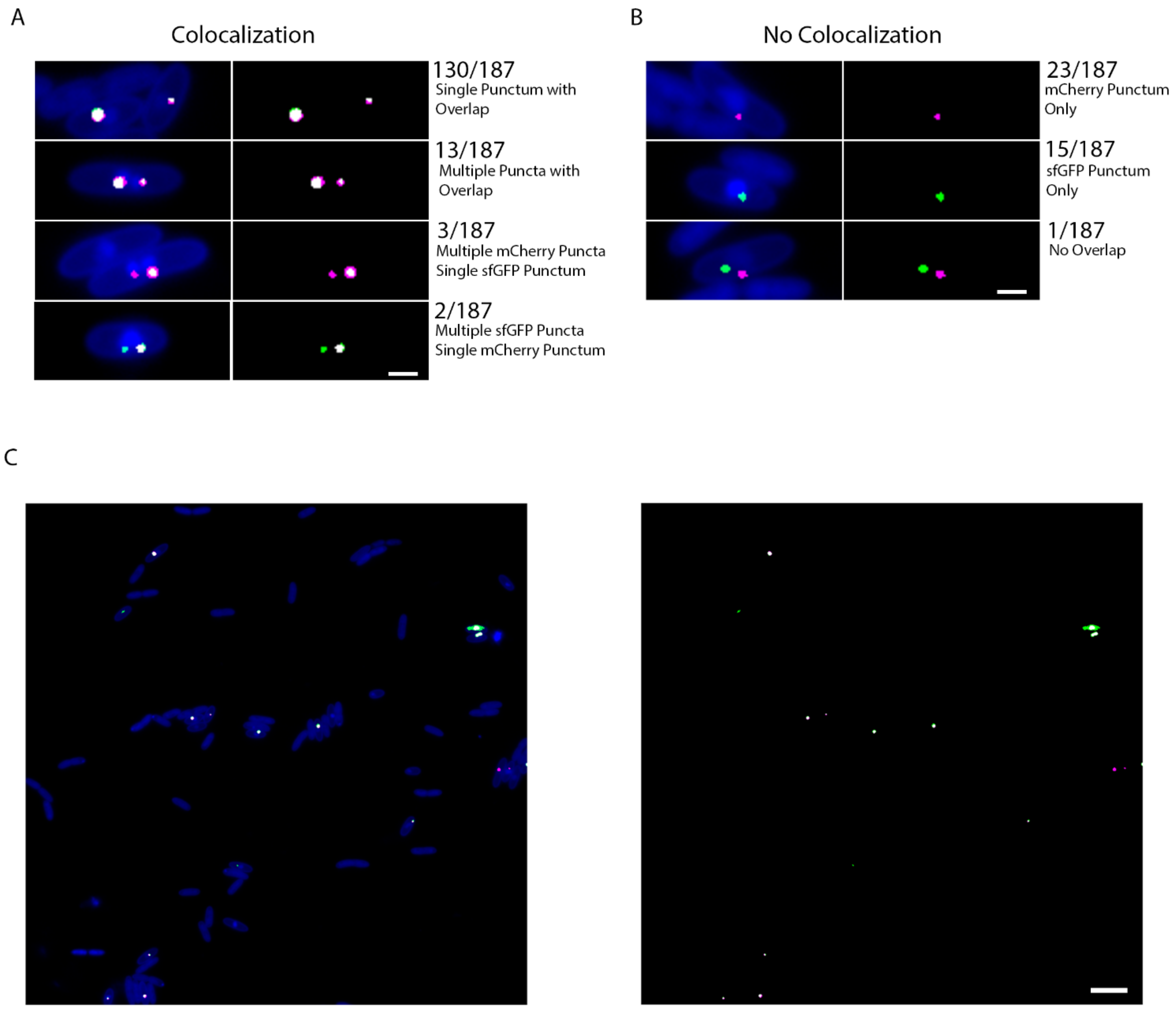


**Figure S9**
**Colocalization Analysis of PhiPA3gp108-(PhiKZ gp104 113-162)-sfGFP and PicA-mCherry**
A) *P. aeruginosa* cells co-expressing the chimera PhiPA3gp108-(PhiKZ gp104 113-162)-sfGFP and PicA-mCherry infected with PhiKZ imaged at 30-45 minutes post infection. Images were analyzed by a non-biased object-based colocalization algorithm and then grouped into the colocalization phenotypes shown. The total number of cells with that phenotype is listed on the right. DAPI is shown in blue, sfGFP signal above threshold is shown in green, mCherry above threshold is shown in magenta. Pixels that contain both mCherry and sfGFP above threshold are white. Total number of infected cells with fluorescence above threshold is 187. Scale bar is 1 µm.
B) Same as described in (A) except phenotypes shown for cells that did not have overlapping fluorescence are shown. Scale bar is 1 µm.

C) Representative whole microscopy field. A total of 8 microscopy fields were analyzed. 2 microscopy fields were left out of the analysis due to drift between sampling of fluorescence channels. Scale bar is 5 µm.

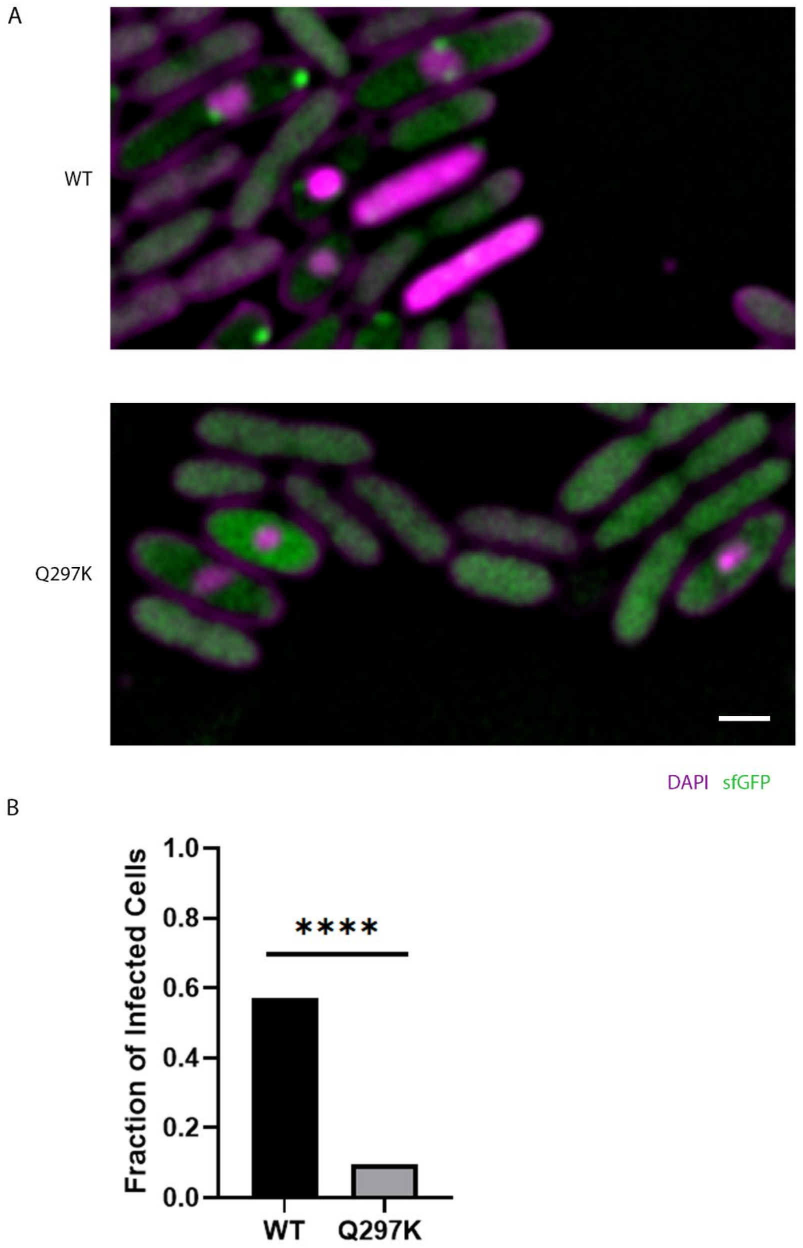


**Figure S10**
**PicA Q297K mutant PhiKZ shows reduced accumulation of PhiPA3gp108-[PhiKZ gp104(113-162)]-sfGFP at the nuclear periphery**
(A) Wildtype (upper panel) or PicA Q297K mutant (lower panel) PhiKZ infecting *P. aeruginosa* expressing PhiPA3gp108-[PhiKZ gp104(113-162)]-sfGFP. Scale bar is 1 µm. (B) Quantification of the fraction of infected cells displaying PhiPA3gp108-[PhiKZ gp104(113-162)]-sfGFP accumulation at the nuclear periphery. **** = $p < .0001$ as determined by Fisher's exact test. N = 232 infected cells for wildtype and 175 infected cells for mutant.

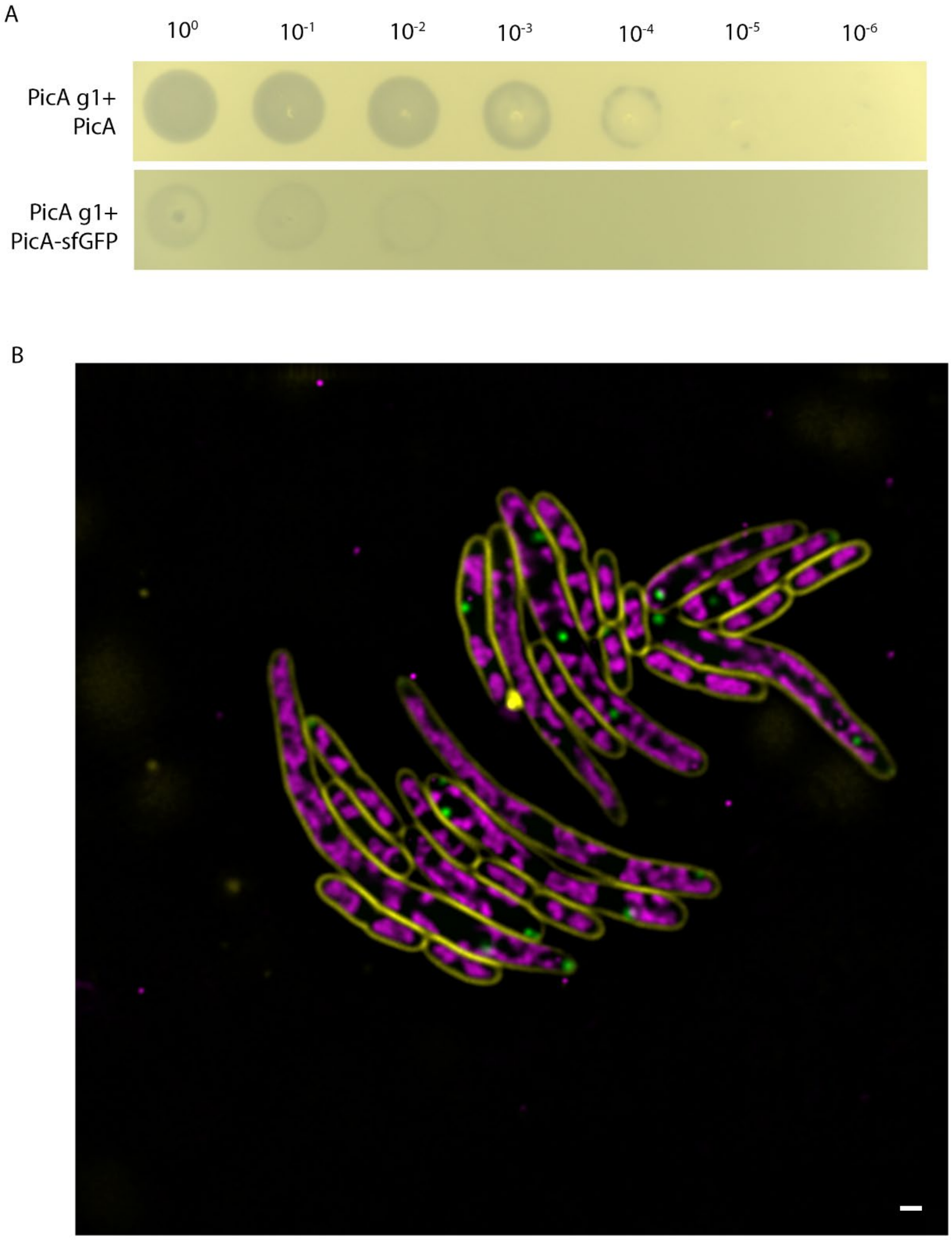


**Figure S11**
**PicA-sfGFP fails to rescue dCas13d knockdown of Goslar PicA**
(A) Spot titer of a serial dilution of Goslar on a lawn of *E. coli* expressing dCas13d with a guide RNA targeting PicA and either codon-altered PicA or codon-altered sfGFP-tagged PicA.
(B) Microscopy image of *E. coli* expressing dCas13d with a guide RNA targeting PicA and codon altered sfGFP-tagged PicA infected with Goslar at 60 mpi. Note that sfGFP signal localizes to the phage genome, but that infection does not progress. DAPI-stained

DNA is purple, sfGFP is green, and FM4-64-stained membrane is yellow. Scale bar is 1 μm.

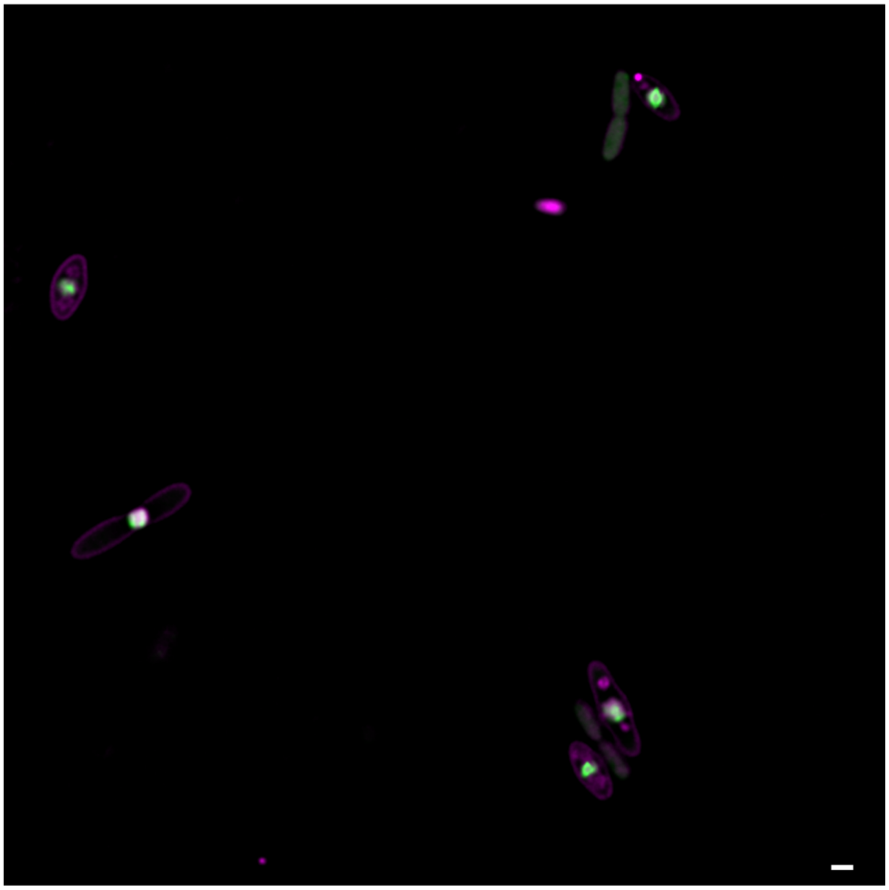

Construct: PhiPA3 RecA-sfGFP
Phage: PhiPA3

**Figure S12**
**Large field of view images corresponding to Figure 1 images**
Microscopy images of *P. aeruginosa* cells expressing the indicated construct and infected with the indicated phage. sfGFP signal is shown in green and DAPI signal is shown in purple. Scale bars are 1 µm.

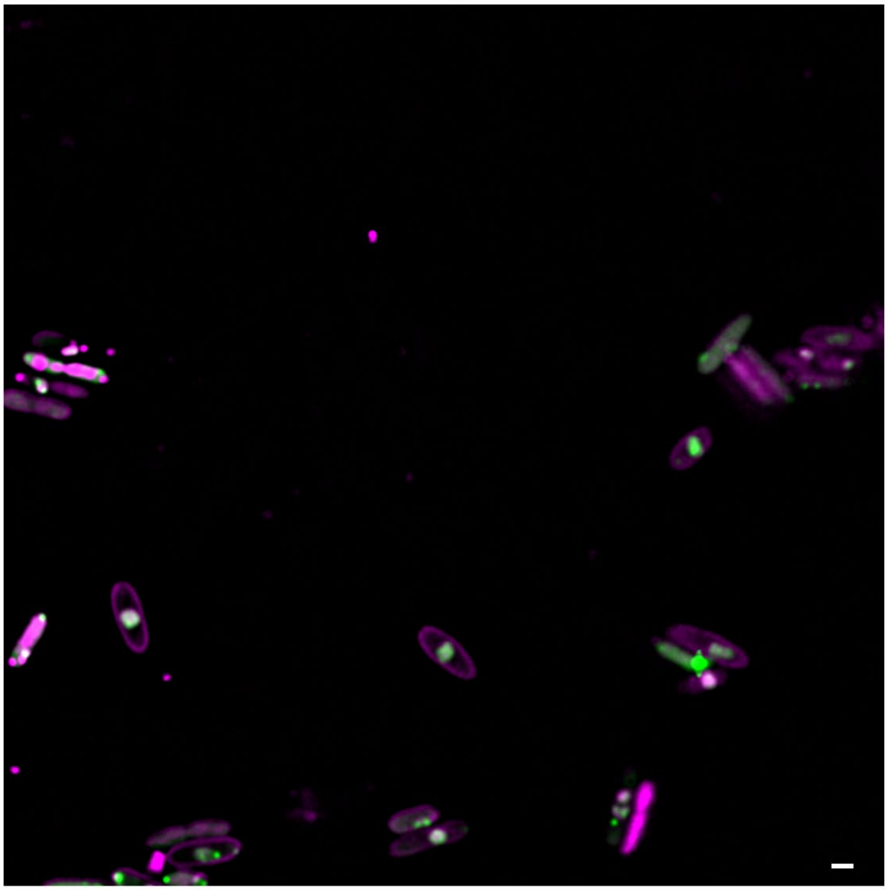

Construct: PhiPA3 RecA-sfGFP
Phage: PhiKZ

**Figure S12 (continued)**
**Large field of view images corresponding to Figure 1 images**
Microscopy images of *P. aeruginosa* cells expressing the indicated construct and infected with the indicated phage. sfGFP signal is shown in green and DAPI signal is shown in purple. Scale bars are 1 µm.

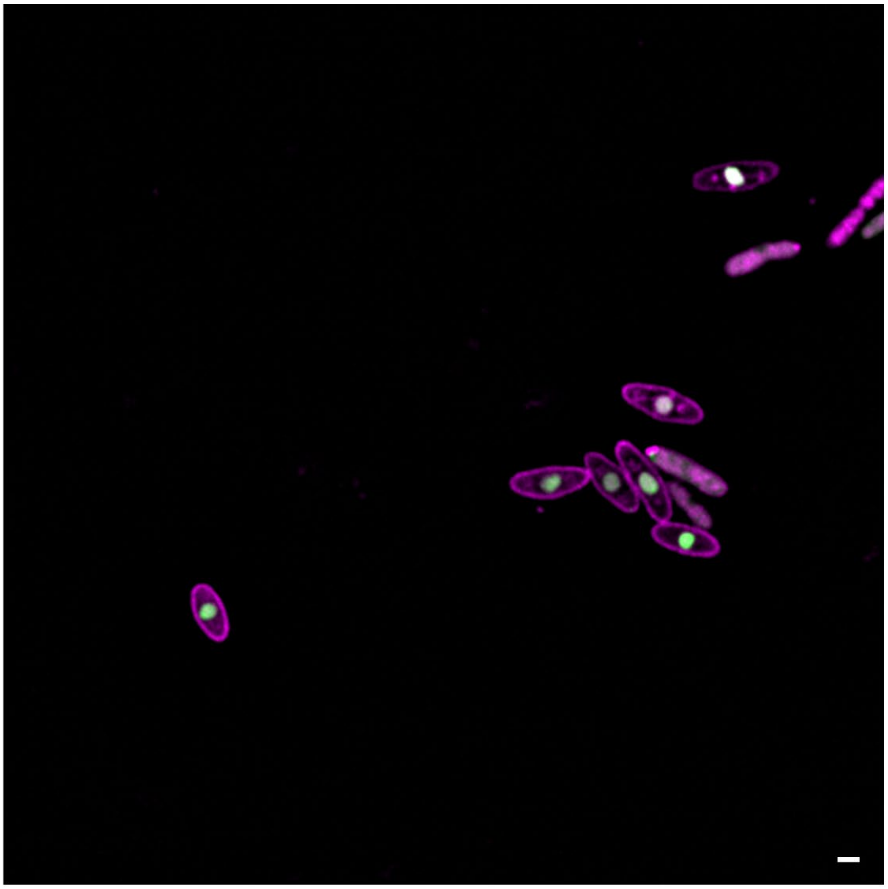

Construct: PhiPA3 gp108-sfGFP
Phage: PhiPA3

**Figure S12 (continued)**
**Large field of view images corresponding to Figure 1 images**
Microscopy images of *P. aeruginosa* cells expressing the indicated construct and infected with the indicated phage. sfGFP signal is shown in green and DAPI signal is shown in purple. Scale bars are 1 µm.

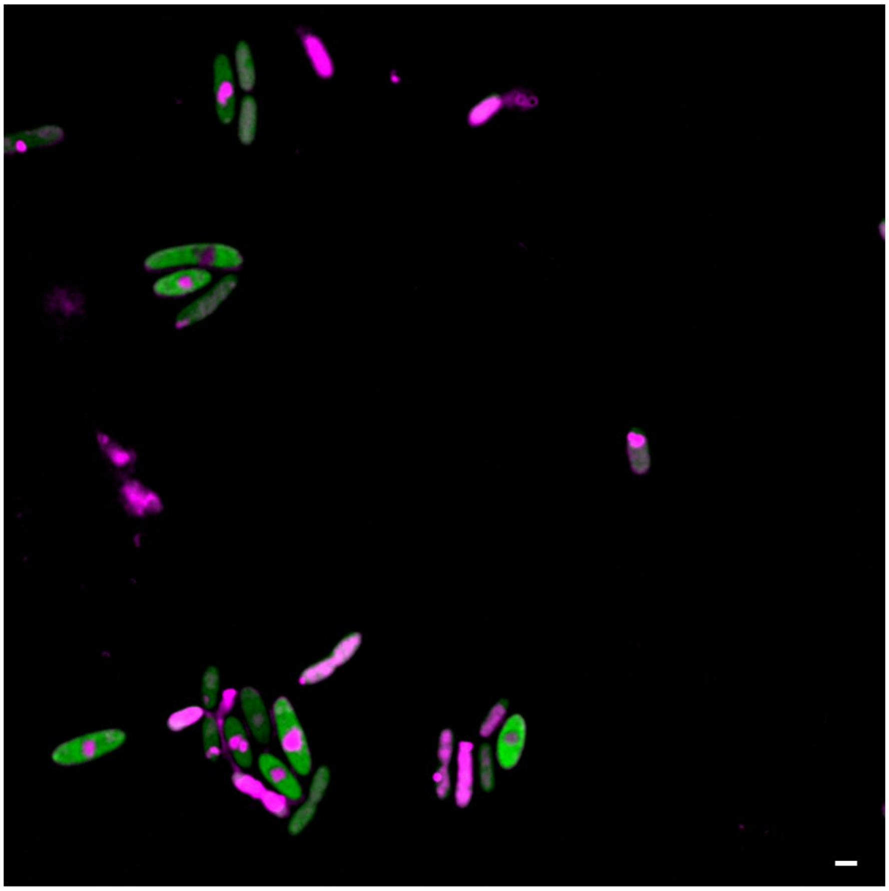

Construct: PhiPA3 gp108-sfGFP
Phage: PhiKZ

**Figure S12 (continued)**
**Large field of view images corresponding to Figure 1 images**
Microscopy images of *P. aeruginosa* cells expressing the indicated construct and infected with the indicated phage. sfGFP signal is shown in green and DAPI signal is shown in purple. Scale bars are 1 μm.

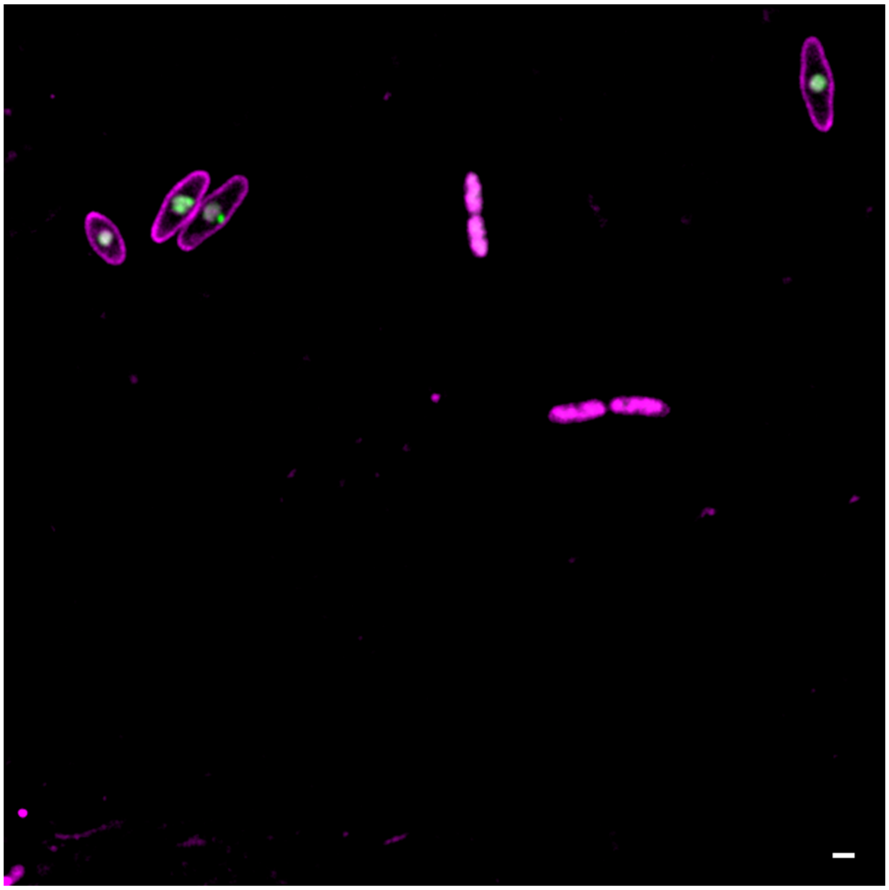

Construct: PhiPA3 gp78-sfGFP
Phage: PhiPA3

**Figure S12 (continued)**
**Large field of view images corresponding to Figure 1 images**
Microscopy images of *P. aeruginosa* cells expressing the indicated construct and infected with the indicated phage. sfGFP signal is shown in green and DAPI signal is shown in purple. Scale bars are 1 μm.

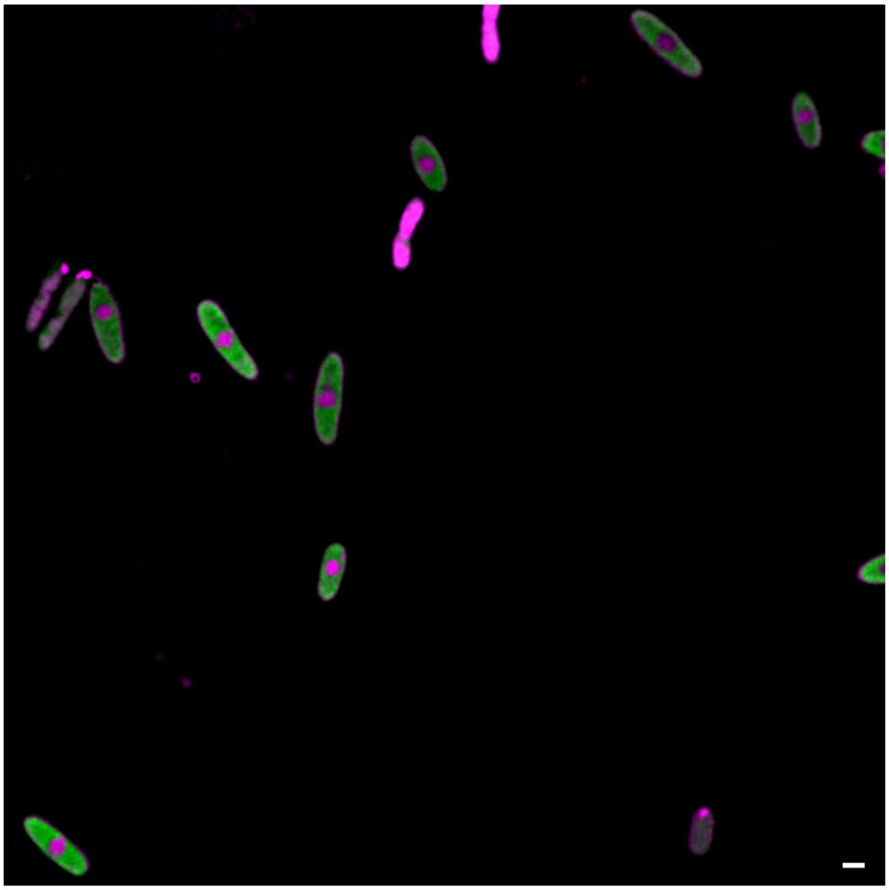

Construct: PhiPA3 gp78-sfGFP
Phage: PhiKZ

**Figure S12 (continued)**
**Large field of view images corresponding to Figure 1 images**
Microscopy images of *P. aeruginosa* cells expressing the indicated construct and infected with the indicated phage. sfGFP signal is shown in green and DAPI signal is shown in purple. Scale bars are 1 µm.

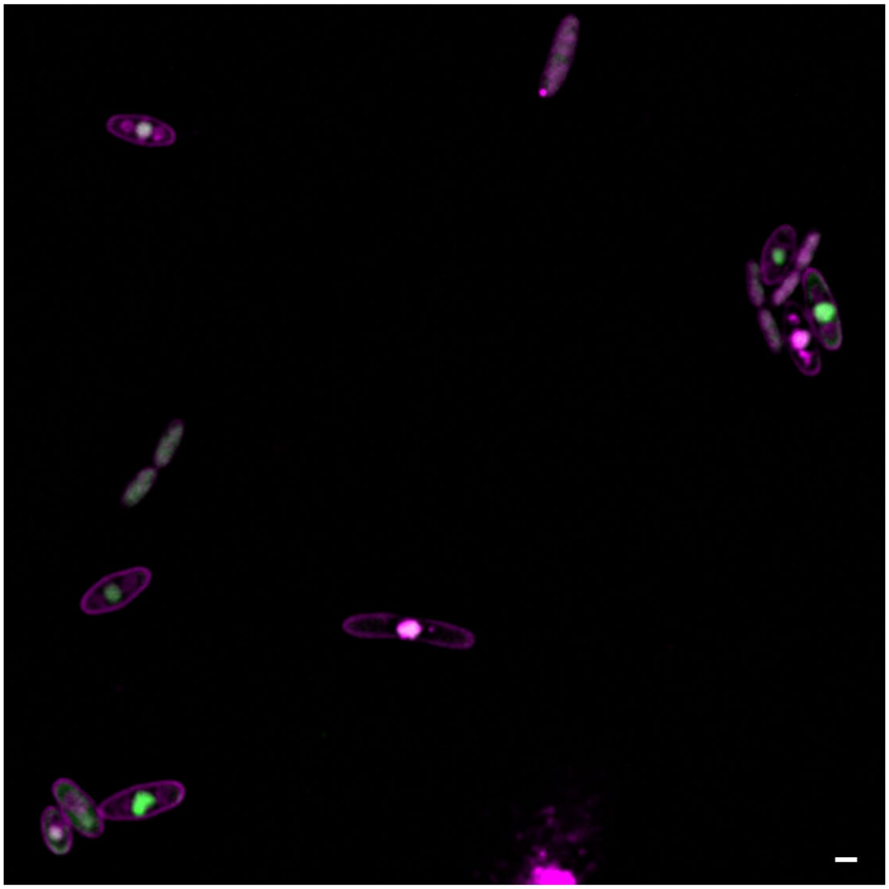

Construct: PhiPA3 gp257-sfGFP
Phage: PhiPA3

**Figure S12 (continued)**
**Large field of view images corresponding to Figure 1 images**
Microscopy images of *P. aeruginosa* cells expressing the indicated construct and infected with the indicated phage. sfGFP signal is shown in green and DAPI signal is shown in purple. Scale bars are 1 μm.

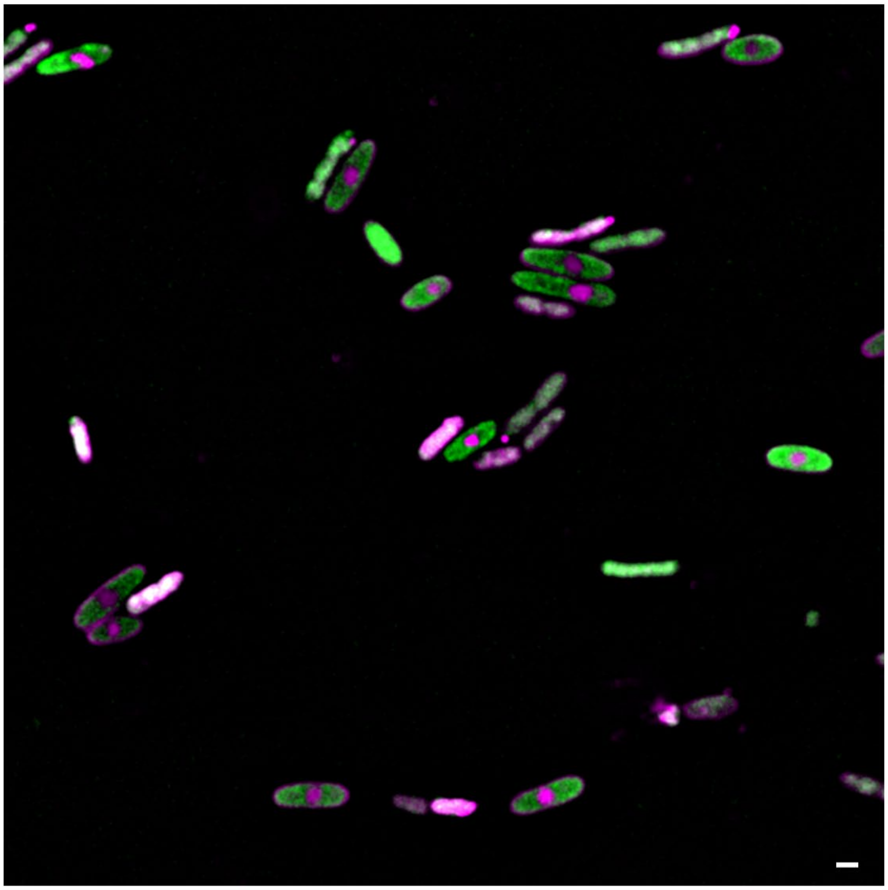

Construct: PhiPA3 gp257-sfGFP
Phage: PhiKZ

**Figure S12 (continued)**
**Large field of view images corresponding to Figure 1 images**
Microscopy images of *P. aeruginosa* cells expressing the indicated construct and infected with the indicated phage. sfGFP signal is shown in green and DAPI signal is shown in purple. Scale bars are 1 µm.

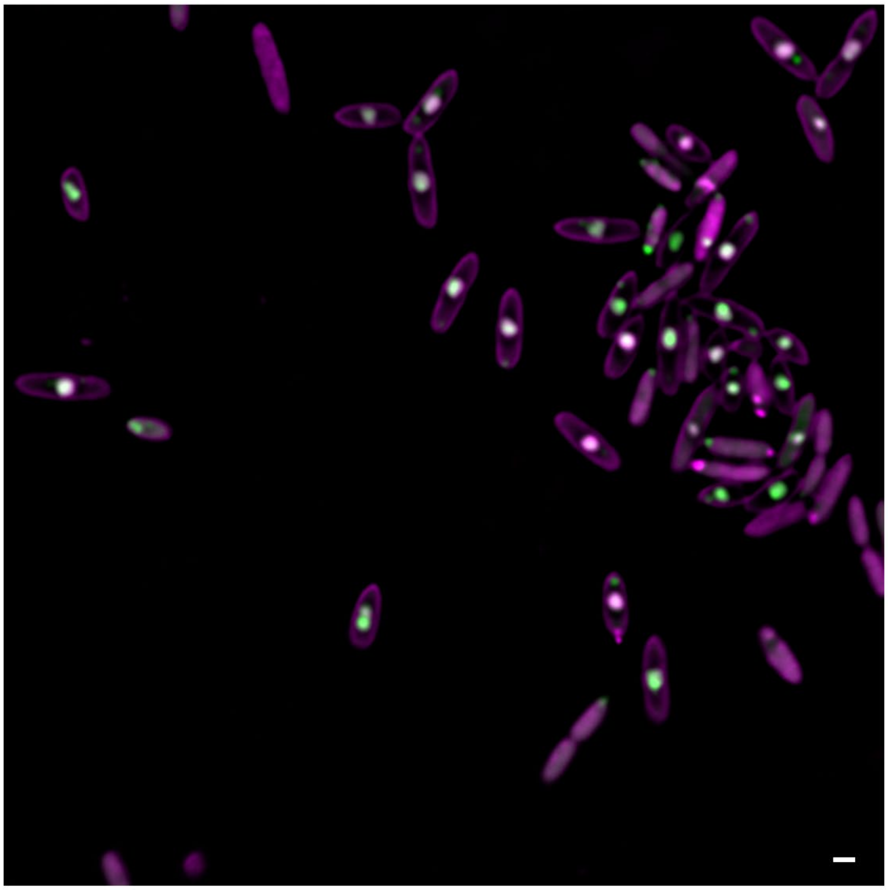

Construct: PhiKZ RecA-sfGFP
Phage: PhiPA3

**Figure S12 (continued)**
**Large field of view images corresponding to Figure 1 images**
Microscopy images of *P. aeruginosa* cells expressing the indicated construct and infected with the indicated phage. sfGFP signal is shown in green and DAPI signal is shown in purple. Scale bars are 1 μm.

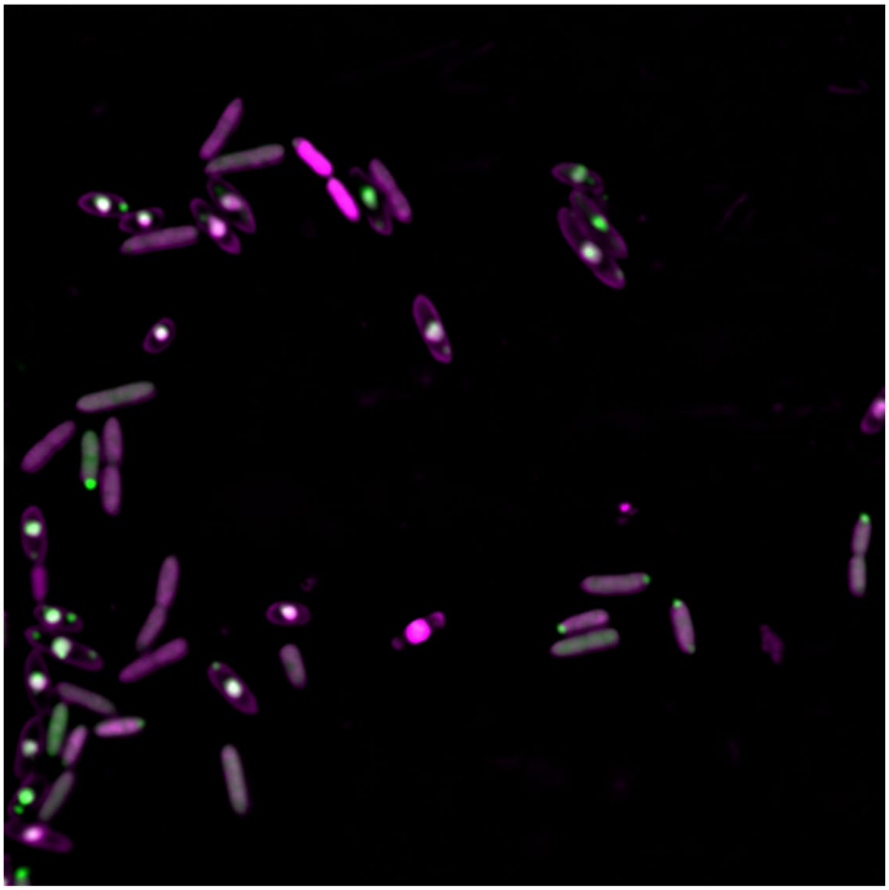

Construct: PhiKZ RecA-sfGFP
Phage: PhiKZ

**Figure S12 (continued)**
**Large field of view images corresponding to Figure 1 images**
Microscopy images of *P. aeruginosa* cells expressing the indicated construct and infected with the indicated phage. sfGFP signal is shown in green and DAPI signal is shown in purple. Scale bars are 1 μm.

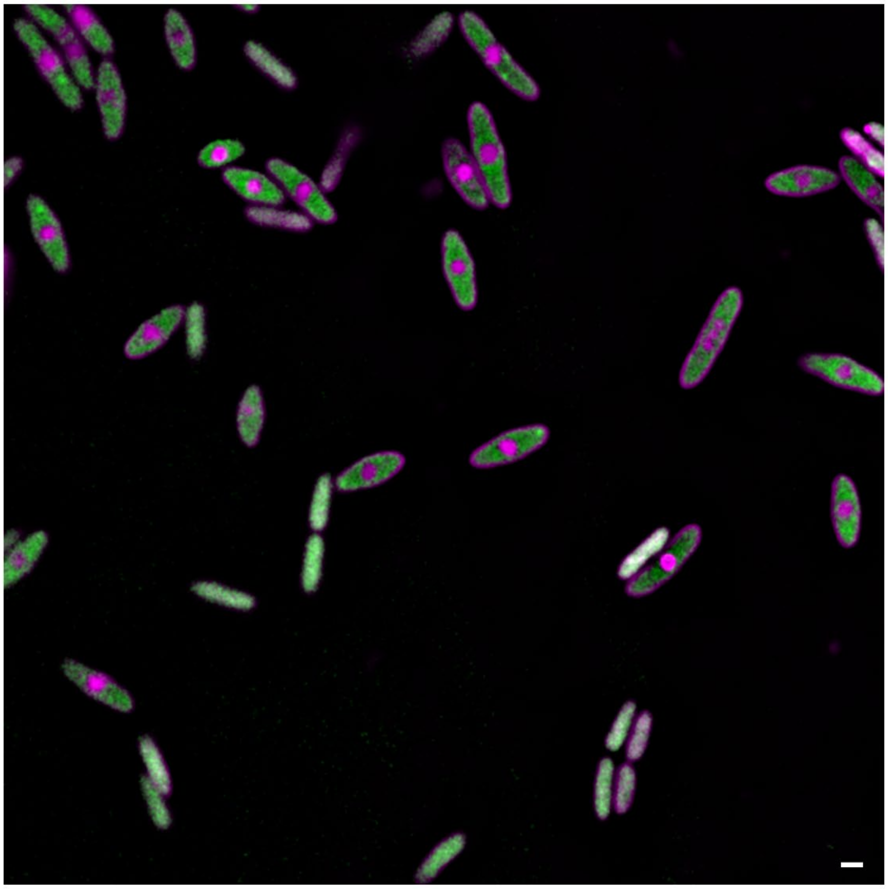

Construct: PhiKZ gp104-sfGFP
Phage: PhiPA3

**Figure S12 (continued)**
**Large field of view images corresponding to Figure 1 images**
Microscopy images of *P. aeruginosa* cells expressing the indicated construct and infected with the indicated phage. sfGFP signal is shown in green and DAPI signal is shown in purple. Scale bars are 1 µm.

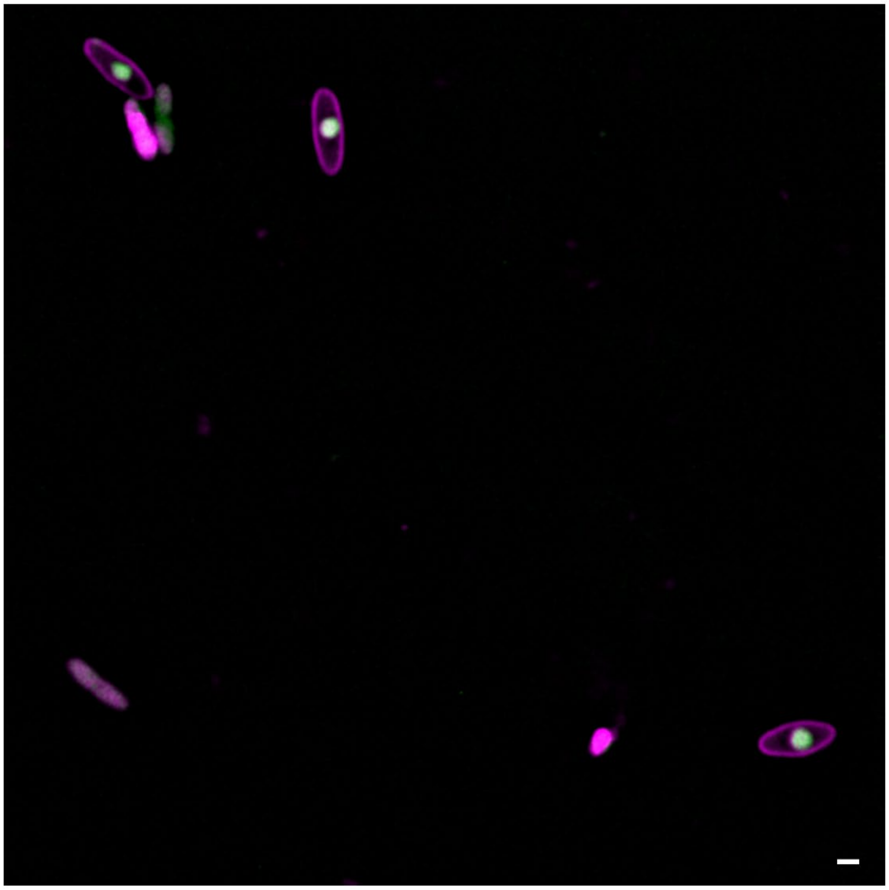

Construct: PhiKZ gp104-sfGFP
Phage: PhiKZ

**Figure S12 (continued)**
**Large field of view images corresponding to Figure 1 images**
Microscopy images of *P. aeruginosa* cells expressing the indicated construct and infected with the indicated phage. sfGFP signal is shown in green and DAPI signal is shown in purple. Scale bars are 1 µm.

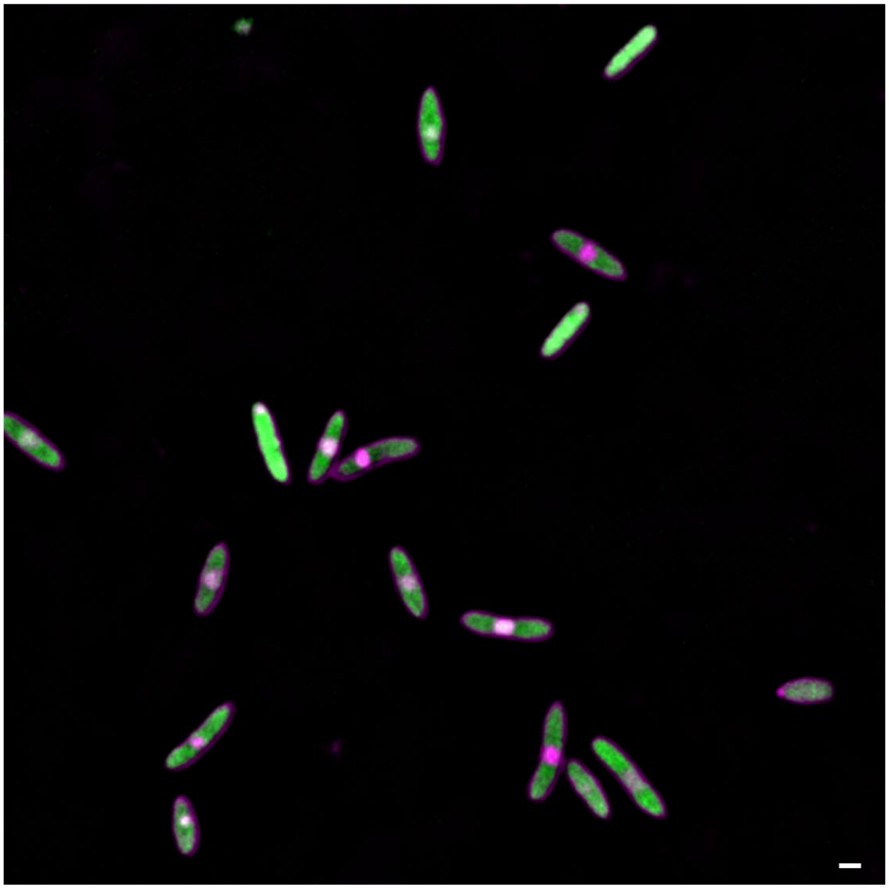

Construct: PhiKZ gp171-sfGFP
Phage: PhiPA3

**Figure S12 (continued)**
**Large field of view images corresponding to Figure 1 images**
Microscopy images of *P. aeruginosa* cells expressing the indicated construct and infected with the indicated phage. sfGFP signal is shown in green and DAPI signal is shown in purple. Scale bars are 1 µm.

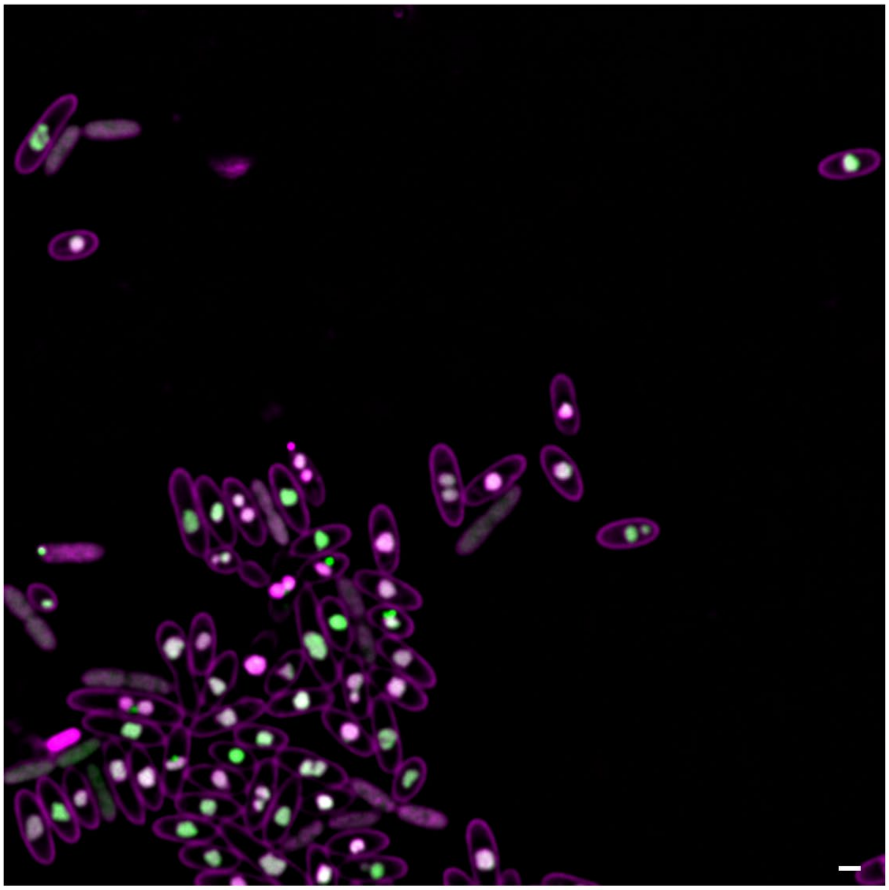

Construct: PhiKZ gp171-sfGFP
Phage: PhiKZ

**Figure S12 (continued)**
**Large field of view images corresponding to Figure 1 images**
Microscopy images of *P. aeruginosa* cells expressing the indicated construct and infected with the indicated phage. sfGFP signal is shown in green and DAPI signal is shown in purple. Scale bars are 1 μm.

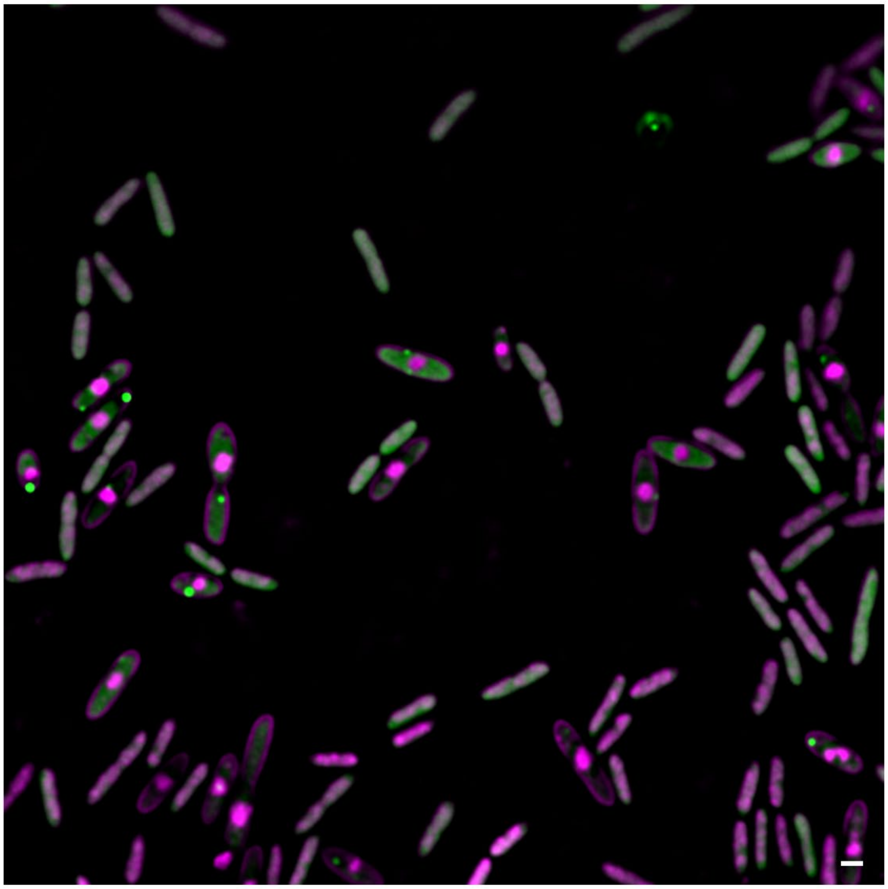

Construct: PhiKZ gp104(1-113)-sfGFP (Truncation)
Phage: PhiKZ

**Figure S12 (continued)**
**Large field of view images corresponding to Figure 1 images**
Microscopy images of *P. aeruginosa* cells expressing the indicated construct and infected with the indicated phage. sfGFP signal is shown in green and DAPI signal is shown in purple. Scale bars are 1 µm.

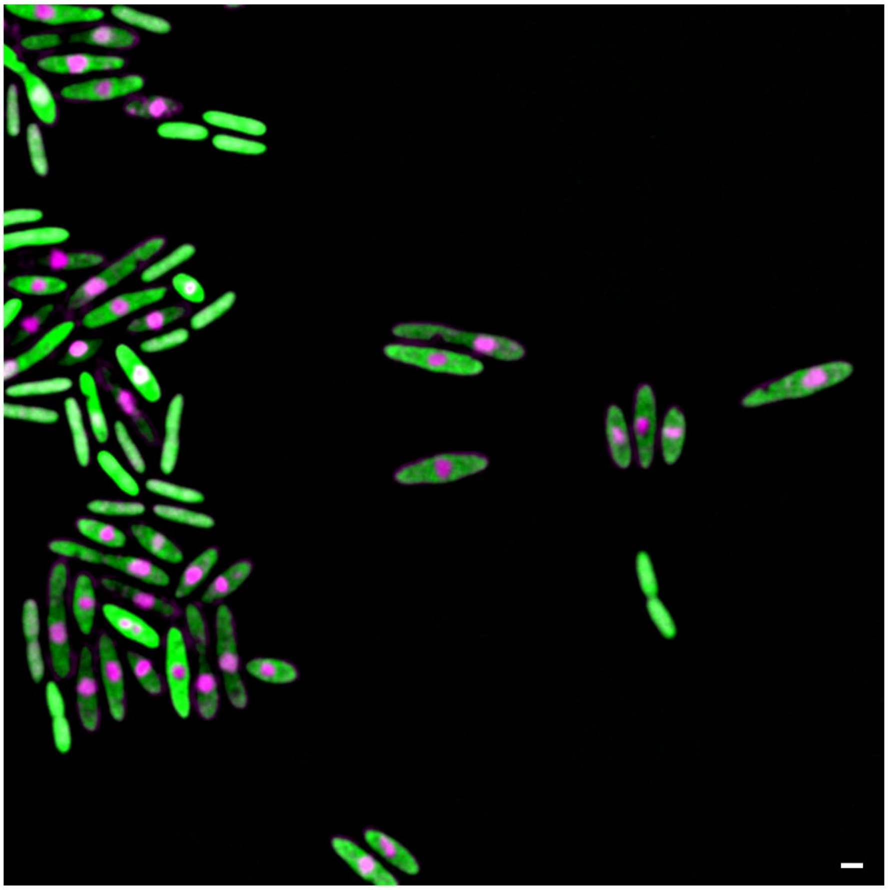

Construct: PhiKZ gp104(66-113)-sfGFP (Truncation)
Phage: PhiKZ

**Figure S12 (continued)**
**Large field of view images corresponding to Figure 1 images**
Microscopy images of *P. aeruginosa* cells expressing the indicated construct and infected with the indicated phage. sfGFP signal is shown in green and DAPI signal is shown in purple. Scale bars are 1 μm.

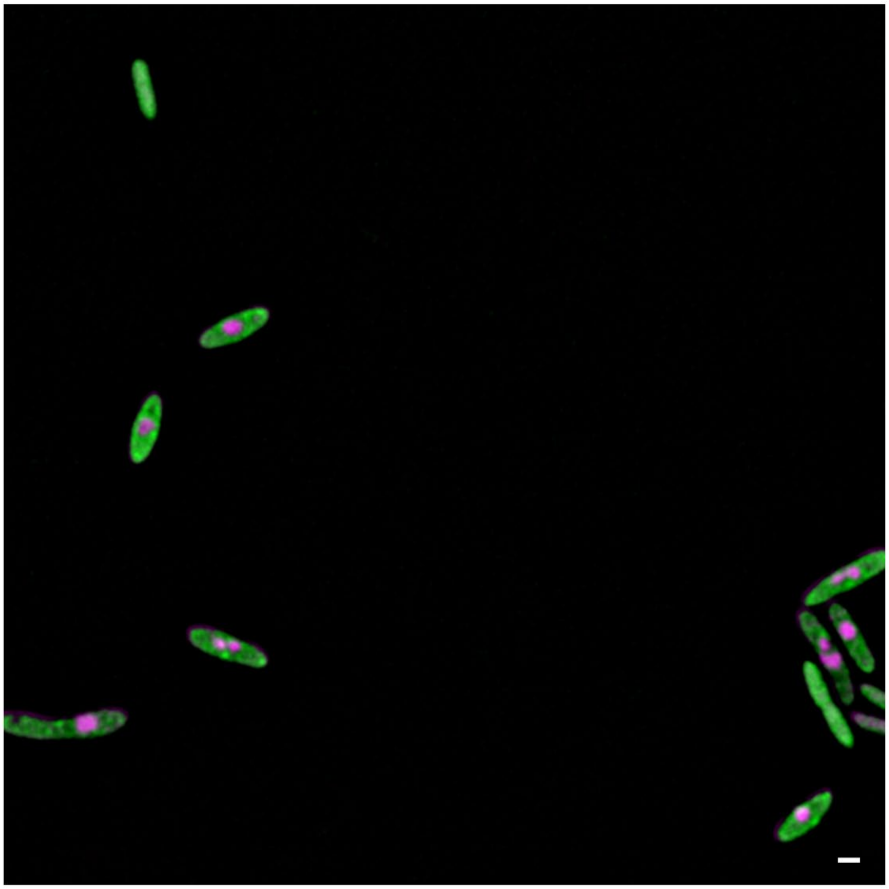

Construct: PhiKZ gp104(66-162)-sfGFP (Truncation)
Phage: PhiKZ

**Figure S12 (continued)**
**Large field of view images corresponding to Figure 1 images**
Microscopy images of *P. aeruginosa* cells expressing the indicated construct and infected with the indicated phage. sfGFP signal is shown in green and DAPI signal is shown in purple. Scale bars are 1 µm.

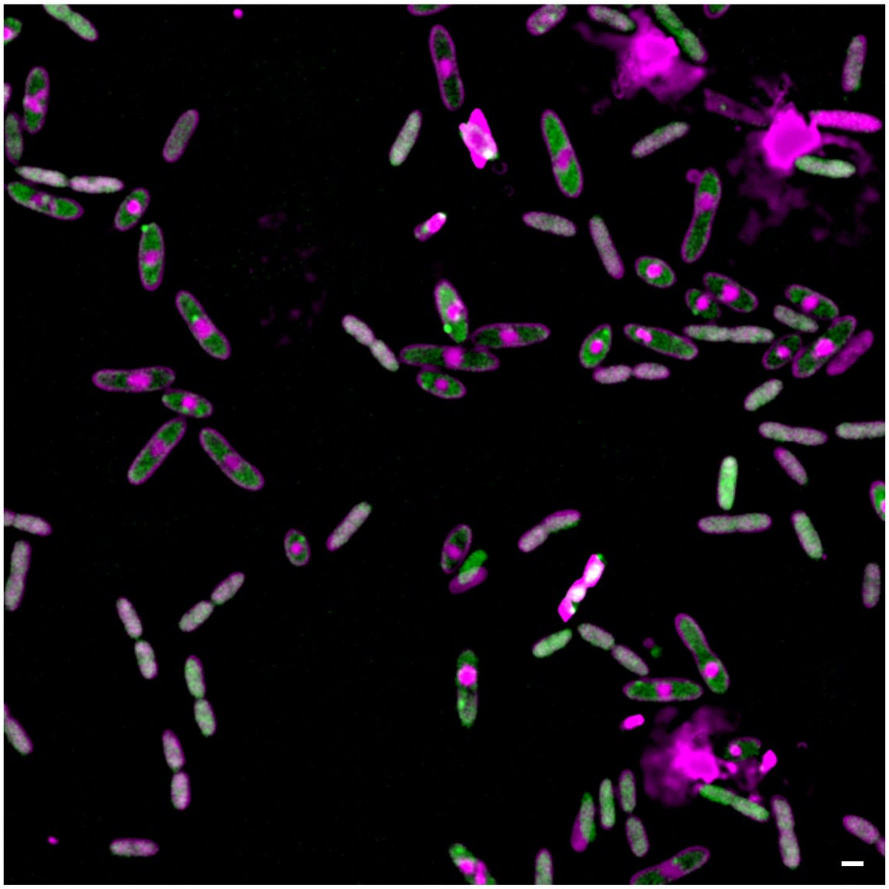

Construct: PhiPA3gp108[PhiKZgp104(1-65)]-sfGFP (Swap)
Phage: PhiKZ

**Figure S12 (continued)**
**Large field of view images corresponding to Figure 1 images**
Microscopy images of *P. aeruginosa* cells expressing the indicated construct and infected with the indicated phage. sfGFP signal is shown in green and DAPI signal is shown in purple. Scale bars are 1 μm.

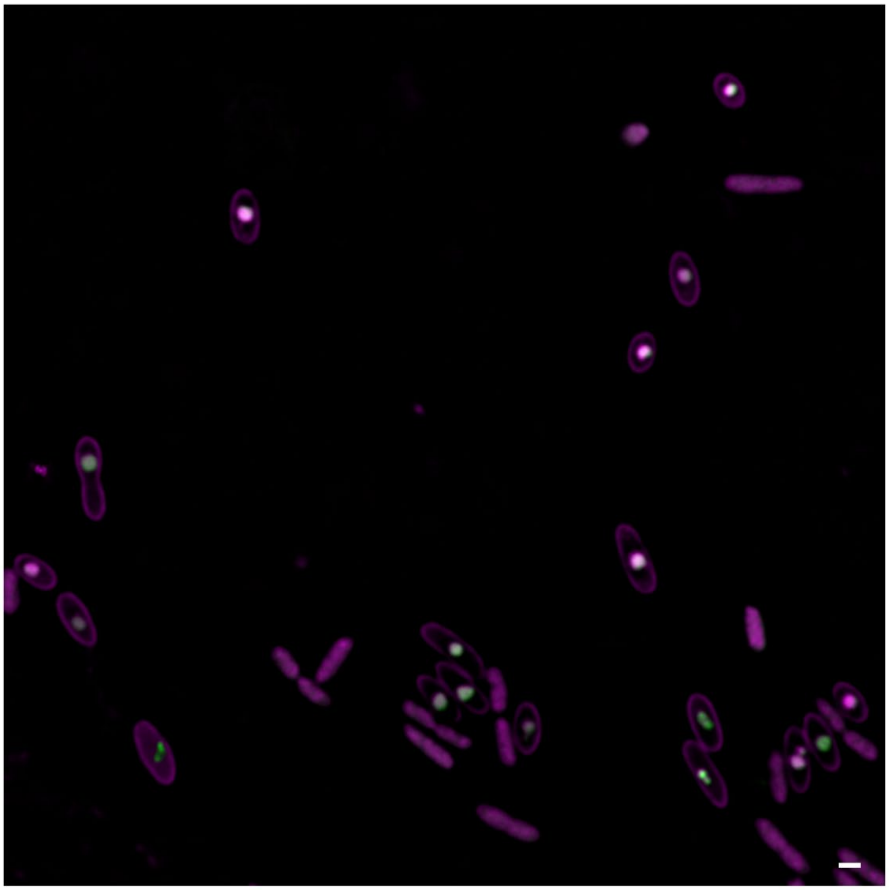

Construct: PhiPA3gp108[PhiKZ gp104(66-162)]-sfGFP (Swap)
Phage: PhiKZ

**Figure S12 (continued)**
**Large field of view images corresponding to Figure 1 images**
Microscopy images of *P. aeruginosa* cells expressing the indicated construct and infected with the indicated phage. sfGFP signal is shown in green and DAPI signal is shown in purple. Scale bars are 1 µm.

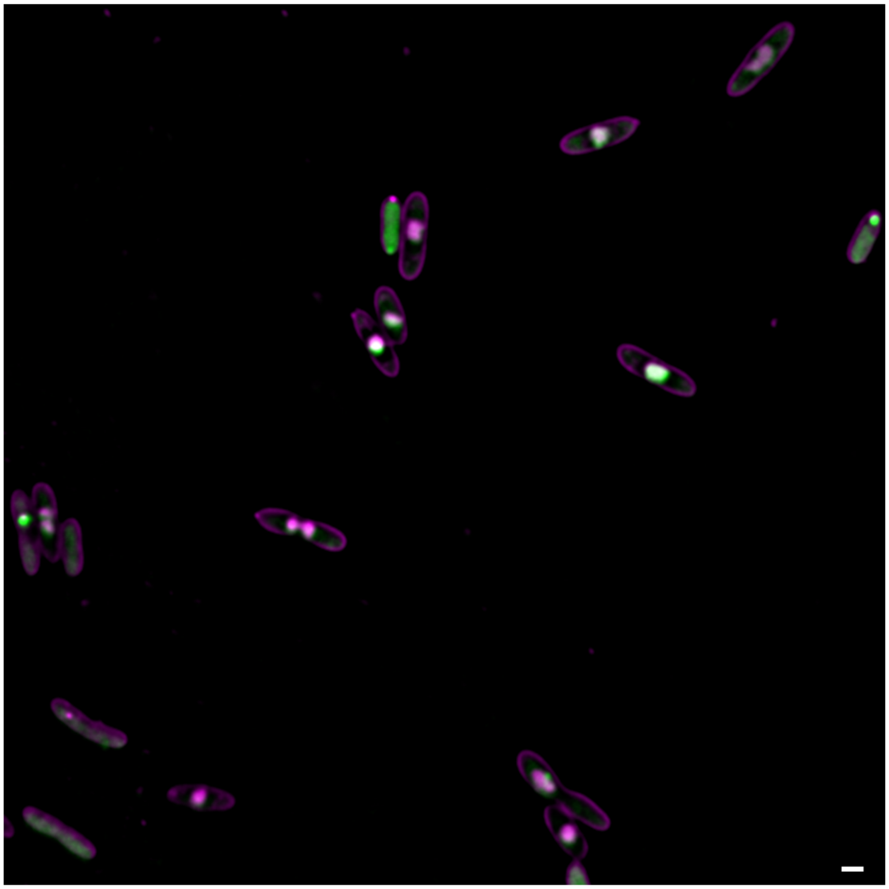

Construct: PhiPA3 gp108[PhiKZ gp104(66-115)]-sfGFP
Phage: PhiKZ

**Figure S12 (continued)**
**Large field of view images corresponding to Figure 1 images**
Microscopy images of *P. aeruginosa* cells expressing the indicated construct and infected with the indicated phage. sfGFP signal is shown in green and DAPI signal is shown in purple. Scale bars are 1 µm.

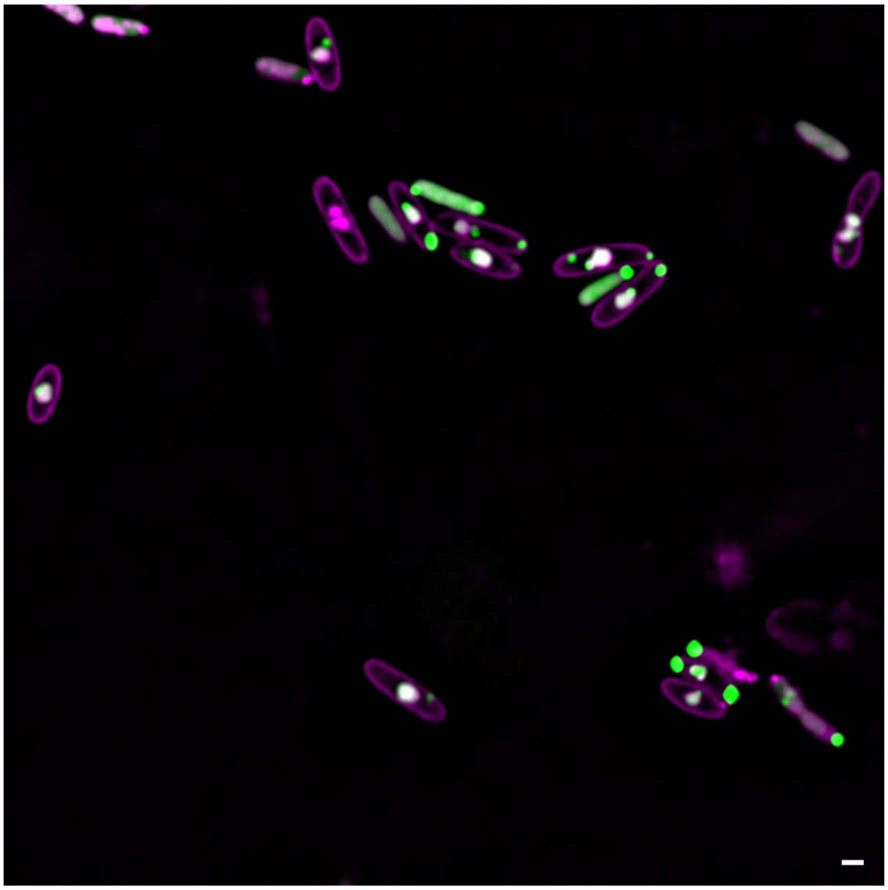

Construct: PhiPA3 gp108[PhiKZ gp104(66-95)]-sfGFP (Swap)
Phage: PhiKZ

**Figure S12 (continued)**
**Large field of view images corresponding to Figure 1 images**
Microscopy images of *P. aeruginosa* cells expressing the indicated construct and infected with the indicated phage. sfGFP signal is shown in green and DAPI signal is shown in purple. Scale bars are 1 µm.

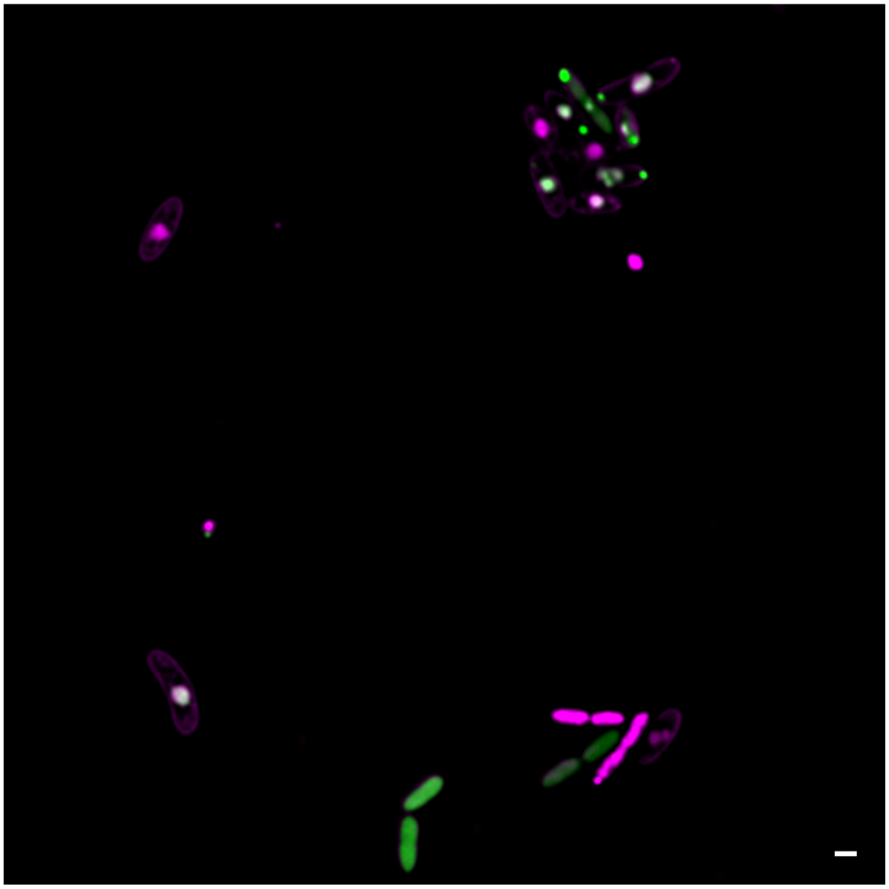

Construct: PhiPA3 gp108[PhiKZ gp104(77-100)]-sfGFP (Swap)
Phage: PhiKZ

**Figure S12 (continued)**
**Large field of view images corresponding to Figure 1 images**
Microscopy images of *P. aeruginosa* cells expressing the indicated construct and infected with the indicated phage. sfGFP signal is shown in green and DAPI signal is shown in purple. Scale bars are 1 μm.

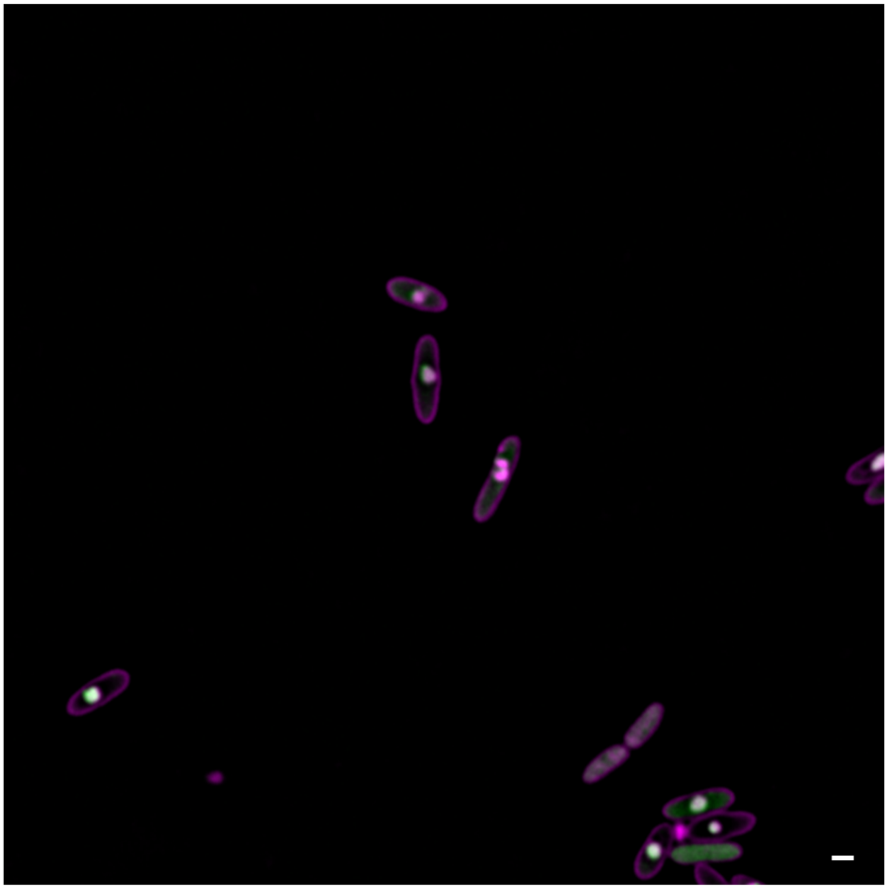

Construct: PhiPA3 gp108[PhiKZ gp104(77-95)]-sfGFP (Swap)
Phage: PhiKZ

**Figure S12 (continued)**
**Large field of view images corresponding to Figure 1 images**
Microscopy images of *P. aeruginosa* cells expressing the indicated construct and infected with the indicated phage. sfGFP signal is shown in green and DAPI signal is shown in purple. Scale bars are 1 µm.

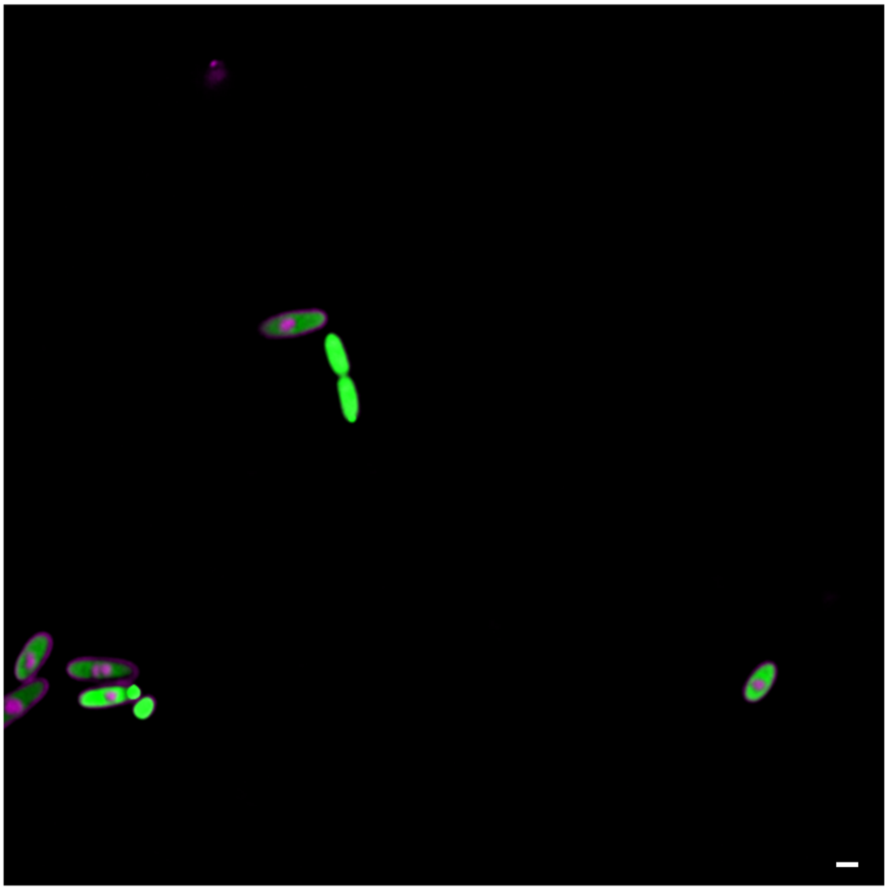

Construct: PhiPA3 gp108[PhiKZ gp104(65-76)]-sfGFP (Swap)
Phage: PhiKZ

**Figure S12 (continued)**
**Large field of view images corresponding to Figure 1 images**
Microscopy images of *P. aeruginosa* cells expressing the indicated construct and infected with the indicated phage. sfGFP signal is shown in green and DAPI signal is shown in purple. Scale bars are 1 µm.

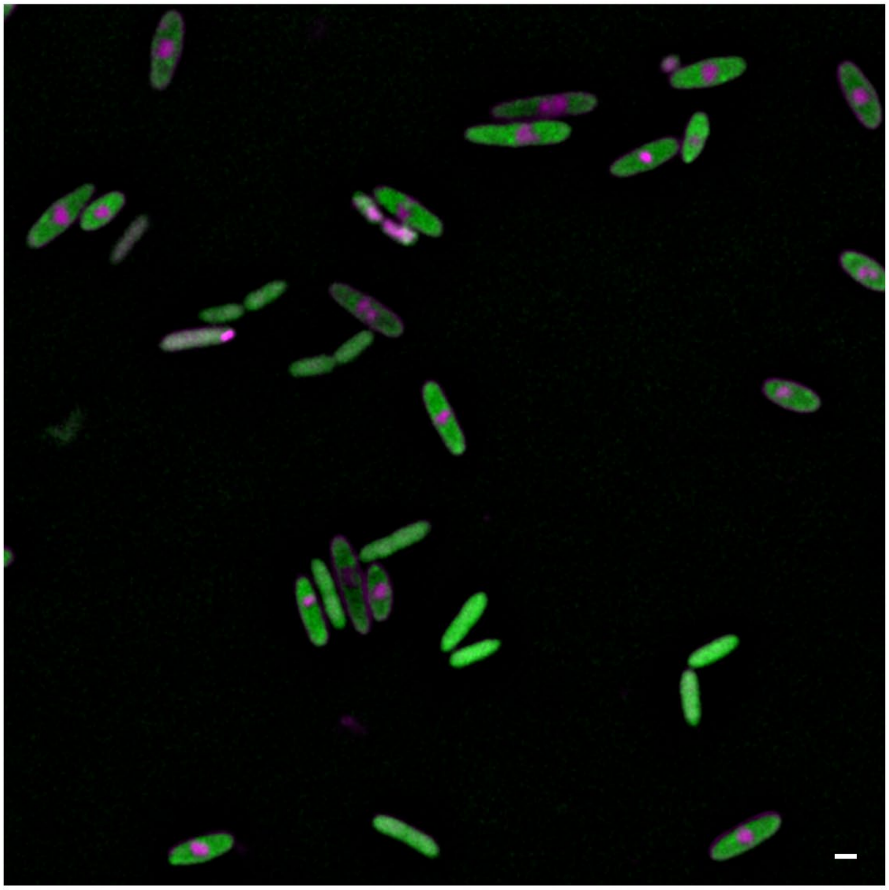

Construct: PhiPA3 gp108[PhiKZ gp104(79-88)]-sfGFP (Swap)
Phage: PhiKZ

**Figure S12 (continued)**
**Large field of view images corresponding to Figure 1 images**
Microscopy images of *P. aeruginosa* cells expressing the indicated construct and infected with the indicated phage. sfGFP signal is shown in green and DAPI signal is shown in purple. Scale bars are 1 µm.

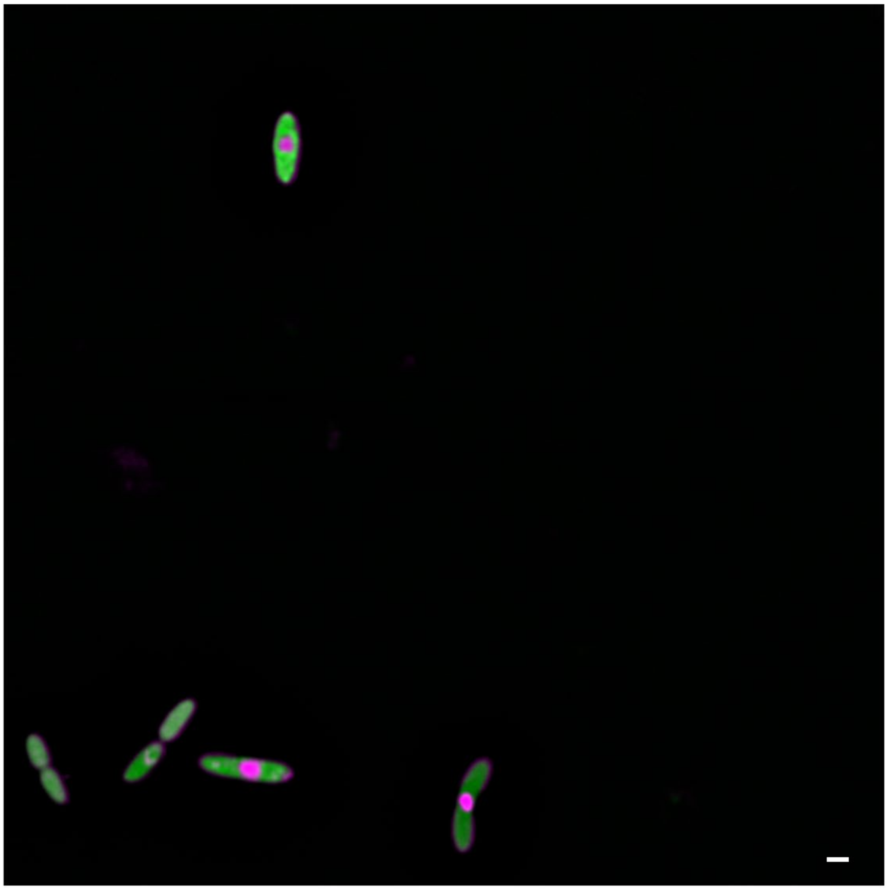

Construct: PhiPA3 gp108[PhiKZ gp104(96-115)]-sfGFP (Swap)
Phage: PhiKZ

**Figure S12 (continued)**
**Large field of view images corresponding to Figure 1 images**
Microscopy images of *P. aeruginosa* cells expressing the indicated construct and infected with the indicated phage. sfGFP signal is shown in green and DAPI signal is shown in purple. Scale bars are 1 µm.

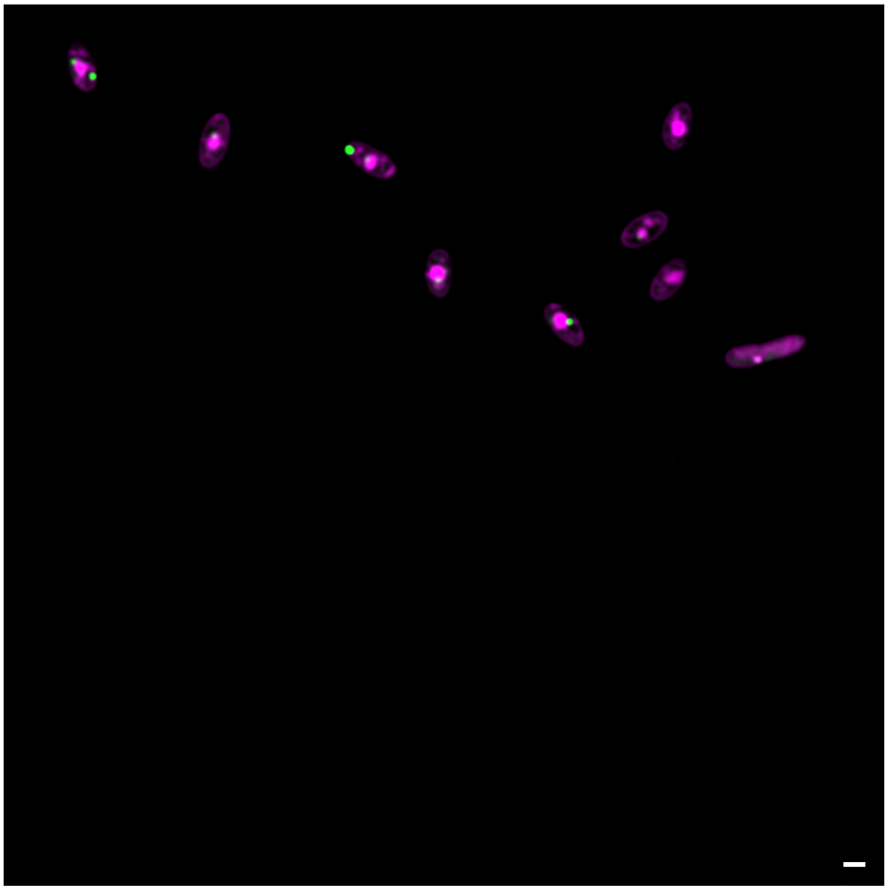

Construct: PhiPA3 gp108[PhiKZ gp104(113-162)]-sfGFP (Swap)
Phage: PhiKZ

**Figure S12 (continued)**
**Large field of view images corresponding to Figure 1 images**
Microscopy images of *P. aeruginosa* cells expressing the indicated construct and infected with the indicated phage. sfGFP signal is shown in green and DAPI signal is shown in purple. Scale bars are 1 µm.

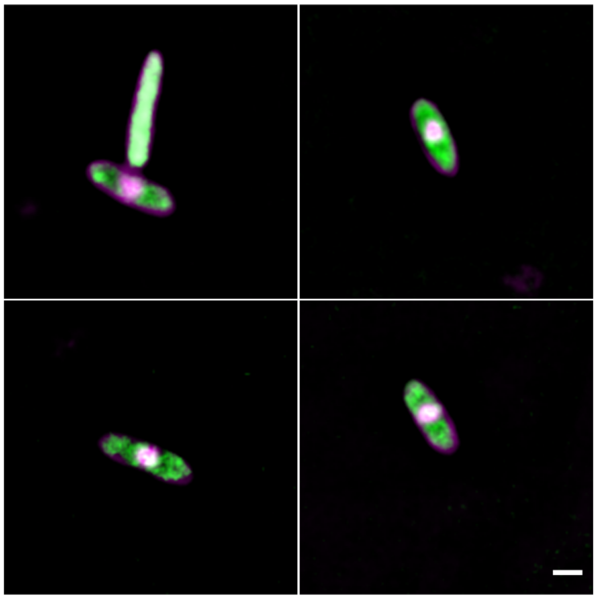

Construct: GFPmut1
Phage: PhiKZ PicA C121Y

**Figure S13**
**Large field of view images corresponding to Figure 2 images**
Microscopy images of *P. aeruginosa* cells expressing the indicated construct and infected with the indicated phage. Note that for PhiKZ C121Y infecting cells expressing GFPmut1, no single FOV contained multiple infected cells, so infected cells from different FOVs are shown. GFP signal is shown in green and DAPI signal is shown in purple. Scale bars are 1 μm.

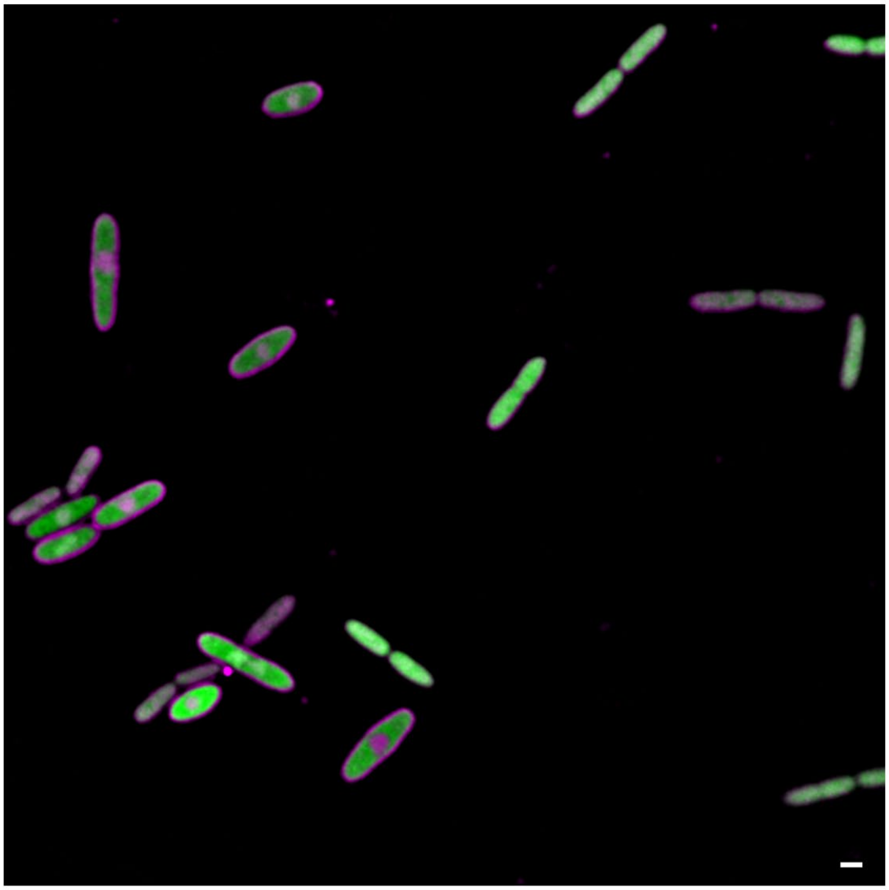

Construct: GFPmut1
Phage: PhiKZ PicA A369T

**Figure S13 (continued)**
**Large field of view images corresponding to Figure 2 images**
Microscopy images of *P. aeruginosa* cells expressing the indicated construct and infected with the indicated phage. GFP signal is shown in green and DAPI signal is shown in purple. Scale bars are 1 μm.

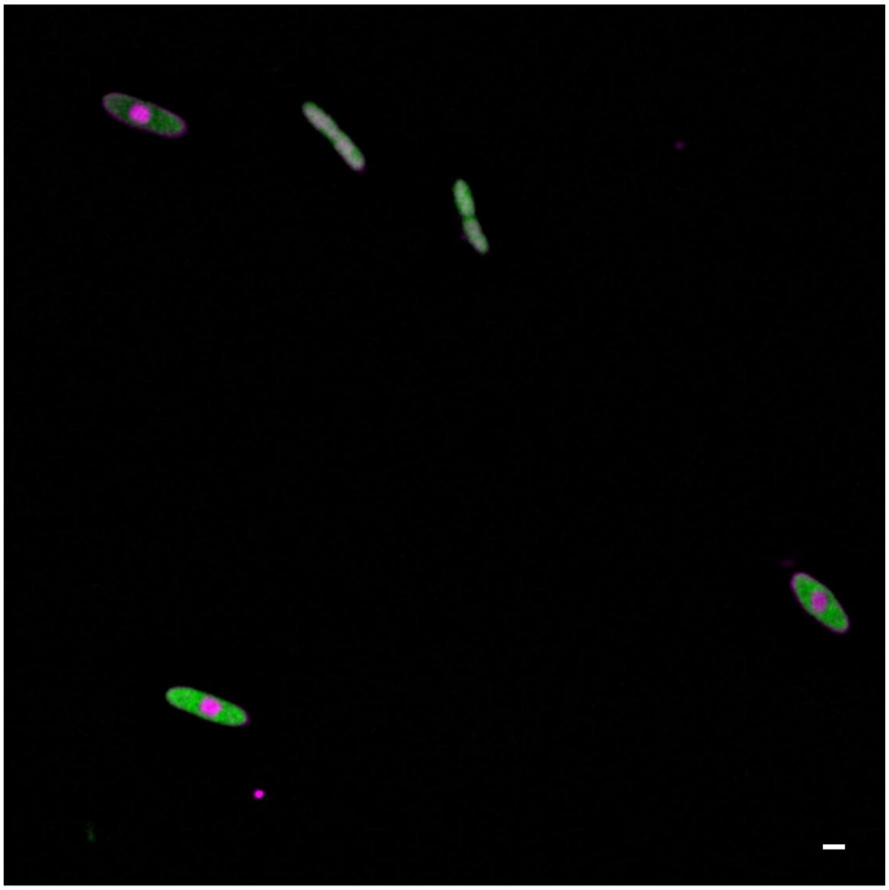

Construct: GFPmut1
Phage: PhiKZ PicA P446S

**Figure S13 (continued)**
**Large field of view images corresponding to Figure 2 images**
Microscopy images of *P. aeruginosa* cells expressing the indicated construct and infected with the indicated phage. GFP signal is shown in green and DAPI signal is shown in purple. Scale bars are 1 μm.

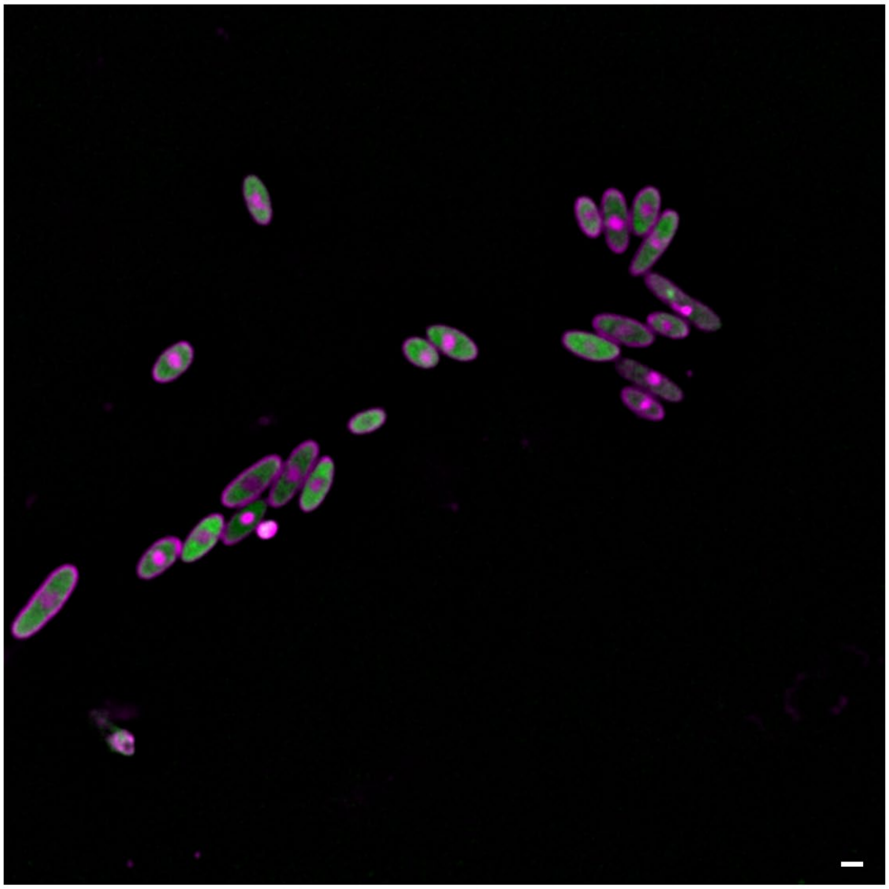

Construct: GFPmut1
Phage: PhiKZ PicA N466K

**Figure S13 (continued)**
**Large field of view images corresponding to Figure 2 images**
Microscopy images of *P. aeruginosa* cells expressing the indicated construct and infected with the indicated phage. GFP signal is shown in green and DAPI signal is shown in purple. Scale bars are 1 μm.

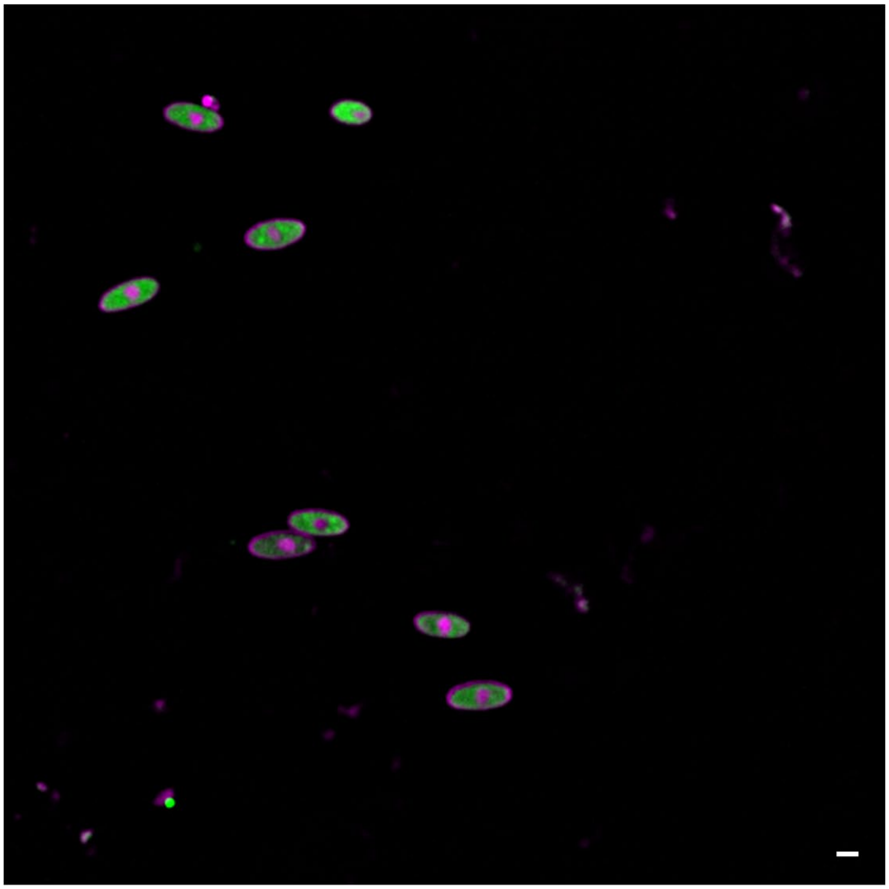

Construct: GFPmut1
Phage: PhiKZ PicA E468K

**Figure S13 (continued)**
**Large field of view images corresponding to Figure 2 images**
Microscopy images of *P. aeruginosa* cells expressing the indicated construct and infected with the indicated phage. GFP signal is shown in green and DAPI signal is shown in purple. Scale bars are 1 μm.

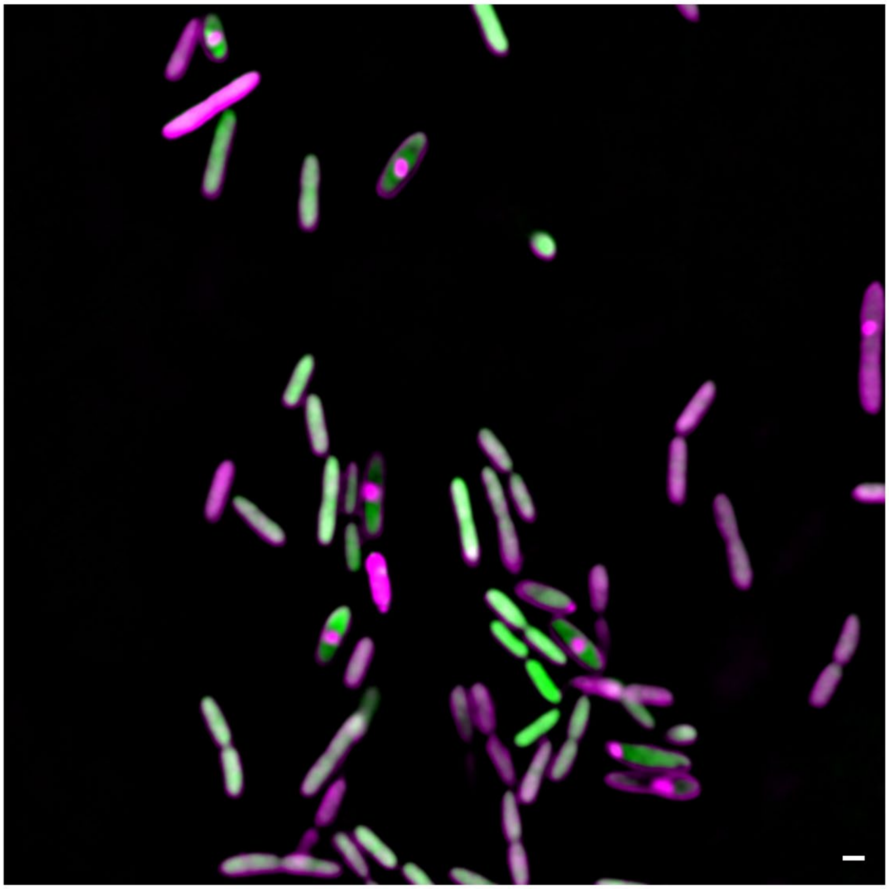

Construct: PhiPA3 gp210-GFPmut1
Phage: PhiKZ PicA C121Y

**Figure S13 (continued)**
**Large field of view images corresponding to Figure 2 images**
Microscopy images of *P. aeruginosa* cells expressing the indicated construct and infected with the indicated phage. GFP signal is shown in green and DAPI signal is shown in purple. Scale bars are 1 μm.

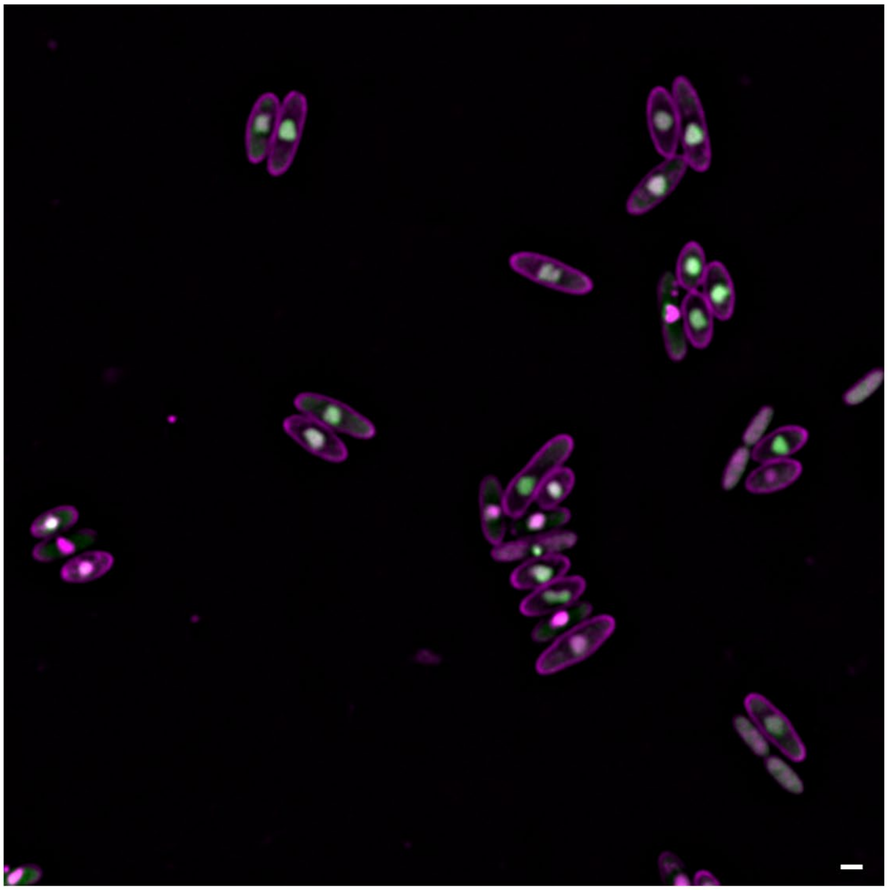

Construct: PhiKZ PicA + GFPmut1
Phage: PhiKZ PicA A369T

**Figure S13 (continued)**
**Large field of view images corresponding to Figure 2 images**
Microscopy images of *P. aeruginosa* cells expressing the indicated construct and infected with the indicated phage. GFP signal is shown in green and DAPI signal is shown in purple. Scale bars are 1 µm.

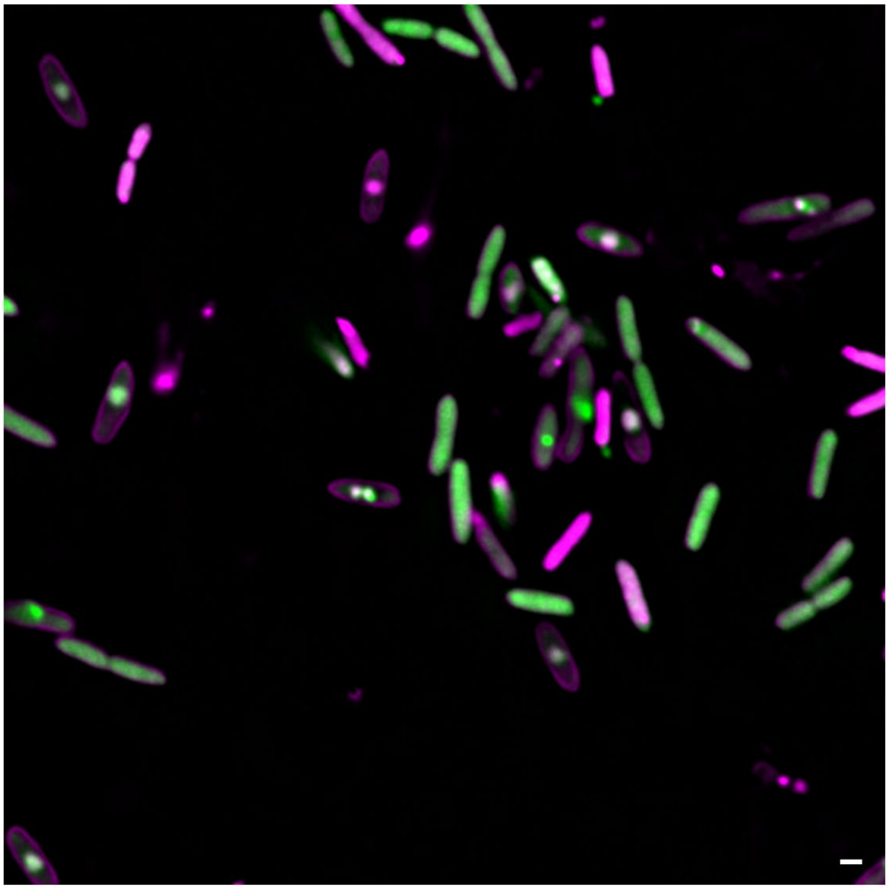

Construct: PhiKZ PicA + GFPmut1
Phage: PhiKZ PicA P446S

**Figure S13 (continued)**
**Large field of view images corresponding to Figure 2 images**
Microscopy images of *P. aeruginosa* cells expressing the indicated construct and infected with the indicated phage. GFP signal is shown in green and DAPI signal is shown in purple. Scale bars are 1 μm.

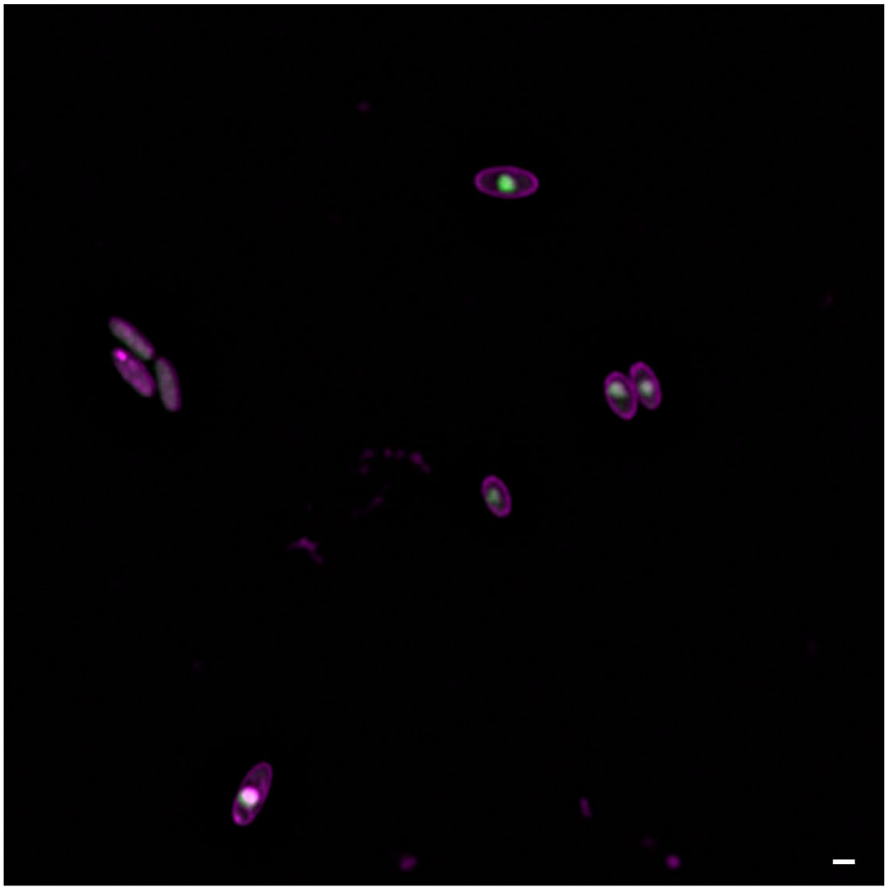

Construct: PhiKZ PicA + GFPmut1
Phage: PhiKZ PicA N466K

**Figure S13 (continued)**
**Large field of view images corresponding to Figure 2 images**
Microscopy images of *P. aeruginosa* cells expressing the indicated construct and infected with the indicated phage. GFP signal is shown in green and DAPI signal is shown in purple. Scale bars are 1 μm.

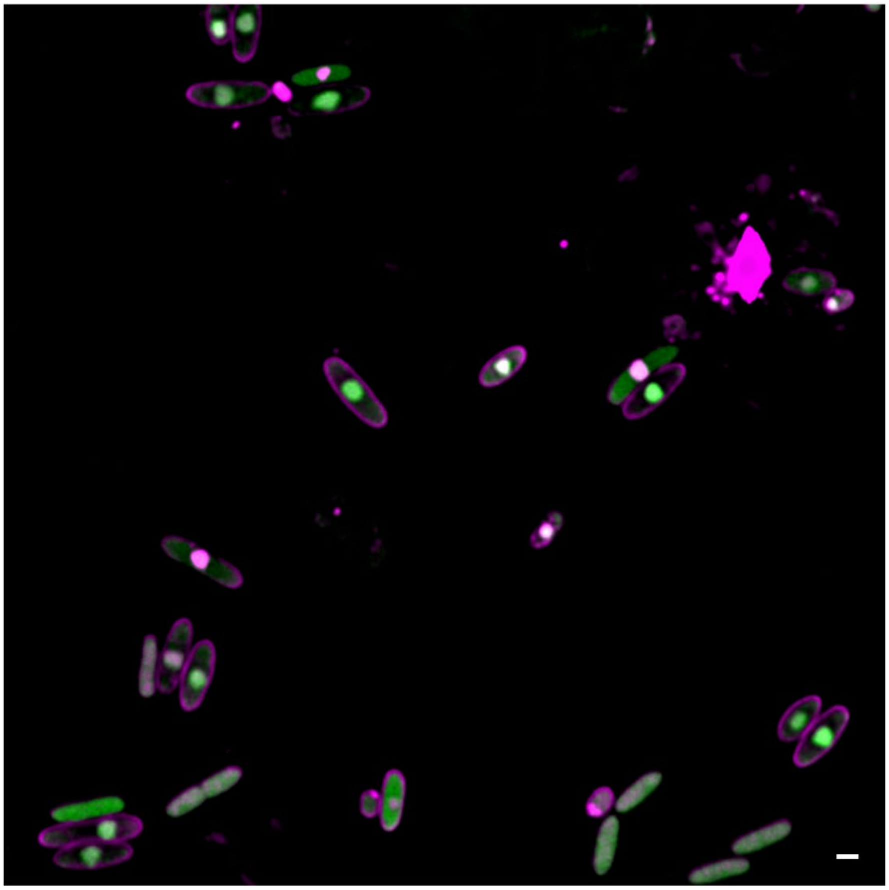

Construct: PhiKZ PicA + GFPmut1
Phage: PhiKZ PicA E468K

**Figure S13 (continued)**
**Large field of view images corresponding to Figure 2 images**
Microscopy images of *P. aeruginosa* cells expressing the indicated construct and infected with the indicated phage. GFP signal is shown in green and DAPI signal is shown in purple. Scale bars are 1 μm.

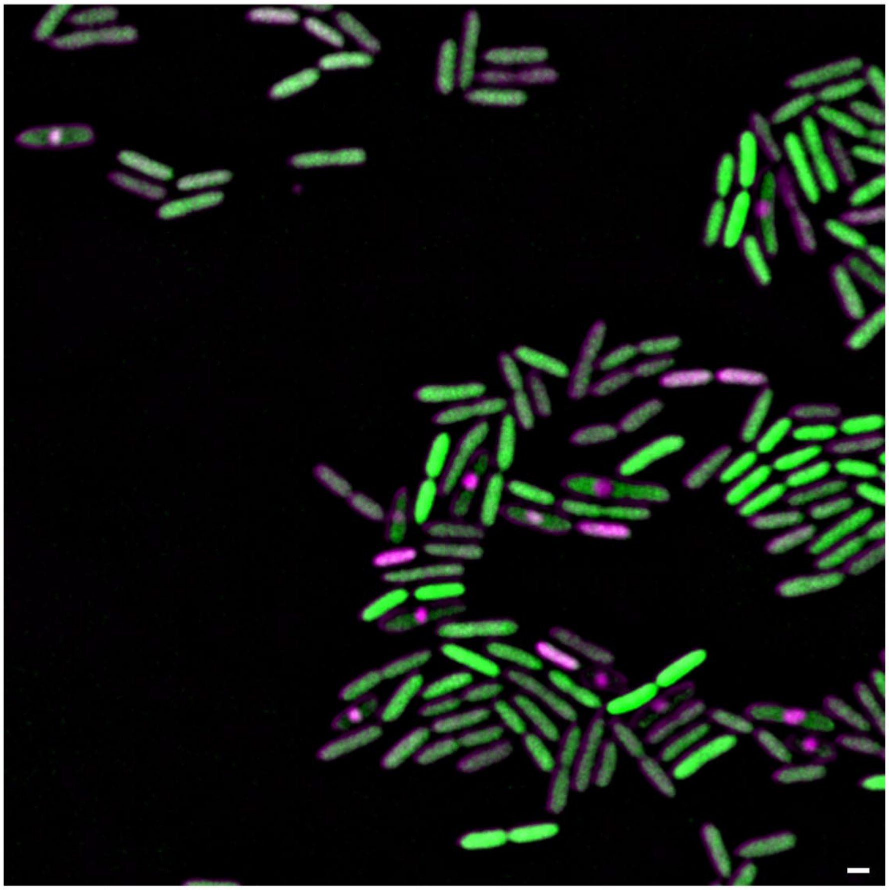

Construct: PhiKZ gp104-sfGFP
Phage: PhiKZ PicA Q297K

**Figure S14**
**Large field of view images corresponding to Figure 3 images**
Microscopy images of *P. aeruginosa* cells expressing the indicated construct and infected with the indicated phage. GFP signal is shown in green and DAPI signal is shown in purple. Scale bars are 1 µm. All infections imaged at 30-45 mpi unless otherwise noted.

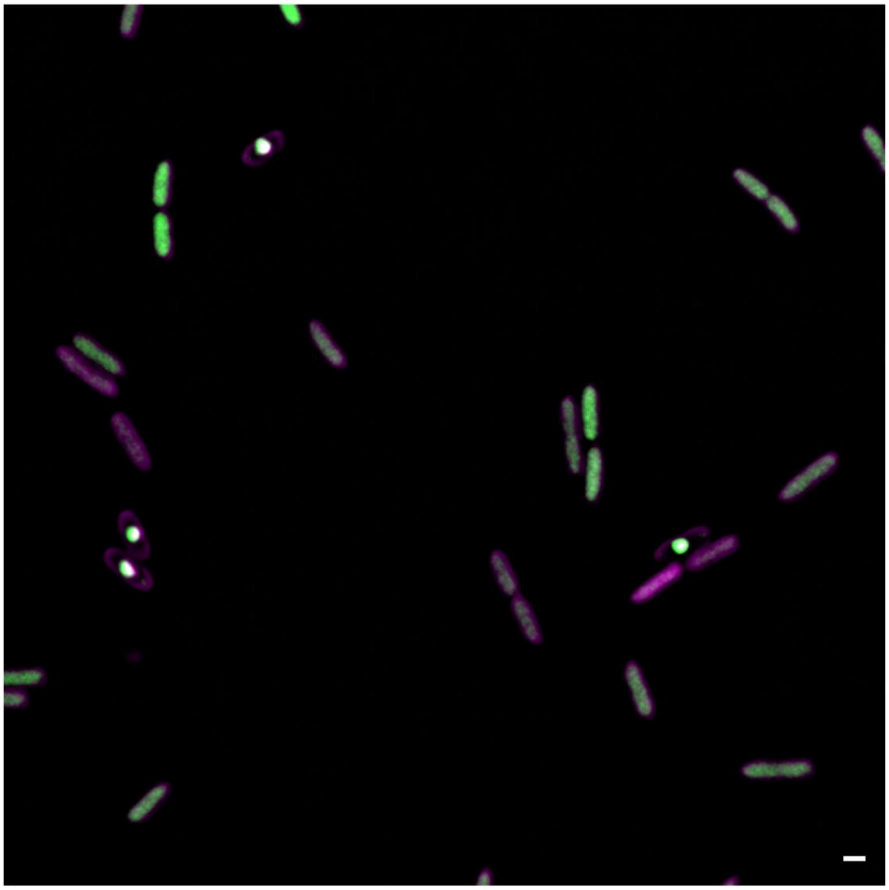

Construct: PhiKZ gp171-sfGFP
Phage: PhiKZ PicA Q297K

**Figure S14 (continued)**
**Large field of view images corresponding to Figure 3 images**
Microscopy images of *P. aeruginosa* cells expressing the indicated construct and infected with the indicated phage. GFP signal is shown in green and DAPI signal is shown in purple. Scale bars are 1 µm. All infections imaged at 30-45 mpi unless otherwise noted.

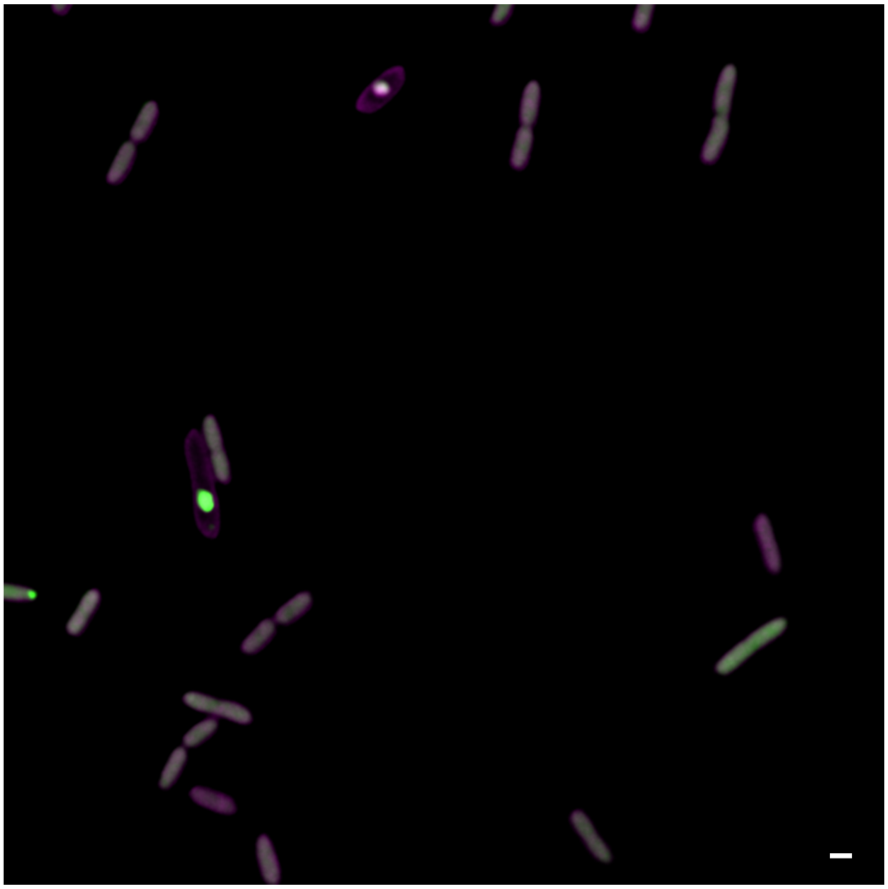

Construct: PhiKZ RecA-sfGFP
Phage: PhiKZ PicA Q297K

**Figure S14 (continued)**
**Large field of view images corresponding to Figure 3 images**
Microscopy images of *P. aeruginosa* cells expressing the indicated construct and infected with the indicated phage. GFP signal is shown in green and DAPI signal is shown in purple. Scale bars are 1 µm. All infections imaged at 30-45 mpi unless otherwise noted.

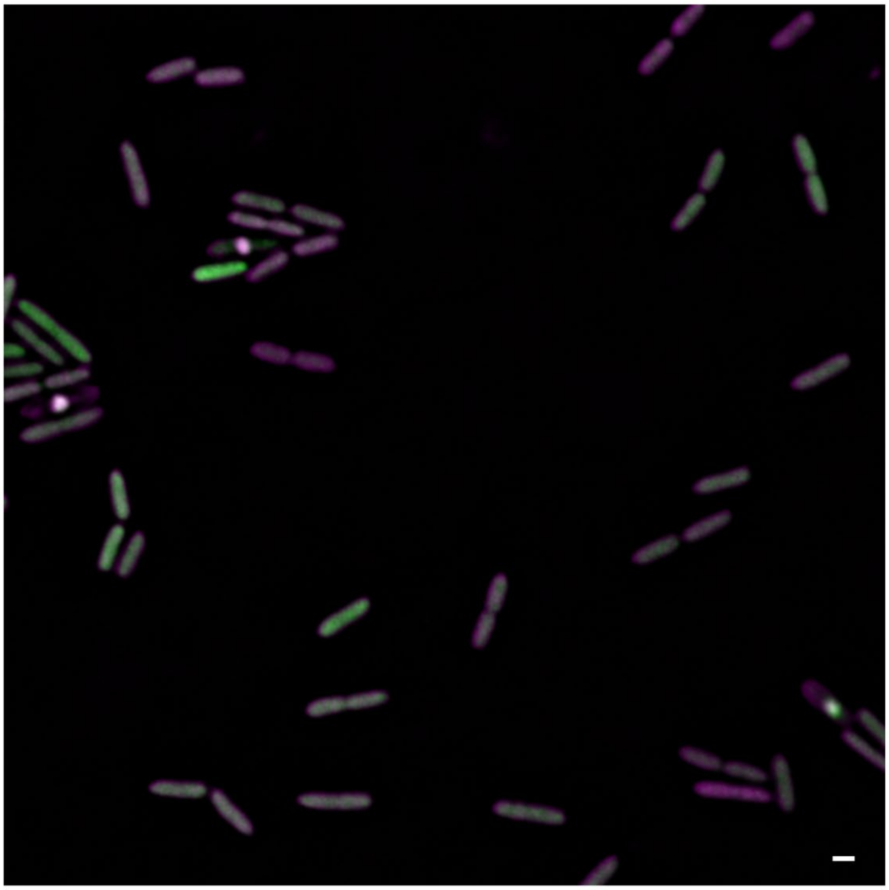

Construct: GFPmut1
Phage: PhiKZ PicA Q297K

**Figure S14 (continued)**
**Large field of view images corresponding to Figure 3 images**
Microscopy images of *P. aeruginosa* cells expressing the indicated construct and infected with the indicated phage. GFP signal is shown in green and DAPI signal is shown in purple. Scale bars are 1 µm. All infections imaged at 30-45 mpi unless otherwise noted.

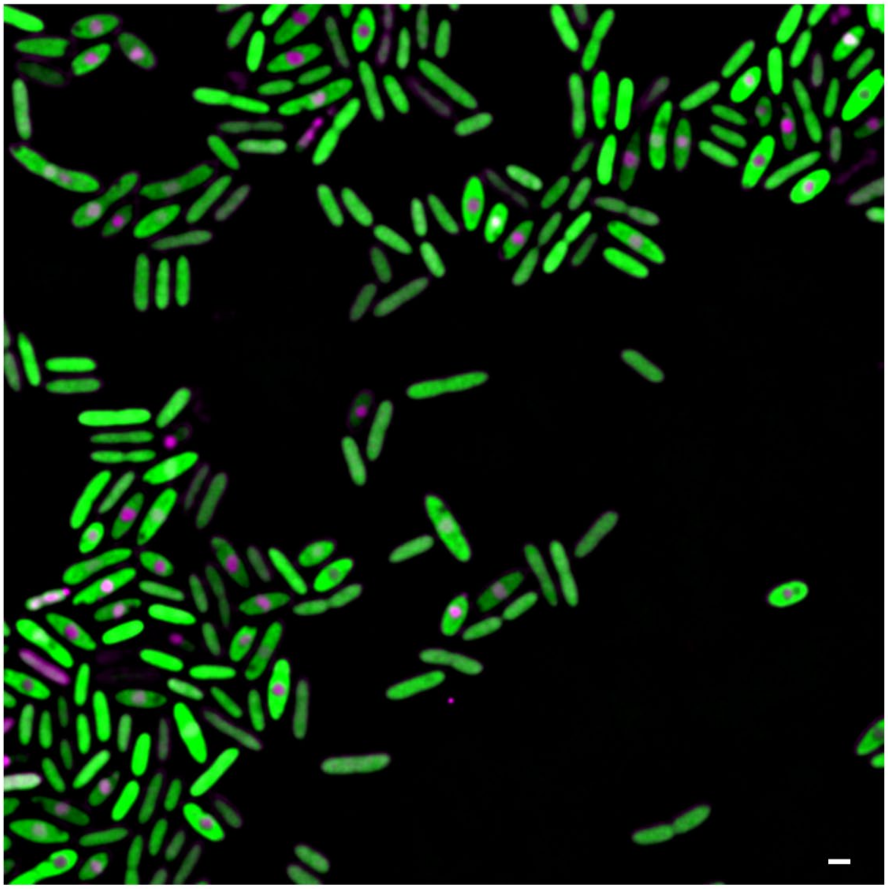

Construct: GFPmut1
Phage: PhiKZ PicA H365Q

**Figure S14 (continued)**
**Large field of view images corresponding to Figure 3 images**
Microscopy images of *P. aeruginosa* cells expressing the indicated construct and infected with the indicated phage. GFP signal is shown in green and DAPI signal is shown in purple. Scale bars are 1 μm. All infections imaged at 30-45 mpi unless otherwise noted.

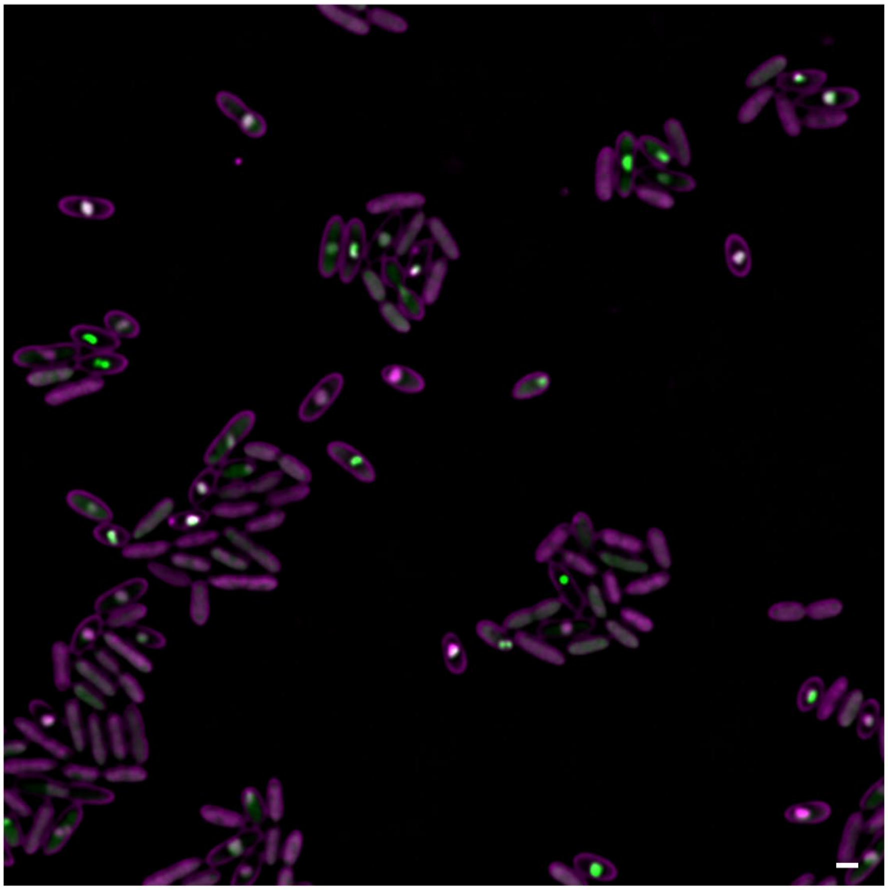

Construct: PhiKZ gp104-sfGFP
Phage: PhiKZ PicA H365Q

**Figure S14 (continued)**
**Large field of view images corresponding to Figure 3 images**
Microscopy images of *P. aeruginosa* cells expressing the indicated construct and infected with the indicated phage. GFP signal is shown in green and DAPI signal is shown in purple. Scale bars are 1 µm. All infections imaged at 30-45 mpi unless otherwise noted.

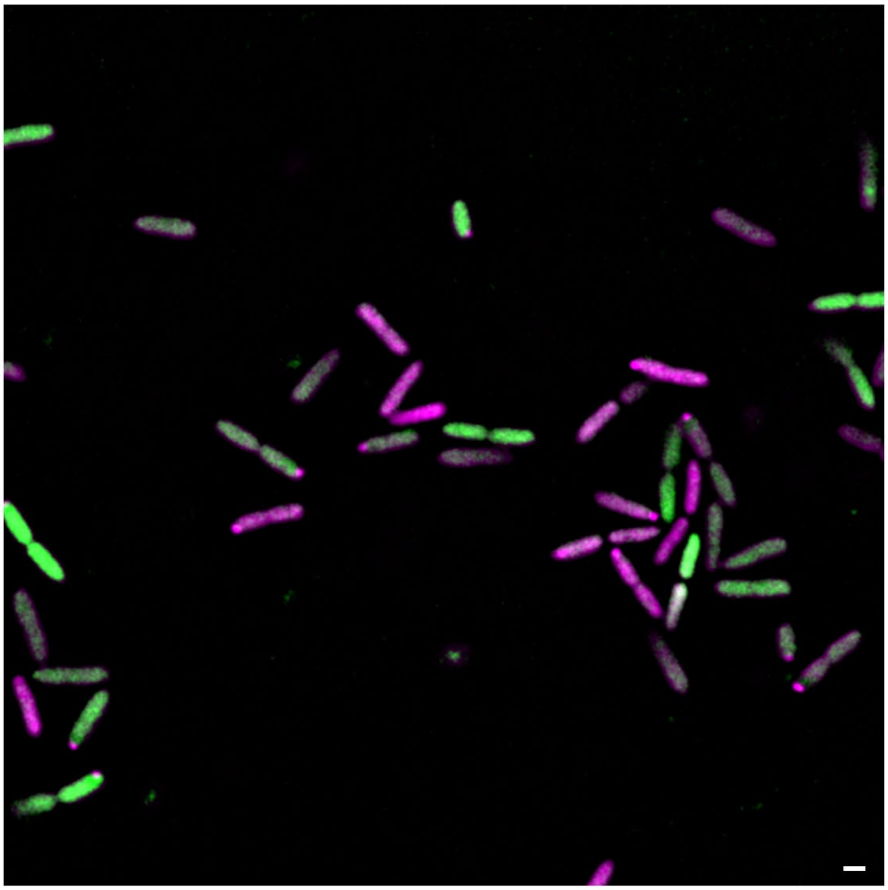

Construct: PhiKZ gp104-sfGFP
Phage: PhiKZ wildtype (5 mpi)

**Figure S14 (continued)**
**Large field of view images corresponding to Figure 3 images**
Microscopy images of *P. aeruginosa* cells expressing the indicated construct and infected with the indicated phage. GFP signal is shown in green and DAPI signal is shown in purple. Scale bars are 1 µm. All infections imaged at 30-45 mpi unless otherwise noted.

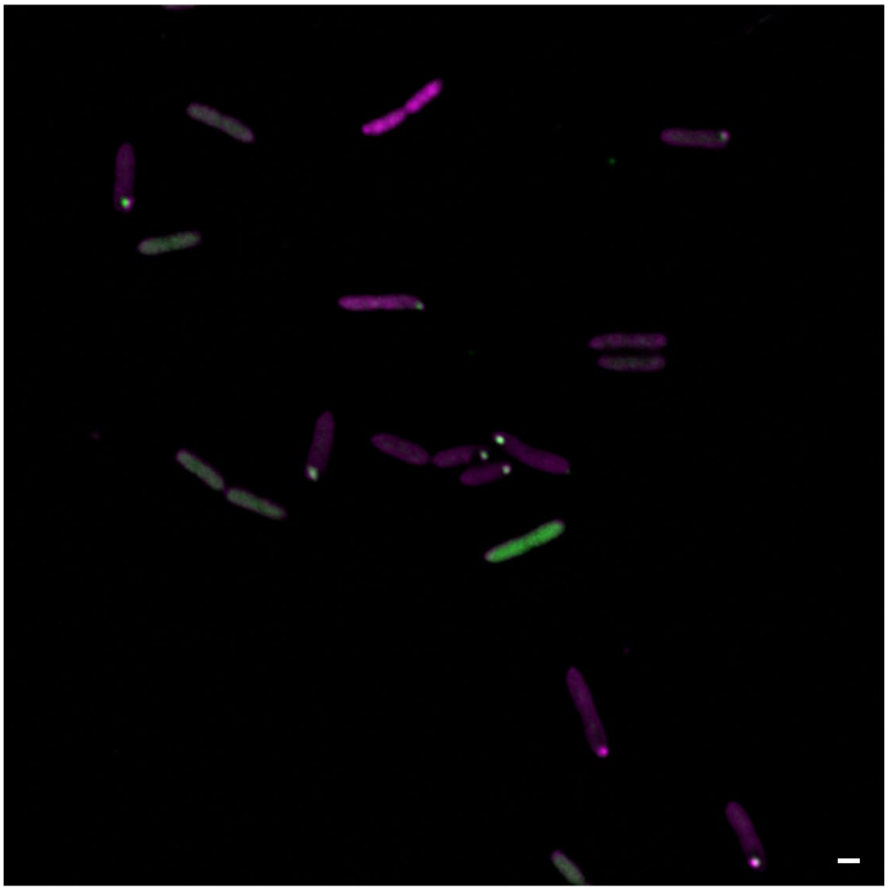

Construct: PhiKZ gp104-sfGFP
Phage: PhiKZ wildtype (10 mpi)

**Figure S14 (continued)**
**Large field of view images corresponding to Figure 3 images**
Microscopy images of *P. aeruginosa* cells expressing the indicated construct and infected with the indicated phage. GFP signal is shown in green and DAPI signal is shown in purple. Scale bars are 1 µm. All infections imaged at 30-45 mpi unless otherwise noted.

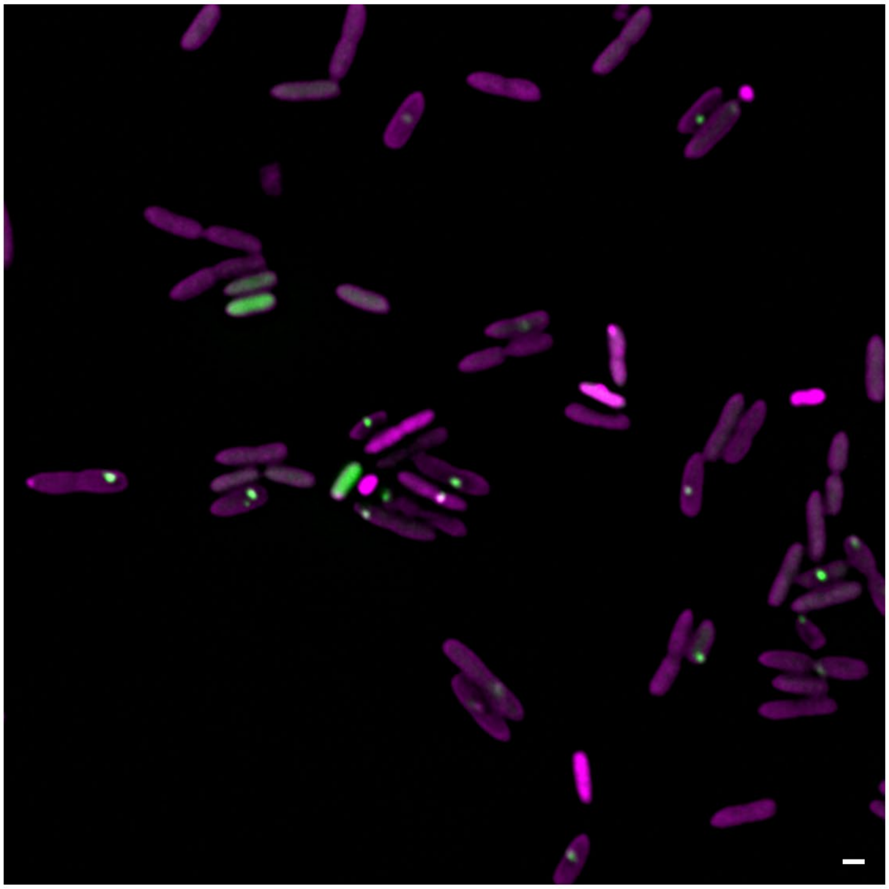

Construct: PhiKZ gp104-sfGFP
Phage: PhiKZ wildtype (15 mpi)

**Figure S14 (continued)**
**Large field of view images corresponding to Figure 3 images**
Microscopy images of *P. aeruginosa* cells expressing the indicated construct and infected with the indicated phage. GFP signal is shown in green and DAPI signal is shown in purple. Scale bars are 1 µm. All infections imaged at 30-45 mpi unless otherwise noted.

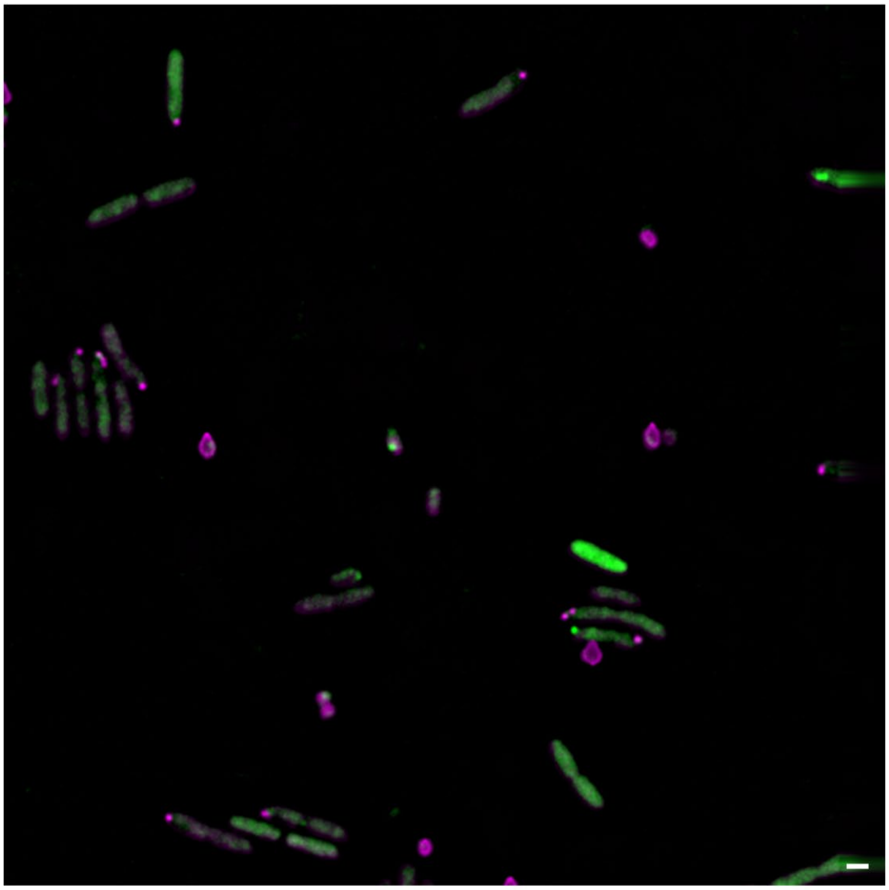

Construct: PhiKZ gp171-sfGFP
Phage: PhiKZ wildtype (5 mpi)

**Figure S14 (continued)**
**Large field of view images corresponding to Figure 3 images**
Microscopy images of *P. aeruginosa* cells expressing the indicated construct and infected with the indicated phage. GFP signal is shown in green and DAPI signal is shown in purple. Scale bars are 1 µm. All infections imaged at 30-45 mpi unless otherwise noted.

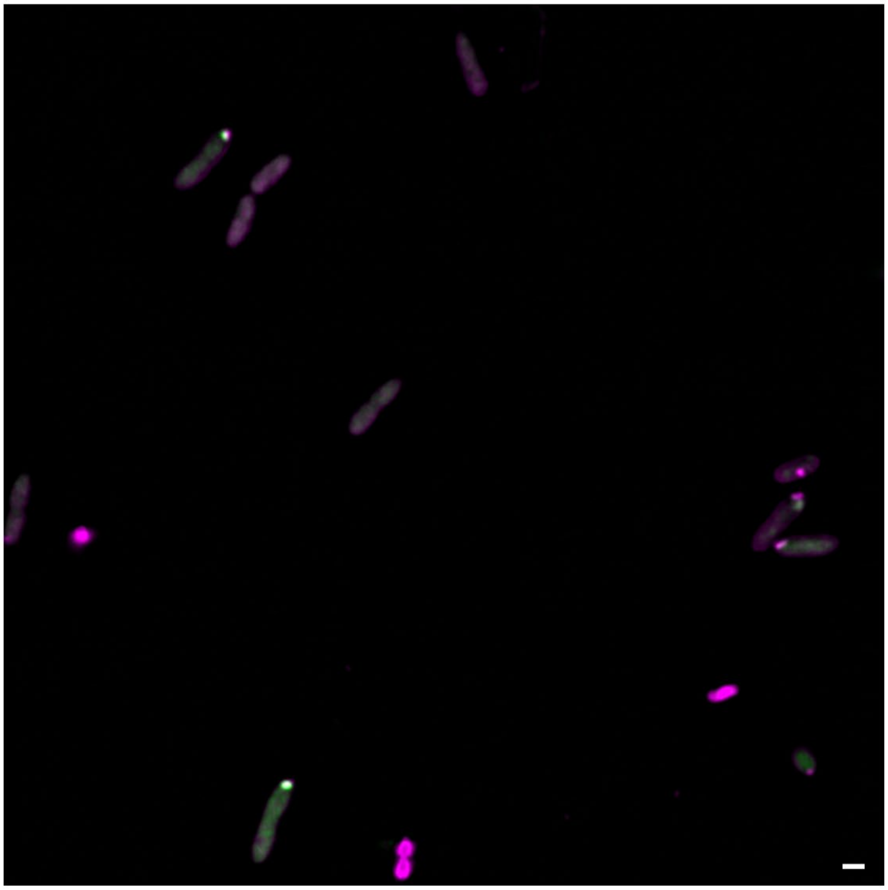

Construct: PhiKZ gp171-sfGFP
Phage: PhiKZ wildtype (10 mpi)

**Figure S14 (continued)**
**Large field of view images corresponding to Figure 3 images**
Microscopy images of *P. aeruginosa* cells expressing the indicated construct and infected with the indicated phage. GFP signal is shown in green and DAPI signal is shown in purple. Scale bars are 1 µm. All infections imaged at 30-45 mpi unless otherwise noted.

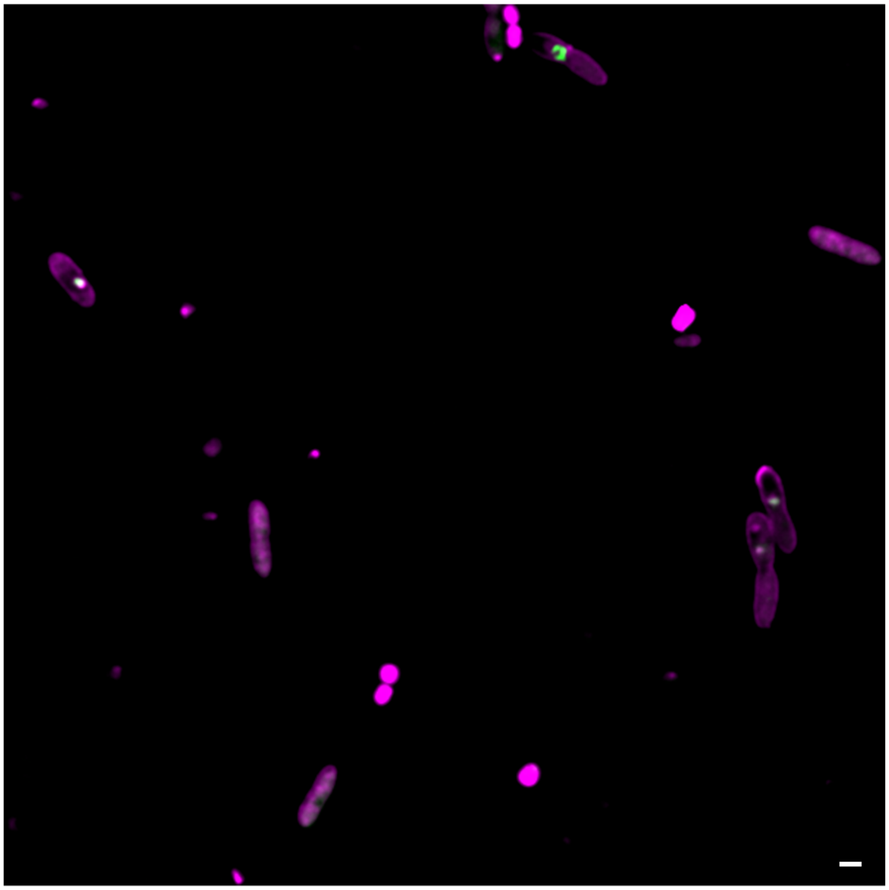

Construct: PhiKZ gp171-sfGFP
Phage: PhiKZ wildtype (15 mpi)

**Figure S14 (continued)**
**Large field of view images corresponding to Figure 3 images**
Microscopy images of *P. aeruginosa* cells expressing the indicated construct and infected with the indicated phage. GFP signal is shown in green and DAPI signal is shown in purple. Scale bars are 1 µm. All infections imaged at 30-45 mpi unless otherwise noted.

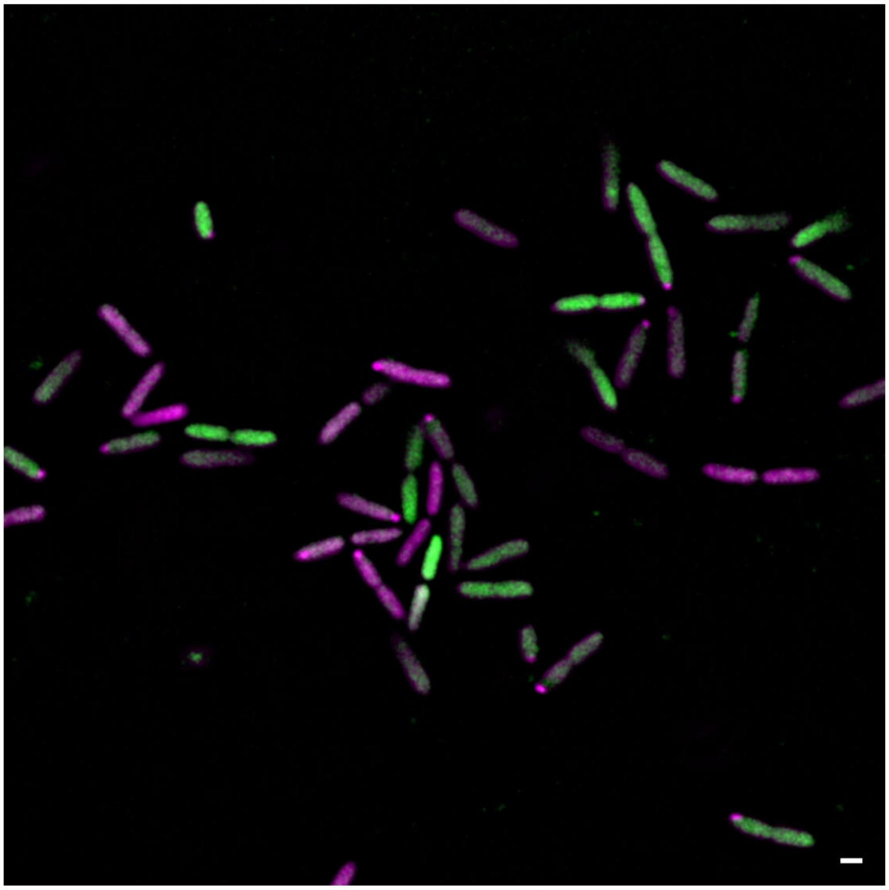

Construct: PhiKZ gp104-sfGFP
Phage: PhiKZ PicA T367P (5 mpi)

**Figure S14 (continued)**
**Large field of view images corresponding to Figure 3 images**
Microscopy images of *P. aeruginosa* cells expressing the indicated construct and infected with the indicated phage. GFP signal is shown in green and DAPI signal is shown in purple. Scale bars are 1 µm. All infections imaged at 30-45 mpi unless otherwise noted.

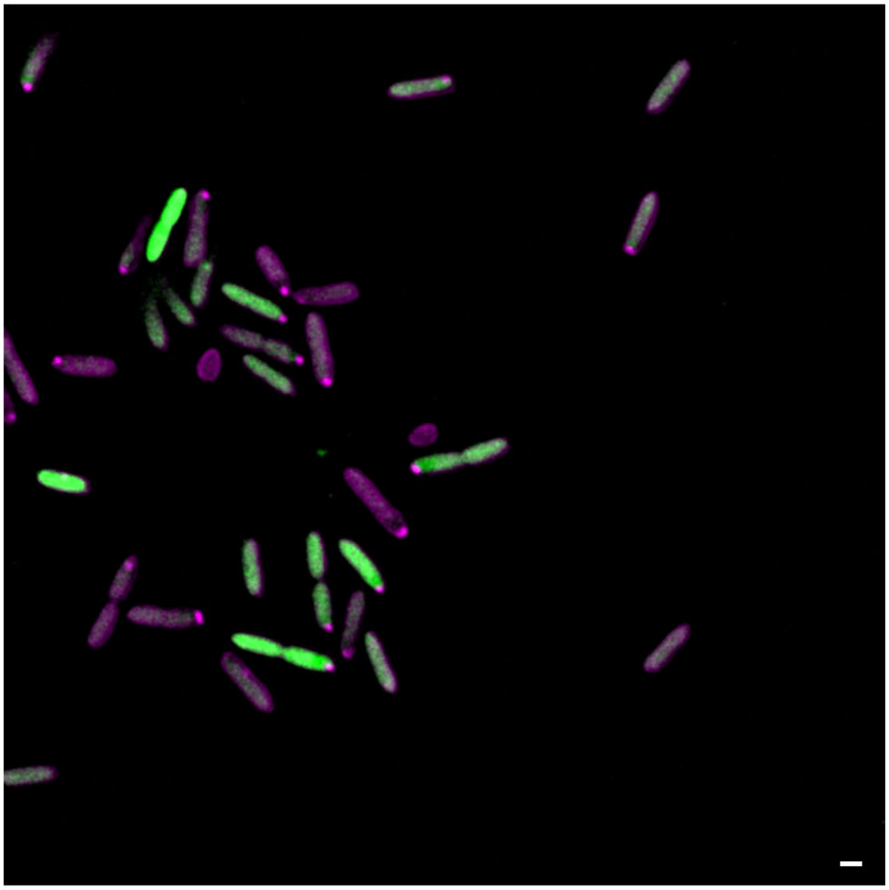

Construct: PhiKZ gp104-sfGFP
Phage: PhiKZ PicA T367P (10 mpi)

**Figure S14 (continued)**
**Large field of view images corresponding to Figure 3 images**
Microscopy images of *P. aeruginosa* cells expressing the indicated construct and infected with the indicated phage. GFP signal is shown in green and DAPI signal is shown in purple. Scale bars are 1 µm. All infections imaged at 30-45 mpi unless otherwise noted.

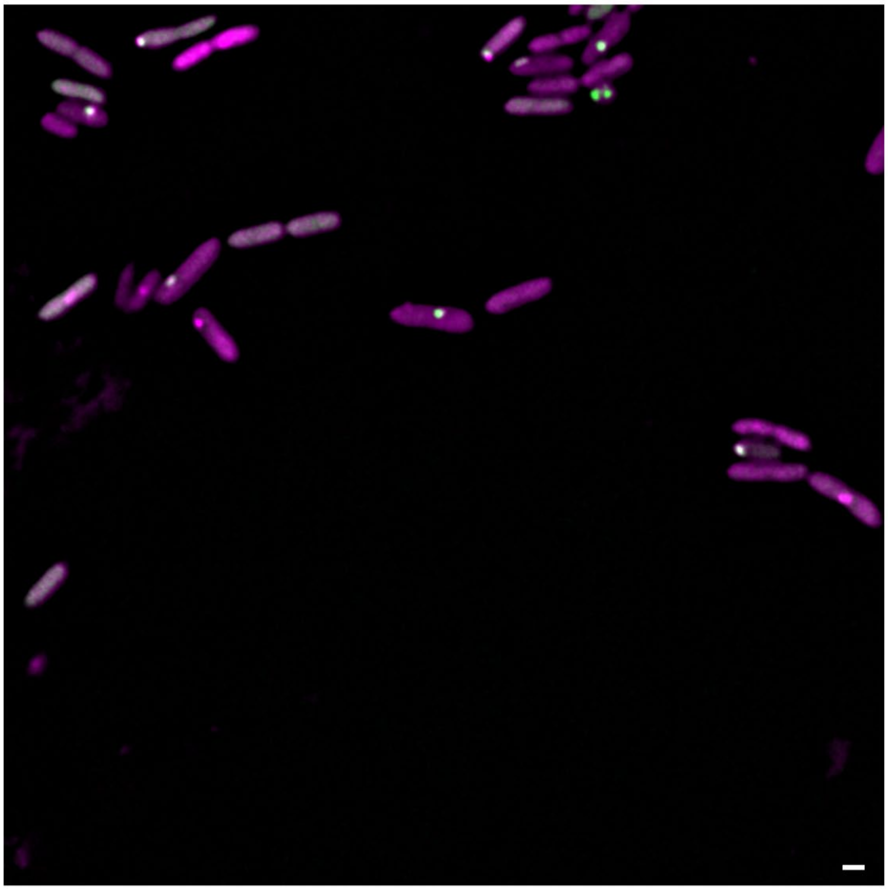

Construct: PhiKZ gp104-sfGFP
Phage: PhiKZ PicA T367P (15 mpi)

**Figure S14 (continued)**
**Large field of view images corresponding to Figure 3 images**
Microscopy images of *P. aeruginosa* cells expressing the indicated construct and infected with the indicated phage. GFP signal is shown in green and DAPI signal is shown in purple. Scale bars are 1 µm. All infections imaged at 30-45 mpi unless otherwise noted.

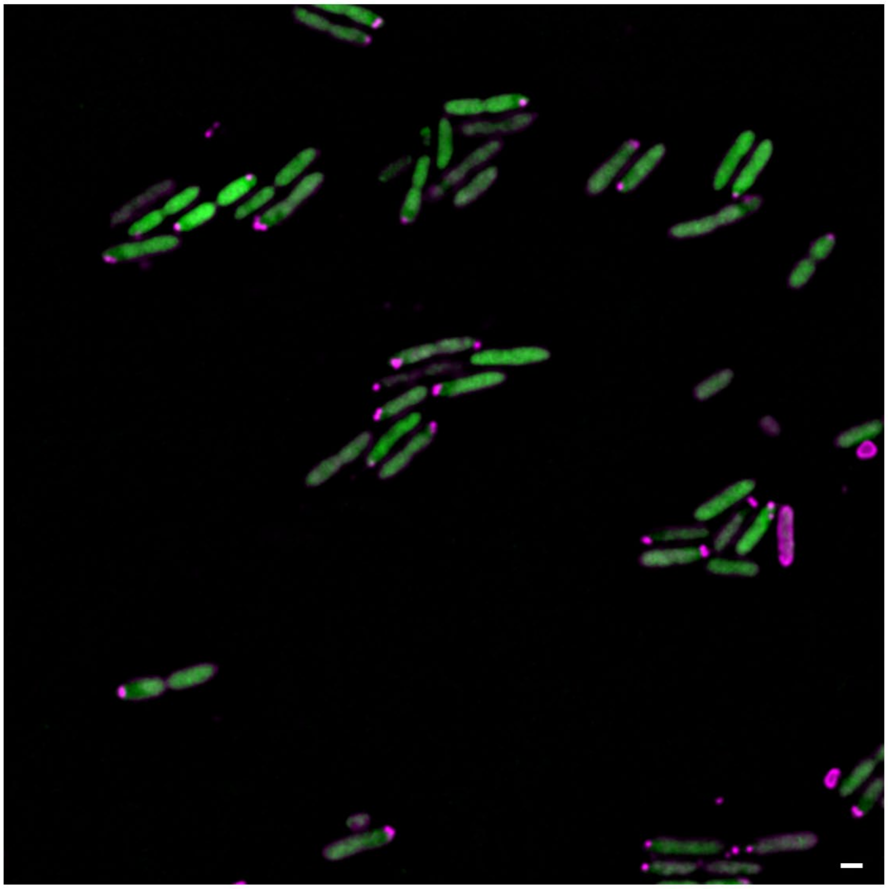

Construct: PhiKZ gp171-sfGFP
Phage: PhiKZ PicA T367P (5 mpi)

**Figure S14 (continued)**
**Large field of view images corresponding to Figure 3 images**
Microscopy images of *P. aeruginosa* cells expressing the indicated construct and infected with the indicated phage. GFP signal is shown in green and DAPI signal is shown in purple. Scale bars are 1 µm. All infections imaged at 30-45 mpi unless otherwise noted.

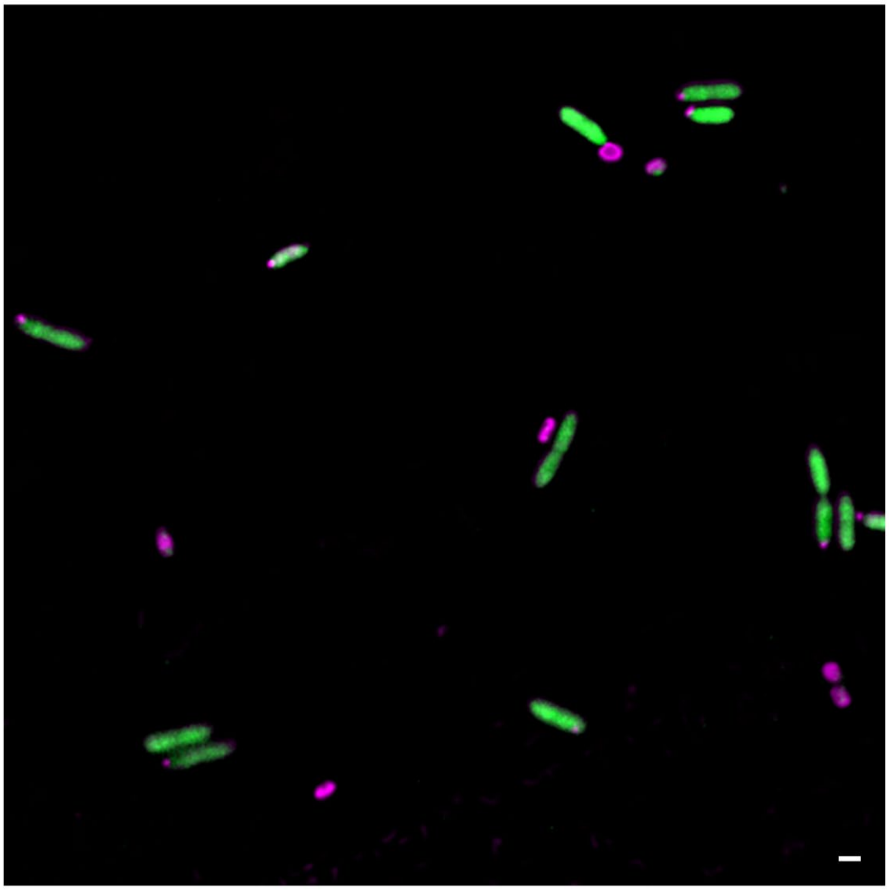

Construct: PhiKZ gp171-sfGFP
Phage: PhiKZ PicA T367P (10 mpi)

**Figure S14 (continued)**
**Large field of view images corresponding to Figure 3 images**
Microscopy images of *P. aeruginosa* cells expressing the indicated construct and infected with the indicated phage. GFP signal is shown in green and DAPI signal is shown in purple. Scale bars are 1 µm. All infections imaged at 30-45 mpi unless otherwise noted.

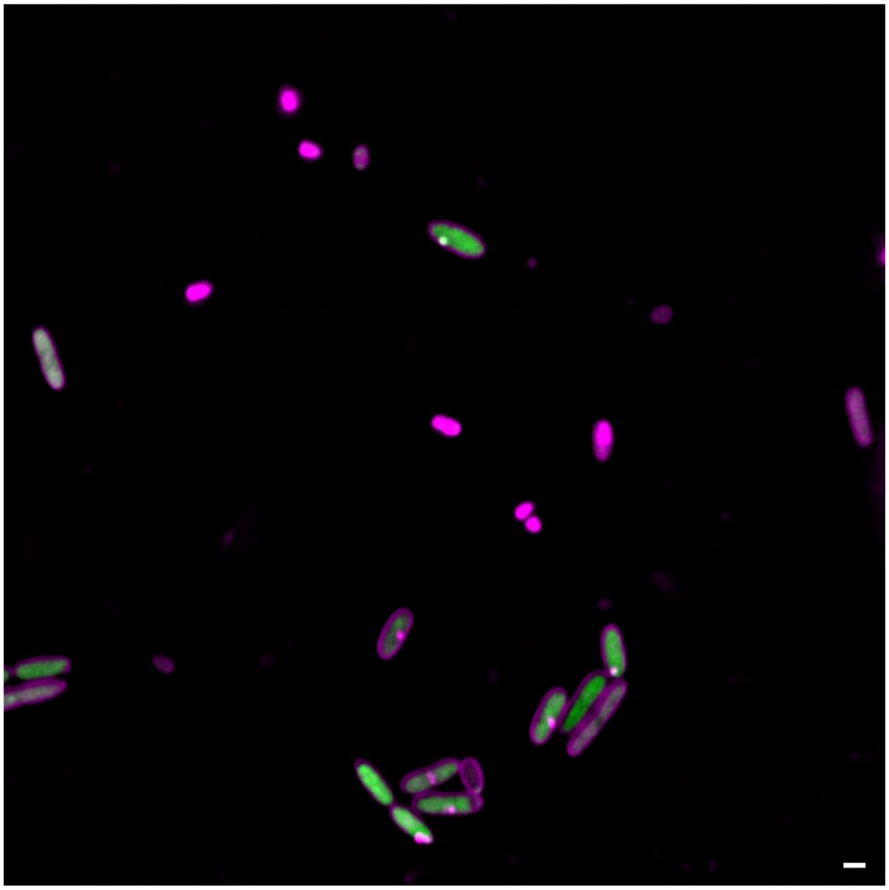

Construct: PhiKZ gp171-sfGFP
Phage: PhiKZ PicA T367P (15 mpi)

**Figure S14 (continued)**
**Large field of view images corresponding to Figure 3 images**
Microscopy images of *P. aeruginosa* cells expressing the indicated construct and infected with the indicated phage. GFP signal is shown in green and DAPI signal is shown in purple. Scale bars are 1 µm. All infections imaged at 30-45 mpi unless otherwise noted.

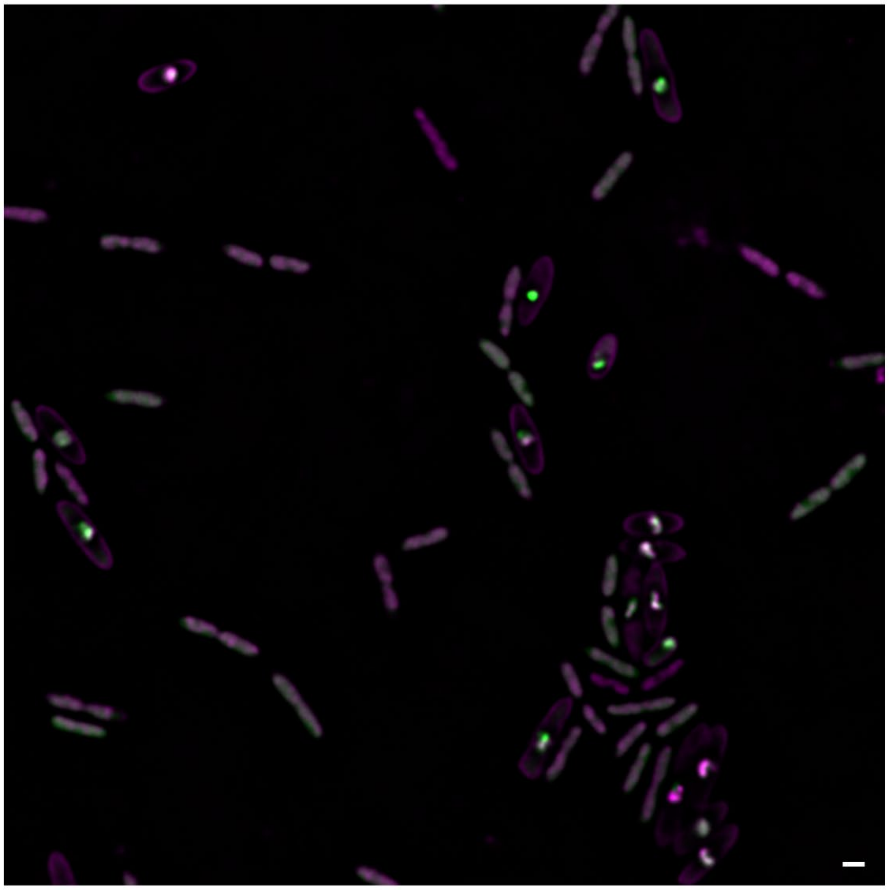

Construct: PhiKZ gp171-sfGFP
Phage: PhiKZ PicA T367P (30 mpi)

**Figure S14 (continued)**
**Large field of view images corresponding to Figure 3 images**
Microscopy images of *P. aeruginosa* cells expressing the indicated construct and infected with the indicated phage. GFP signal is shown in green and DAPI signal is shown in purple. Scale bars are 1 µm. All infections imaged at 30-45 mpi unless otherwise noted.

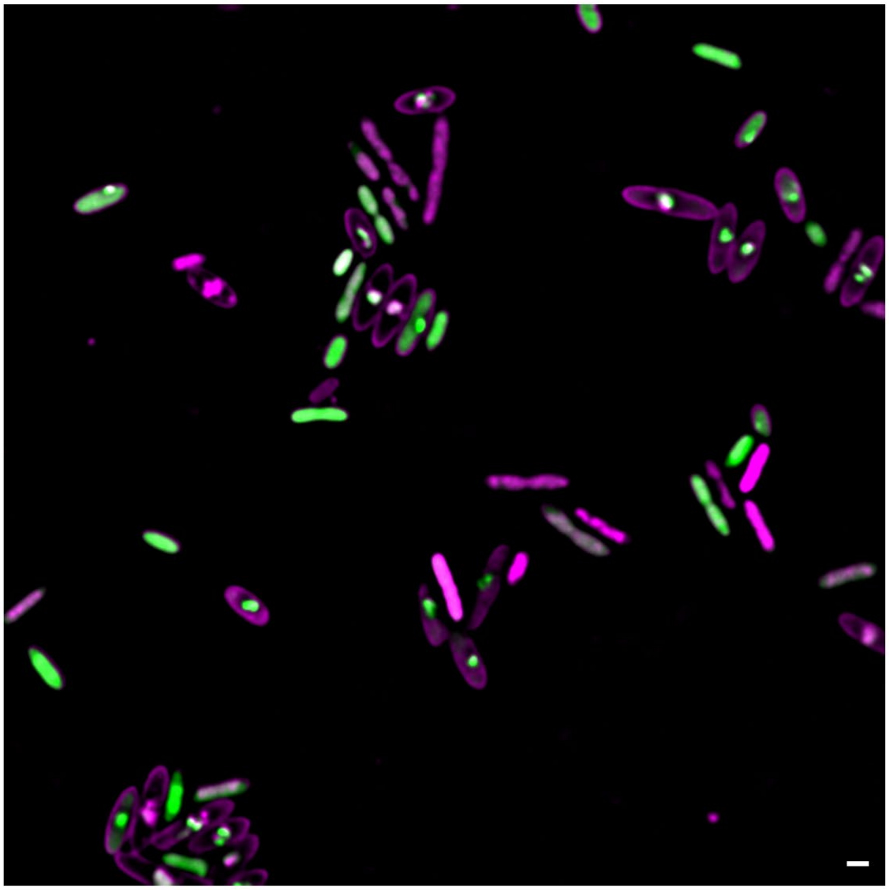

Construct: PhiKZ gp104-sfGFP
Phage: PhiKZ PicA T367P (30 mpi)

**Figure S14 (continued)**
**Large field of view images corresponding to Figure 3 images**
Microscopy images of *P. aeruginosa* cells expressing the indicated construct and infected with the indicated phage. GFP signal is shown in green and DAPI signal is shown in purple. Scale bars are 1 µm. All infections imaged at 30-45 mpi unless otherwise noted.

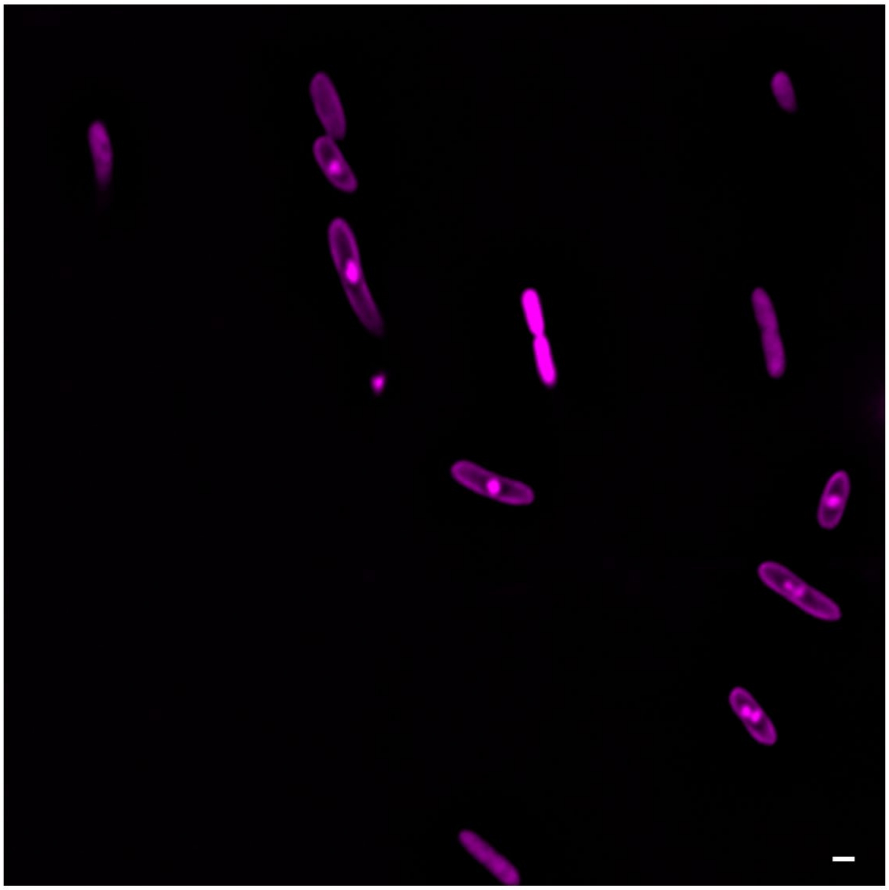

Construct: None
Phage: PhiKZ wildtype (30 mpi)

**Figure S14 (continued)**
**Large field of view images corresponding to Figure 3 images**
Microscopy images of *P. aeruginosa* cells expressing the indicated construct and infected with the indicated phage. GFP signal is shown in green and DAPI signal is shown in purple. Scale bars are 1 µm. All infections imaged at 30-45 mpi unless otherwise noted.

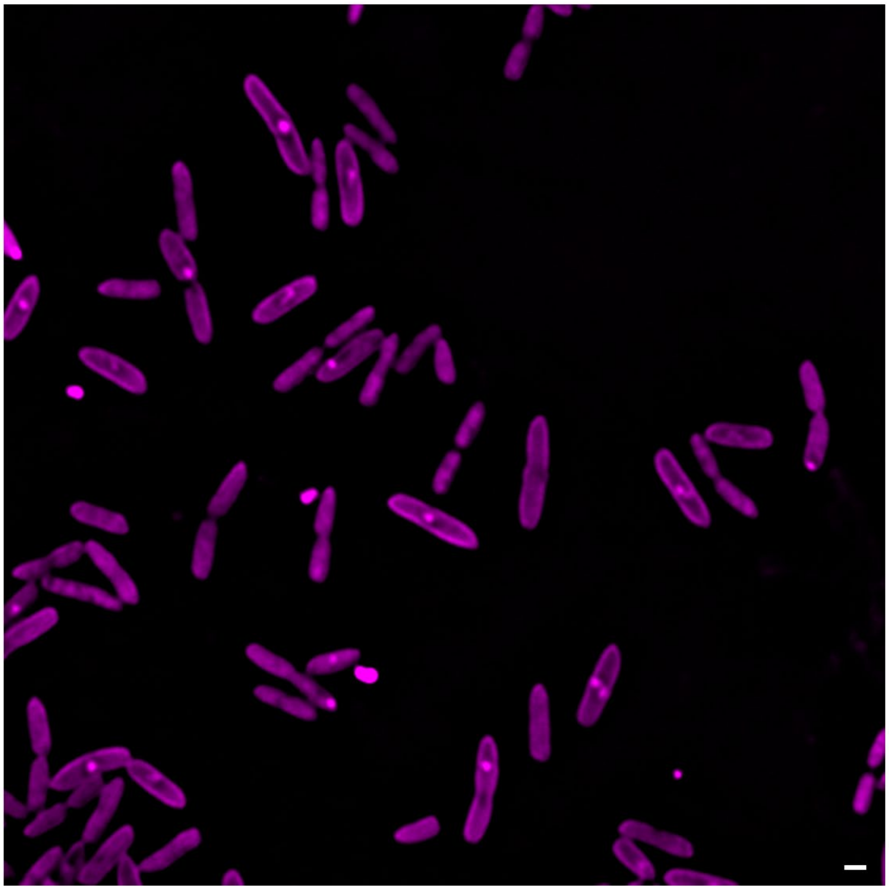

Construct: None
Phage: PhiKZ PicA T367P (30 mpi)

**Figure S14 (continued)**
**Large field of view images corresponding to Figure 3 images**
Microscopy images of *P. aeruginosa* cells expressing the indicated construct and infected with the indicated phage. GFP signal is shown in green and DAPI signal is shown in purple. Scale bars are 1 µm. All infections imaged at 30-45 mpi unless otherwise noted.

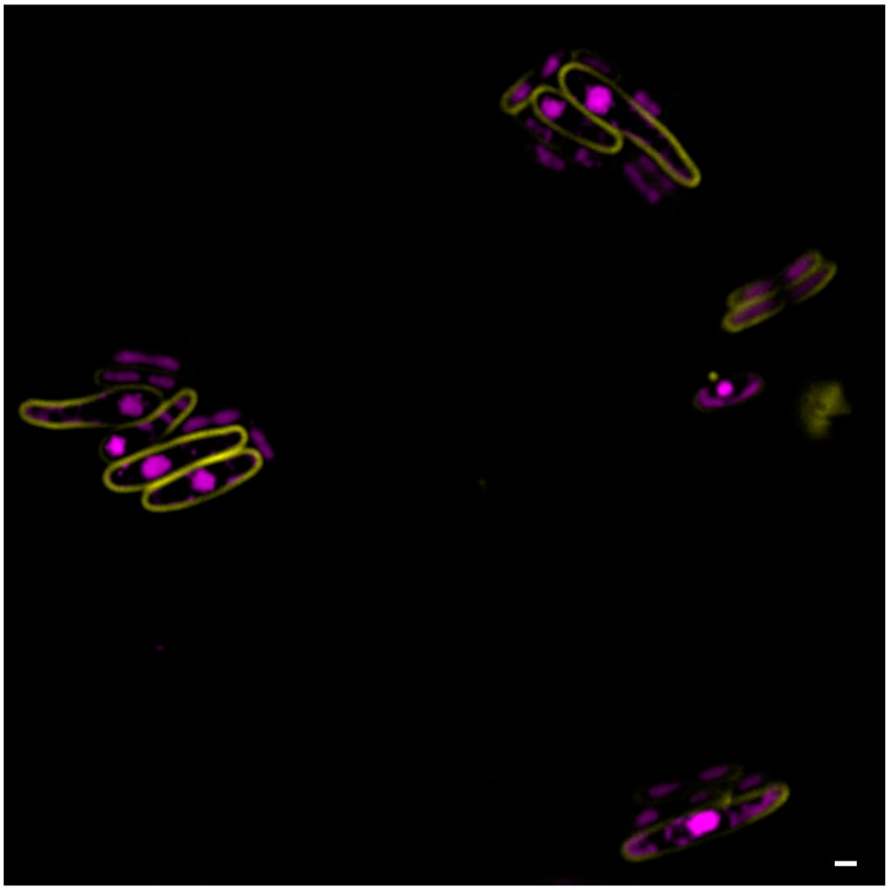

Construct: dCas13d Empty Vector
Phage: Goslar

**Figure S15**
**Large field of view images corresponding to Figure 4 images**
Microscopy images of *E. coli* cells expressing the indicated construct and infected with the indicated phage. DAPI signal is shown in purple and FM4-64 signal is shown in yellow. Scale bars are 1 µm. All images taken at 75-90 mpi.

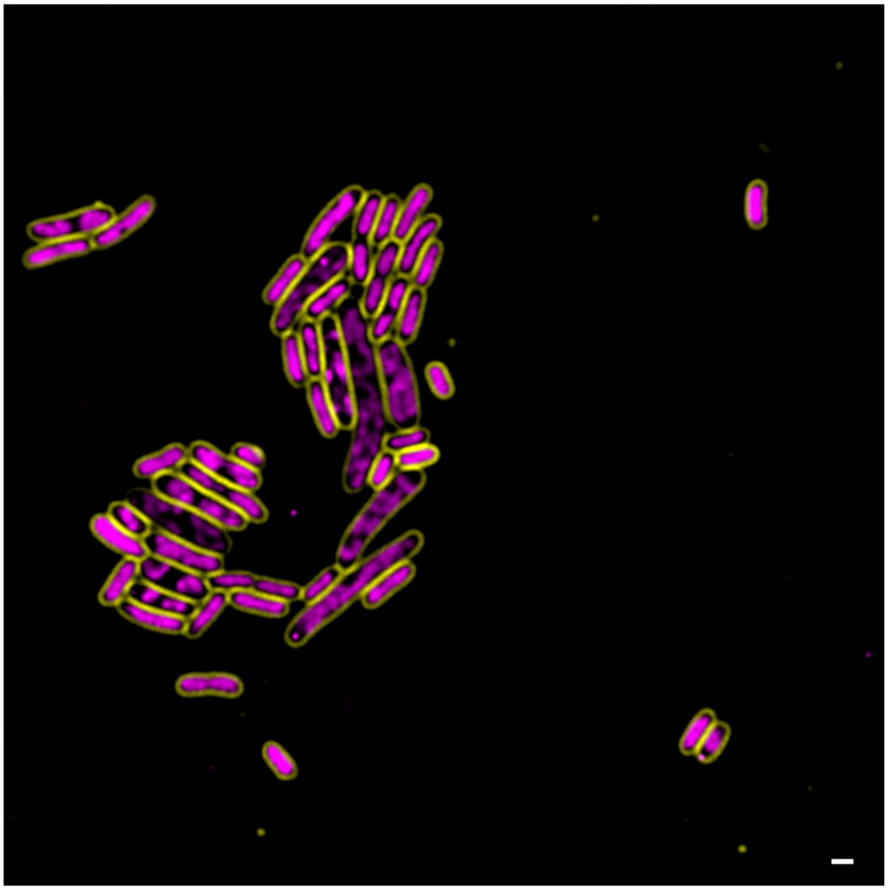

Construct: dCas13d Goslar PicA g1
Phage: Goslar

**Figure S15 (continued)**
**Large field of view images corresponding to Figure 4 images**
Microscopy images of *E. coli* cells expressing the indicated construct and infected with the indicated phage. DAPI signal is shown in purple and FM4-64 signal is shown in yellow. Scale bars are 1 µm. All images taken at 75-90 mpi.

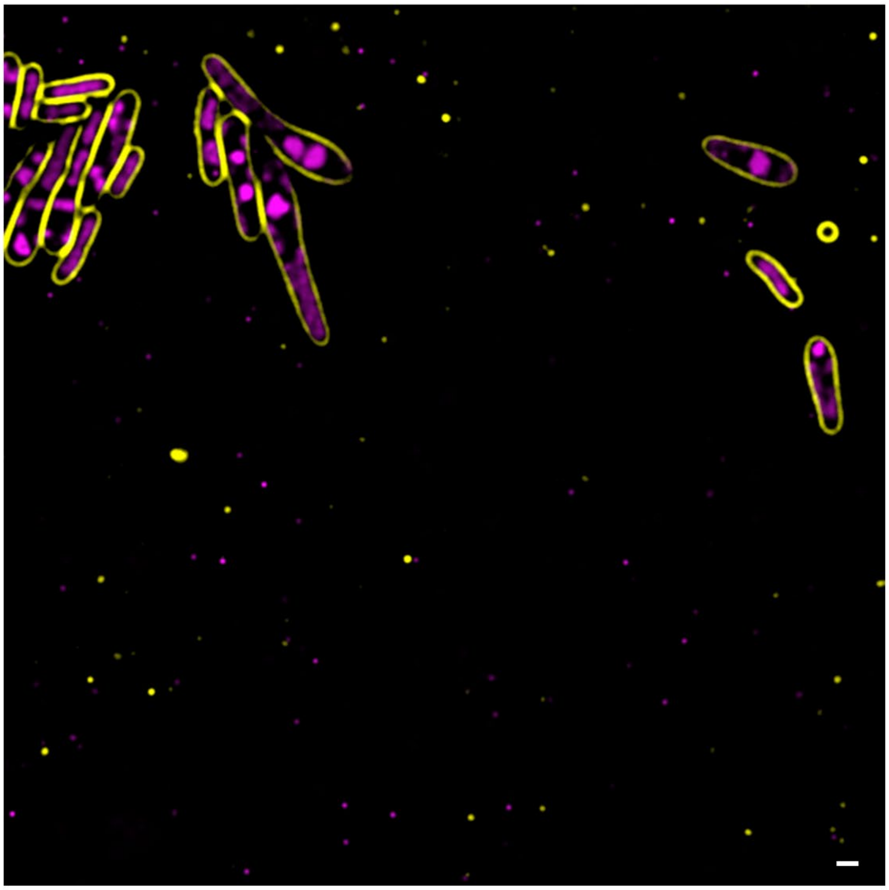

Construct: dCas13d Goslar PicA g1 + codon altered PicA
Phage: Goslar

**Figure S15 (continued)**
**Large field of view images corresponding to Figure 4 images**
Microscopy images of *E. coli* cells expressing the indicated construct and infected with the indicated phage. DAPI signal is shown in purple and FM4-64 signal is shown in yellow. Scale bars are 1 µm. All images taken at 75-90 mpi.
